# Evaluation of canine 2D cell cultures as models of myxomatous mitral valve degeneration

**DOI:** 10.1371/journal.pone.0221126

**Published:** 2019-08-15

**Authors:** Karen Tan, Greg Markby, Rhona Muirhead, Rachel Blake, Lisa Bergeron, Greg Fici, Kim Summers, Vicky Macrae, Brendan Corcoran

**Affiliations:** 1 Roslin Institute, University of Edinburgh, Roslin, United Kingdom; 2 Royal (Dick) School of Veterinary Studies, University of Edinburgh, Roslin, United Kingdom; 3 Zoetis Animal Health, Kalamazoo, Michigan, United States of America; Scuola Superiore Sant’Anna, ITALY

## Abstract

The utility of cells cultured from the mitral valve as models of myxomatous diseases needs to be properly validated. In this study valve interstitial cells (VICs) and valve endothelial cells (VECs) were cultured from normal and diseased canine mitral valves in 2% (v/v) or 10% FBS media, in the presence of TGFβ1, 2 and 3, the TGFβ RI kinase inhibitor SB431542 and TGFβ neutralising antibodies, 5HT and the 5HT2RB antagonist LY272015. Cultures were examined by morphology, transcriptomic profiling, protein expression of the cell specific markers αSMA and SM22α (VICs), and CD31 (VECs), deposition of proteoglycans (PG), the PG versican, and the TGFβs themselves. VECs derived from normal valves were CD31+/αSMA-, but those from diseased valves were αSMA+, indicating endothelial-to-mesenchymal (EndoMT) transition had occurred. The TGFβs induced EndoMT in normal VECs, and this was abolished by SB431542, with significant changes in αSMA, CD31 and HAS2 expression (P<0.05). Normal VICs cultured in 10% FBS media were αSMA+ (activated myofibroblast (disease) phenotype), but were αSMA- when grown in 2% FBS. VICs from diseased dogs were αSMA+ in 2% FBS (retention of the activated myofibroblast disease phenotype), with significantly increased TGFβ1 expression (P<0.05) compared to normal cells. Treatment of normal and diseased VICs with the TGFβs significantly increased expression of αSMA, SM22α, versican, the TGFβs themselves, and deposition of PGs (P<0.05), with TGFβ1 being the most potent activator. These effects were either abolished or markedly reduced by SB431542 and a pan-TGFβ neutralizing antibody (P<0.05). SB431542 also markedly reduced αSMA expression in VICs from diseased valves, but 5HT and LY272015 had no effect on VIC phenotype. Transcriptomic profiling identified clear differences in gene expression for the different conditions and treatments that partially matched that seen in native diseased valve tissue, including changes in expression of *ACTA2* (αSMA), *5HTR2B*, *TAGLN* (SM22α) and *MYH10* (SMemb), gene ontology terms and canonical signalling pathways. Normal and diseased VICs and normal VECs from canine mitral valves can be successfully grown in culture with retention of phenotype, which can be manipulated using TGFβ1 and the TGFβ RI kinase inhibitor SB431542. This optimized cell system can now be used to model MMVD to elucidate disease mechanisms and identify key regulators of disease progression.

## Introduction

Myxomatous mitral valve degeneration (MMVD) is the single most common acquired cardiovascular disease of the dog, and an important pathological component of a range of valvulopathies in humans, including Barlow’s Disease and Fibroelastic Deficiency, making the dog a potentially useful naturally-occurring large animal model for acquired human mitral valvulopathies [1–4]. Key pathological changes of myxomatous degeneration in both species involve the extracellular matrix (ECM) with progressive loss and disorganization of collagen bundles and elastin fibres and the accumulation of proteoglycans (PGs) and glycosaminoglycans (GAGs) [5–7].

The pathogenesis of MMVD is only partially understood. Loss of mitral valve endothelial cells (VECs), endothelial-to-mesenchymal transition (EndoMT) and transition of normally quiescent valvular interstitial cells (qVICs) into activated myofibroblasts (aVICs) likely contribute to the ECM changes seen [1, 8–12]. Changes in a number of signalling pathways have been reported including the TGFβ/BMP super-family, 5-hydroxytryptamine (5HT, serotonin), angiotensin and Wnt/β-catenin [13–19]. EndoMT has been shown to be activated in canine MMVD and a sheep model where VECs lose expression of CD31 (PECAM1, platelet and endothelial cell adhesion molecule 1) and CDH5 (cadherin 5), and gain αSMA expression, and transcriptomic data would suggest this also occurs in human MMVD [12]. αSMA is also a marker for activated myofibroblasts. Diseased canine valves have increasing numbers of αSMA+ myofibroblasts and TGFβ1 mediates αSMA+ myofibroblast transformation in cultured VICs [11, 20–22].

TGFβ signalling appears to be the prominent pathway implicated in MMVD [14, 21, 23, 24]. Canonical SMAD-dependent TGFβ pathway activation and up-regulation found in human MMVD possibly reflects end-stage fibrosis, but transcriptomic evidence from the dog suggests involvement of non-canonical TGFβ signaling pathways, and this may well be the case in the early non-fibrotic stage of human MMVD [14, 16–18, 25–29]. A role for 5HT signaling in MMVD is also suspected [30, 31]. With TGFβ, 5HT may be important in ECM regulation, and while consistent up-regulation of the *5HTR2A* and *5HTR2B* 5HT receptor genes in MMVD has been reported, 5HT itself does not increase expression of αSMA in normal VICs [14, 16, 19, 23, 30, 31]. Mechanical stimulation of tissue engineered valves (human) does induce *5HTR2A*, *5HTR2B* and *ACTA2* (αSMA) gene expression and these effects can be blocked by the 5HTR2B antagonist LY272015 [31].

Culture of canine mitral valve cells has been reported, but none are reliable *in vitro* models of MMVD due to spontaneous increased expression of αSMA in both VICs and VECs when cultured in standard culture media containing 10%FBS v/v [32–34]. In conventional monocultures with 10% FBS, normal VECs spontaneously undergo EndoMT and qVICs have an αSMA+ activated myofibroblastic (aVIC) phenotype, which hampers comparison between normal and diseased states, and this clearly limits options for functional and mechanistic studies of pathogenesis using cultured cells [33]. However, low serum media have been shown to maintain the quiescent phenotype of VICs from human aortic valves and prevent EndoMT in VECs from canine mitral valves [33, 35, 36].

The aims of this study, therefore, were to examine cell culture models of MMVD using canine-derived qVICs, aVICs and VECs from normal and diseased mitral valves, under low-serum conditions, identify the appropriate TGFβ factors necessary for disease phenotype induction, and examine the effects of neutralizing TGFβ antibodies, the TGFβ receptor inhibitor SB431542 and the 5HTR2B antagonist LY272015 on cell phenotype, using a combination of morphological assessment, protein immunoblotting, immunohistochemistry, PCR and transcriptomic profiling.

## Materials & methods

### Cell isolation and culture

Cells were collected from healthy young adult dogs (n = 6) and diseased middle-aged dogs (n = 7) of various breeds. The ubiquity of MMVD in the dog makes it problematic to identify age-matched normal controls. Valves were collected with full owner consent, no dogs were euthanized for the purpose of this study. The Royal (Dick) School of Veterinary Studies, Veterinary Ethics Research Committee (Institutional Care and Use Committee) approved this study). Resected valves were graded normal (grade 0) or diseased (grade 1–4) using the standard Whitney classification and graded independently by two observers [37]. VECs were isolated using collagenase digestion as previously reported [33]. Cells were cultivated using standard tissue culture techniques and not used beyond passage 8. A low serum (2% v/v FBS), with added FGF-2 (10ng/ml) and insulin (50ng/ml) DMEM medium (DMEM Low FBS (DLF); Gibco) was used for VICs, and the effect initially compared with a high serum (10% v/v FBS) DMEM medium without FGF-2 and insulin (DMEM High FBS (DH)) [33, 35]. For VECs, canine endothelial cell growth medium (CEM) was used (Cell Applications Inc.). VECs from diseased valves proved difficult to isolate and grow in sufficient numbers and were not included in this study. For TGFβ1, 2 or 3 (Gibco Life Sciences) and the TGFβRI antagonist SB431542, control vehicle was 4mM HCl, 0.1% w/v bovine serum albumin (BSA, Sigma-Aldrich) and diemethylsulfoxide (DMSO) respectively. Pan TGFβ1, 2, 3 and TGFβ1, TGFβ2 and TGFβ3 neutralizing antibodies (R&D Systems) were used at 1 or 10μg/ml. Six-well plates were seeded at 1.5x10^5^ cells/well. 96 well plates used for CellTitre-Glo luminescent cell viability assays (Promega) were seeded at 3x10^3^ cells/well, and at 4x10^5^ for transcriptomic profiling. Harvested cell pellets had RNA and protein extracted as outlined below and supernatants were stored for proteoglycan assays and ELISAs. All experiments were carried out with three technical replicates.

The effects of TGFβ1, 2 or 3 (0.1-10ng/ml) and SB431542 (10μM; Sigma Aldrich,) on VECs and qVICs and aVICs, and the effects of 5HT (100nM), neutralising anti-TGFβ antibodies (1μM) and LY272015 (1μM and 100nM) (Santa Cruz Biotechnology, USA) on qVICs and aVICs, were assessed and details of marker antibodies used are shown in S1 Table.

### Molecular biology analysis

RNA was isolated using the RNeasy mini kit (Qiagen) with cell homogenization using QIAshredders and DNAse I treatment. Quantity and quality of RNA was assessed by NanoDrop ND1000 (Thermo Scientific). For TGFβ RT-PCR experiments reverse transcription with oligo-dT primers was performed on 2μg of total RNA using Omniscript reverse transcriptase (Qiagen) at 37°C for 1h, before PCR with primers of interest using HotStarTaq DNA polymerase (Qiagen). Products were separated on 2% agarose gel and PCR product band intensities were quantified using GelDoc XR (BioRad).

For 5HT experiments cDNA synthesis was performed with Superscript III kit (Invitrogen). The Takyon 2X low Rox SYBR green mastermix dTTP blue (Eurogentec) was used for quantitative PCR (qPCR). qPCR was performed on the MxPro MX3000p Stratagene (Agilent Technologies). Results were analysed using MxPro version 4.1 and the relative expression calculated using ΔΔCt method with normalisation to the geomean of the reference genes MRPS25, GAPDH and RPL32. Primers were designed using Primer 3 v.0.4.0 (http://bioinfo.ut.ee/primer3-0.4.0/) and nucleotide sequences obtained from the NCBI Gene Bank (https://www.ncbi.nlm.nih.gov/genbank/) or Ensembl databases (http://www.ensembl.org) as previously reported (Table 1) (Liu *et al*, 2015). For microarray experiments (n = 3) qVICs were treated with 5ng/mL TGFβ1 and DMSO (Gibco Life Sciences, PHG9204) for 72 hours. aVICs were treated with 10μM SB431542 and 4mM HCL, 0.1% (w/v) BSA (Sigma-Aldrich, S4317) for 96 hours. The Affymetrix GeneChip Canine Gene 1.1 ST Array plate was used for transcriptomic profiling. Affymetrix transcriptome analysis console (version 3.1.0.5) was used to perform paired or unpaired one-way analysis of variance (ANOVA). Differentially expressed gene lists were created using the following criteria: P-value <0.05, log 2 signal intensity >3.5 and fold change of >1.5 or <-1.5. Gene enrichment analysis used Database for Annotation, Visualisation and Integrated Discovery (DAVID; http://www.david.ncifcrf.gov) and Ingenuity Pathway Analysis (IPA; Qiagen, Germany). DAVID predicts the biological processes that are associated with the genes in the list using the gene ontology (GO) terms. Gene lists deriving from differential expression analysis were uploaded to DAVID for analysis, using *Canis familiaris* as the background. IPA identified canonical pathways, upstream regulators, disease and biological functions. Gene list comparisons used the INDEX function of Microsoft Excel.

**Table 1:** Primer sequences used for RT-PCR (TGFβ) and qPCR (5HT).

Gene of Interest	Primer sequence	PCR Product
	**TGFβ experiments**	
*ACTA2* (αSMA)	FP 5’GGGGATGGGACAAAAGGACA 3’ RP 5’GCCACGTAGCAGAGCTTCTCCTTGA3’	525bp
*TAGLN* (SM22)	FP 5’AAGAACGGCGTGATTCTGAG3’ RP 5’CGGTAGTGCCCATCATTCTT3’	269bp
*MYH10* (SMemb)	FP 5’AGAAGCGAGCTGGAAAACTG3’ RP 5’TCTTGCTCTGTCCGATTCTG3’	252bp
*VIM* (Vimentin)	FP 5’GGAGCAGCAGAACAAGATCC3’ RP 5’AGACGTGCCAAAGAAGCATT3’	282bp
*GAPDH*	FP 5’CATCAACGGGAAGTCCATCT3’ RP 5’GTGGAAGCAGGGATGATGTT3’	428bp
	**5HT experiments**	
*ACTA2* (αSMA)	FP 5’CGGCTACTCCTTTGTGACG3’ RP 5'CGTGGCCATCTCGTTCTC3'	100bp
*TAGLN* (SM22)	FP 5’GACATGTTCCAGACCGTCGA3’ RP 5’CAATGACGTGCTTTCCCTCC3’	199bp
*MYH10* (SMemb)	FP 5’AGAAGCGAGCTGGAAAACTG3’ RP 5’TCTTGCTCTGTCCGATTCTG3’	252bp
*GAPDH*	FP 5'GGGAAGATGTGGCGTGAC3' RP 5'GAAGGCCATGCCAGTGAG3'	123bp

### Protein immunoblotting

For protein immunoblotting (Western blotting, WB) cells were lysed in buffer (7M urea, 0.1M DTT, 0.05% (v/v) Triton X-100, 25mM NaCl, 20mM HEPES, pH 7.6) and protein quantified using the Quick Start Bradford assay (Bio-Rad). 30μg of protein lysates were electrophoresed and blotted onto Hybond ECL nitrocellulose membranes (GE Healthcare) and subjected to standard immunoprotocols with primary and secondary antibodies. Bands were visualized by chemiluminescence (ECL Western Blotting Detection Reagents, GE Healthcare, Cat No. RPN2209) using autoradiography film (GE Healthcare), and quantified (ImageJ) (NIH) as a percentage compared to β-actin.

Cells fixed in 4% (v/v) paraformaldehyde and permeabilized with 0.3% (v/v) Triton X-100 were stained using the antibodies listed in Table 1, with DAPI as nuclear stain (Life Technologies). Cell morphology was qualitatively assessed by light and fluorescent microscopy and images captured using a Zeiss Axiovert 40 CFL inverted microscope linked to AxioVision software.

### Enzyme-Linked Imunosorbent Assay (ELISA)

Culture supernatants (at 72hrs) were examined using human TGFβ1 (100% homology), mouse/rat/canine/porcine TGFβ2 (Quantikine ELISA), human TGFβ3 (98% homology), canine tumour necrosis factor (TNF) and recombinant canine IFNγ (DuoSet ELISA, R&D Systems, U.S.A.). TGFβ1 was also assayed at 120hrs. Microplates were read at 450nm using a Perkin Elmer Victor plate-reader. Proteoglycans were detected using a sulphated glycosaminoglycan quantification kit (AMS Biotechnology Ltd) and readings were taken at 515nm. For these experiments values were normalised to total cellular protein (Quick Start Bradford protein assay; Bio-Rad). Values from WB analyses, proteoglycan assays, ELISAs and gene expression were analysed using Student’s t-test.

## Results

**Fig 1:**
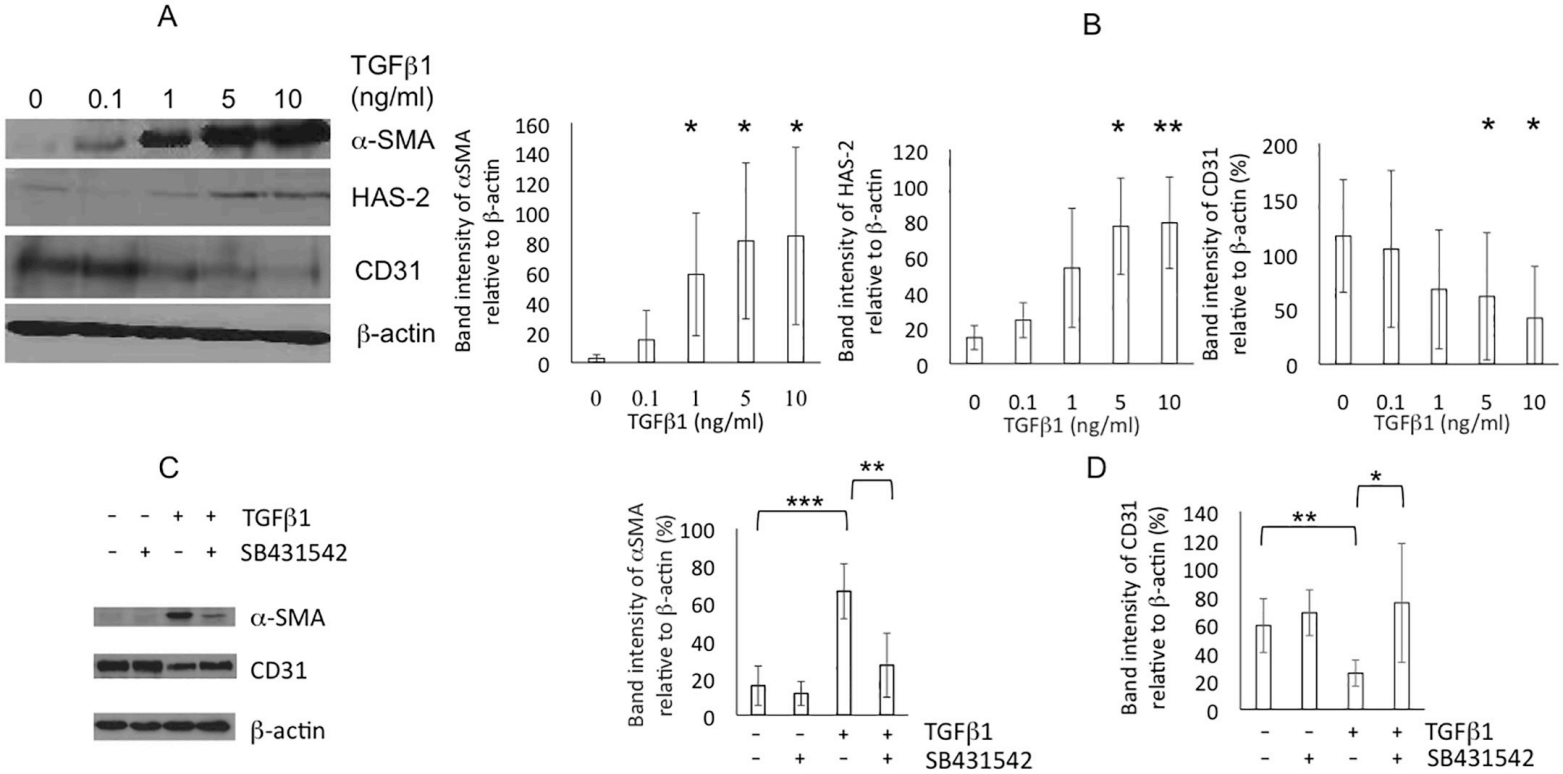
Normal valve endothelial cells (VEC). (A) Representative protein-immunoblot of normal VECs treated with 0, 0.1, 1, 5 and 10ng/ml TGFβ1 for 5 days (n = 4) illustrating expression of αSMA, HAS-2 and CD31. (B) Quantified expression of αSMA in (A) relative to β-actin. (C) Representative protein immunoblot illustrating effects of SB431542 on αSMA and CD31 expression. (D) Quantified expression of αSMA in (C) relative to β-actin. Statistical analysis was performed with Student’s t-test. *, **, *** P<0.05, 0.01, 0.001 respectively.

Initially, the effects of TGFβ1 on canine VEC phenotype were assessed. VECs (n = 4) were grown in CEM and were morphologically normal (cobblestone monolayers with contact inhibition) and αSMA-, unlike those grown in conventional high serum DMEM medium, which underwent EndoMT and transformed into activated myofibroblasts (αSMA+/CD31-). TGFβ1-treated normal VECs grown in CEM also transformed into myofibroblast-like cells with larger central bodies and failed to form compact and confluent cobblestone monolayers. There was a dose-dependent significant (P<0.05) up-regulation of αSMA and hyaluronic acid synthase-2 (HAS-2), and down-regulation of CD31 protein, indicative of EndoMT (Fig 1A and 1B). SB431542 (n = 3) significantly inhibited (P<0.05) TGFβ1-induced EndoMT (Fig 1C and 1D). TGFβ2 and 3 had similar effects over the same dose range.

**Fig 2:**
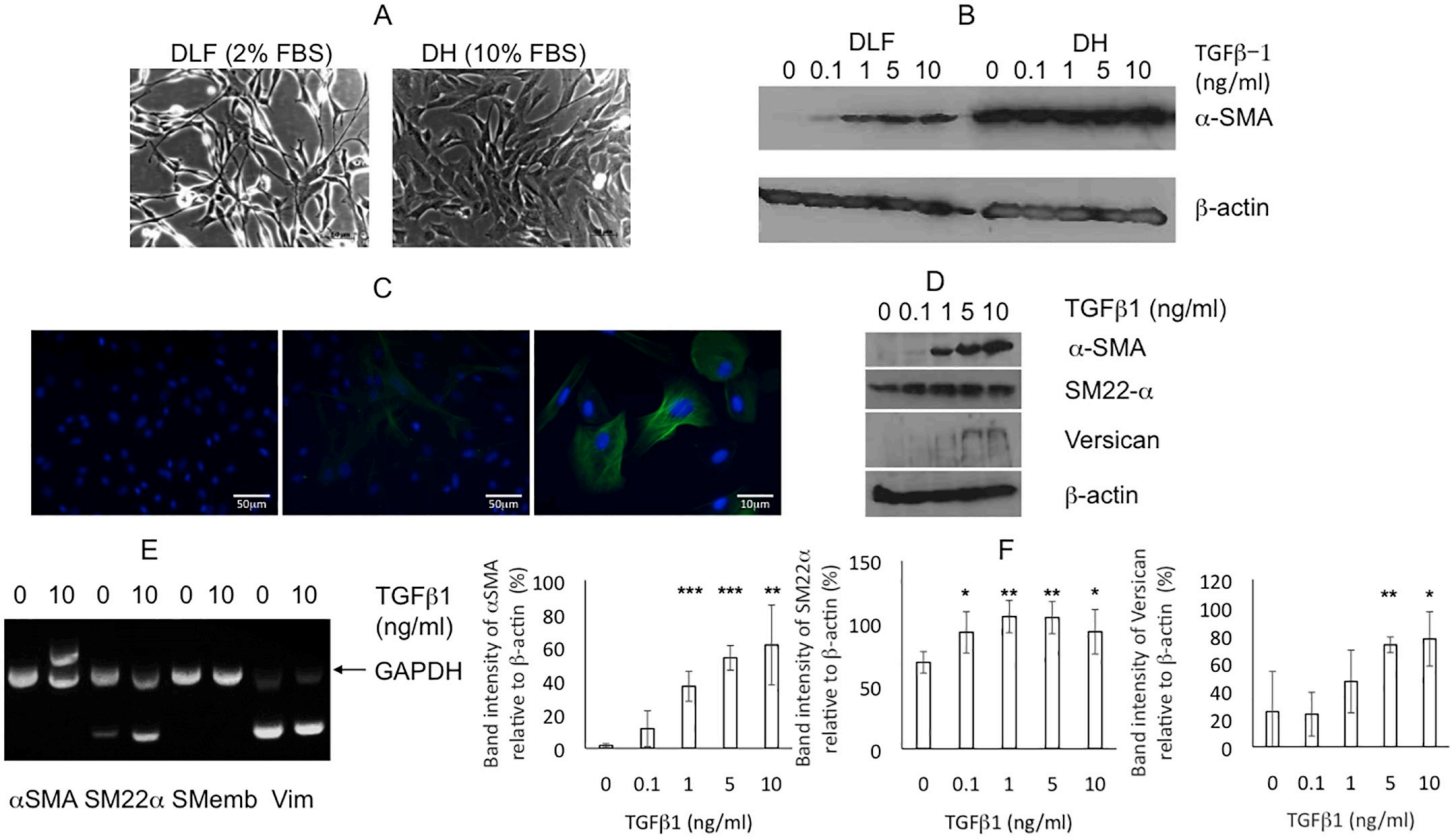
Quiescent valve interstitial cells (VIC) activated by TGFβ1. (A) Representative photomicrographs comparing morphology of normal VICs cultured in 2% (v/v) serum DLF and 10% (v/v) serum DH. Scale bar = 50μM. (B) Comparison of the expression of αSMA in low (DLF) and high serum (DH) conditions and in the presence of 0.1, 1, 5 or 10ng/ml TGFβ1. (C) Representative immunofluorescent photomicrographs of increased αSMA (green) expression in normal VICs in DLF (x20 magnification control left panel) treated with 10ng/ml TGFβ (x20 and x40 magnification centre and right panel respectively); blue DAPI staining showing cell nuclei. (D) Protein immunoblot for αSMA, SM22α and versican in DLF. (E) Representative RT-PCR for αSMA, SM22α, SMemb and vimentin (Vim) (gene specific primers multiplexed PCR with GAPDH housekeeping gene primers) in normal VICs untreated (0ng/ml) or treated (10ng/ml) with TGFβ1 in DLF. (F) Quantification of staining intensities relative to β-actin for αSMA, SM22α and versican respectively in normal VICs (n = 4) in DLF. Statistical analysis was performed with Student’s t-test. *, **, *** P<0.05, 0.01, 0.001 respectively.

We then investigated the effects of DLF and DH media (low and high serum respectively) on canine VIC phenotype. DLF media was superior to DH media in preventing myofibroblast activation and maintaining VIC quiescence. DLF qVICs were less adherent, more elongated and spindle shaped, had smaller central bodies (Fig 2A) and markedly less αSMA expression (Fig 2B). Cells formed into proliferative clusters at 0.1ng/ml TGFβ1 and differentiated into non-proliferative large myofibroblasts at higher concentrations (e.g. 10ng/ml), with fewer viable cells observed.

Since DLF media maintained the quiescent state in normal VICs, we assessed whether TGFβ1 treatment of these cells could induce EndoMT. TGFβ1-treated (n = 4) qVICs grown in DLF medium differentiated into aVICs with significantly increased (P<0.05) expression of αSMA and SM22-α (Fig 2C–2E), and treatment with TGFβ 2 and 3 had the same effect. TGFβ2 and TGFβ3 induced secretion of TGFβ1 by qVICs, while TGFβ1 increased secretion of TGFβ2. On the basis of these results all subsequent VIC experiments were conducted using DLF medium only.

**Fig 3:**
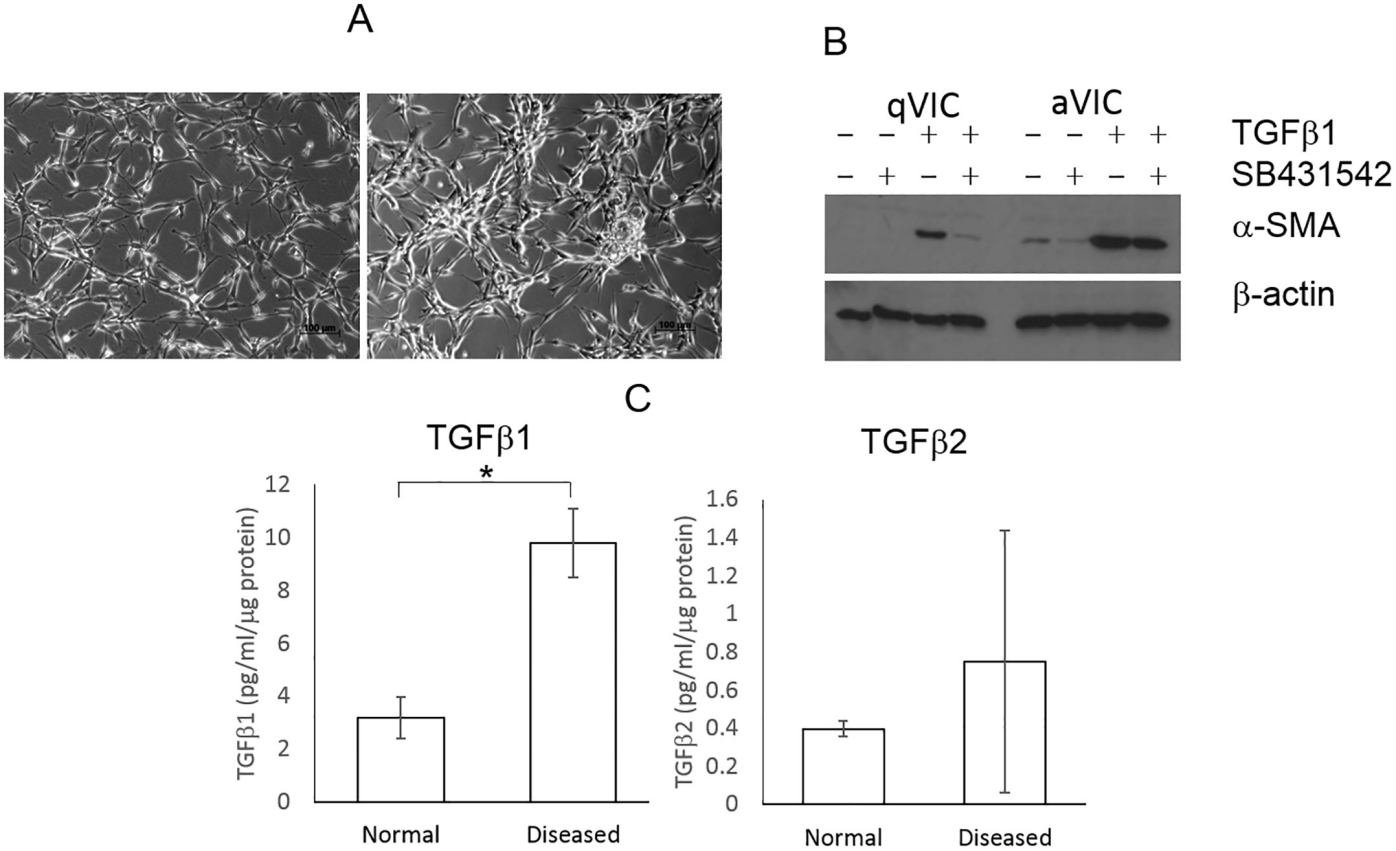
Comparison of qVICs and aVICs. (A) Representative photomicrographs demonstrating morphology of normal (left image) and diseased (right image) VICs. Scale bar = 100μM. (B) Representative protein immunoblotting illustrating αSMA expression in qVICs and aVICs cell cultures, and in response TGFβ1 (5ng/ml) and/or SB431542 (10μM). (C) Mean cumulative TGFβ1 and 2 secretion after 120 hours for qVICs and aVICs (ELISA) (n = 3). Statistical analysis was performed with Student’s t-test. * P<0.05.

The phenotype of aVICs cultured from diseased valves was compared to qVICs from normal valves. aVICs had morphological features and increased αSMA typical of activated myofibroblasts (Fig 3A and 3B). They also had significantly heightened gene expression for *ACTA2* (+40.7 fold change), *TAGLN* (+4.3) and *MYH10* (+4) compared to qVICs. Baseline TGFβ1 secretion (n = 3) was higher in aVICs compared to qVICs, but the difference achieved significance (P<0.05) only after 120hrs in culture (Fig 3C). There was no difference in TGFβ2 and 3 or TNF expression, and all were expressed at low levels. IFNγ was below the detection level of the assay in both qVICs and aVICs. Addition of SB431542 reduced TGFβ1 secretion in both qVICs and aVICS, whether in the presence or absence of exogenous TGFβ1. The addition of TGFβ1 markedly up-regulated expression of αSMA and this was reversed by SB431542.

**Fig 4:**
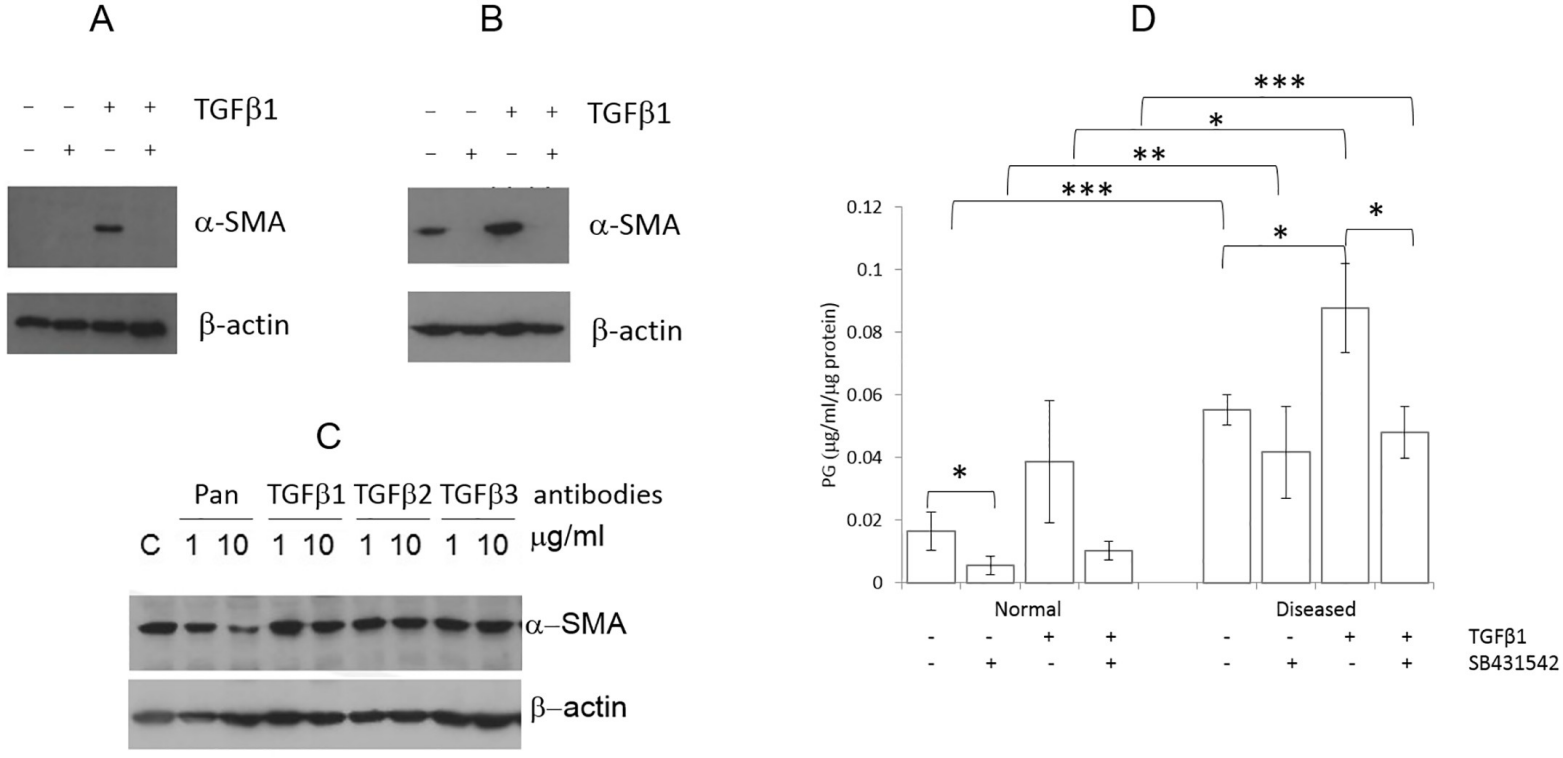
Pharmacological manipulation of αSMA expression. Protein immunoblotting illustrating: (A) Changes in αSMA expression in normal cells treated with vehicle, 5ng/ml of TGFβ1 and 10μM of SB431542 (B) Abolition of αSMA expression in diseased cells by SB431542. (C) Reduction in αSMA expression in diseased cells by treating with a pan anti-TGFβ antibody at 10μg/ml, but not by TGFβ specific antibodies. (D) Changes in deposition of proteoglycans (PG) in normal and diseased cells in response to TGFβ1 and SB4341542. Statistical analysis was performed with Student’s t-test. *, **, and *** P<0.5, 0.1 and 0.01 respectively.

The effects of Inhibition of TGFβ1 signalling on VIC phenotype were then assessed. SB431542 (10μM) prevented or reversed TGFβ1-induced myofibroblast activation in both qVICs treated with TGFβ1 (n = 4) and aVICs (n = 4) (Fig 4A and 4B). The same effect with SB431542 was noted in aVICs that were not treated with TGFβ1. The pan-TGFβ antibody (n = 3), but not specific neutralizing TGFβ antibodies, reduced αSMA expression (Fig 4C) and PG deposition by aVICs, although all neutralizing antibodies could inhibit PG secretion. TGFβ2 and TGFβ3 induced secretion of TGFβ1 by VICs, while TGFβ1 upregulated TGFβ2. TGFβ3 levels were below the range of detection. There was significantly greater (P<0.05) deposition of PG by aVICs compared to qVICs under all conditions (Fig 4D). TGFβ1 increased PG expression in both qVICs and aVICs (n = 3), achieving significance in aVICs, and was inhibited by SB431542.

5HT, alongside TGFβ1, has been reported as a possible factor responsible for the development for MMVD, and we therefore tested the impact of 5HT in our culture system. 5HT-treatment of qVICs (n = 3) had no significant effect on expression of *ACTA2* (αSMA), *5HTR2B*, *TAGLN* or *MYH10* mRNA and there were no discernible changes in cell morphology or PG synthesis. LY272015 treatment (n = 3) of diseased cells at both 100nM and 1μM did not reduce expression of *ACTA2*, *5HTR2B*, *TAGLN* or *MYH10* or PG secretion, but unexpectedly both doses significantly increased expression of *5HTR2B*, and 1μM also increased expression of *TAGLN*. There was no effect of LY272015 on the same gene expression profile in normal cells.

**Fig 5:**
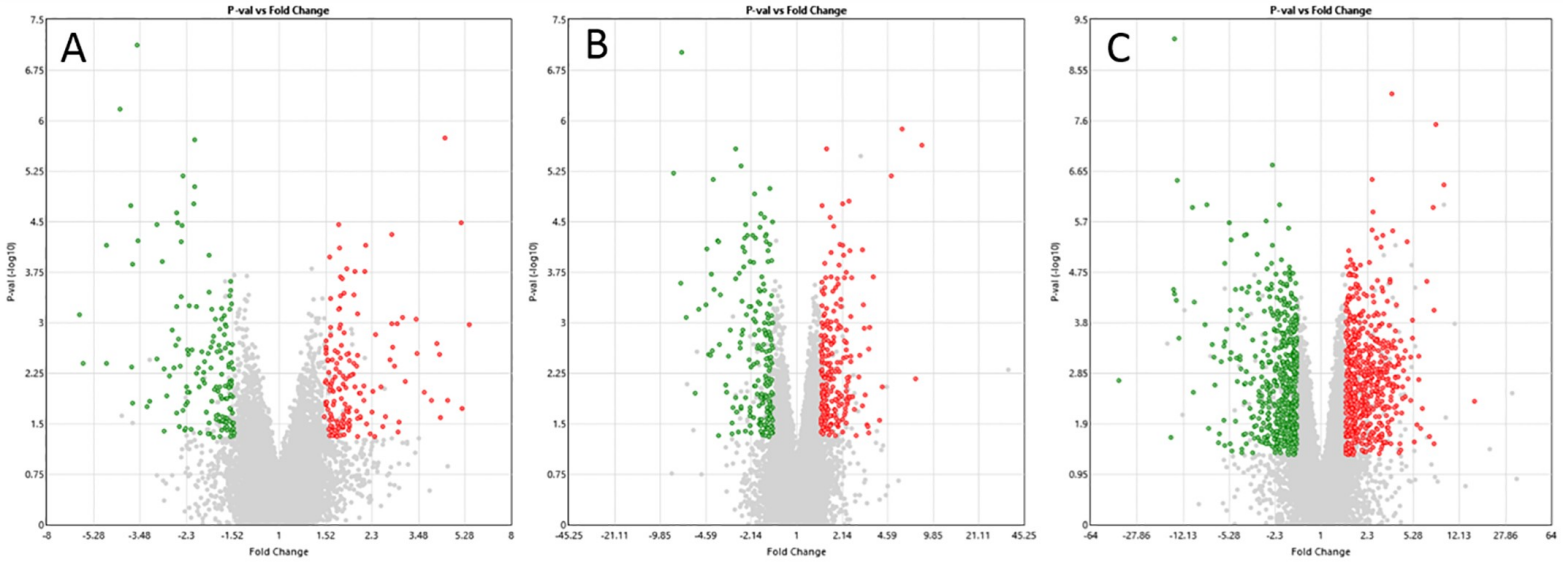
Volcano plots comparing qVICs to aVICs and the effect of treatments on VICs. Red dots represent up-regulated genes, green represent genes that were down-regulated and uncoloured are genes which do not pass the differential expression criteria. The X-axis shows fold change value and the Y-axis shows the P-value. (A) Genes differentially expressed between qVICs + vehicle and qVICs treated with TGFβ1. (B) Genes differentially expressed between aVICs + vehicle and aVICs treated with SB431542. (C) Differentially expressed genes between qVICs and aVICs. Statistical analysis was performed with paired or unpaired ANOVA.

Transcriptomic profiling of qVICs treated with TGFβ1 and aVICs treated with SB431542 was performed to assess the genome-wide consequences of treatment. RT-qPCR was used to validate the microarray and results were generally in agreement except for 5*HTR2B* and *BMPER* in the aVICs/qVICs comparison (S2 Table). Principal component analysis (PCA) of the transcriptomic profiles identified TGFβ1 treated qVICs and untreated aVICs clustering the furthest apart from all other samples. aVICs treated with SB431542 shifted towards the qVIC cluster. Differential gene expression analyses comparing qVICs, qVICs treated with TGFβ1, aVICs, and aVICs treated with SB431542 are shown as volcano plots in Fig 5. Differentially expressed genes (DEGs) were identified by comparing different data sets as follows; qVICs vs aVICs, 902 DEGs (406 down-regulated and 496 up-regulated); TGFβ1-treated qVICs vs vehicle treated qVICs, 275 DEGs (144 down, 131 up); SB431542-treated aVICs vs vehicle treated aVICs, 236 DEGs (115 down 121 up); TGFβ1-treated qVICs vs aVICs, 832 DEGs (490 down, 342up). 102 genes were increased in both TGFβ1-treated qVICs (induced-disease) and aVICs (natural-disease) compared to un-treated qVICs, with only one (*MAMLD1*) having the opposite fold change, and the remaining 101 all up-regulated in both disease groups. Treatment of aVICs with SB431542 reduced the gene signal intensity for *TGFB1*, *2* and *3*, and this was significant for *TGFB3*. TGFβ1 treatment of qVICs increased the expression of *TGFB1*, but not *TGFB2* or *TGFB3*. The full gene lists for these comparisons are shown in S3 to S7 Tables.

To interpret the transcriptomic data further, gene ontology (GO) terms and ingenuity pathway analysis (IPA) was performed. These give insight into the roles that the differentially expressed genes may be involved in. The top 10 GO terms for up- and down-regulated genes for qVICs compared to aVICs were dominated by DNA replication, for qVICs treated with TGFβ1 by extra cellular matrix regulation, for aVICs treated with SB431542 by mesenchymal related terms, for TGFβ-treated qVICs compared to aVICs cell cycle and cell growth, and lastly for the shared gene set for qVICs treated with TGFβ1 and aVICs by extracellular space and smooth muscle proliferation. All the GO term comparisons are shown in S8 Table. IPA identified a number of canonical pathways significantly changed (p<0.05) in the different analyses and the comparisons are listed as follows; 32, qVICs vs aVICs; 29, TGFβ1-qVICs vs qVICs; 37, SB431542-aVICs vs aVICs; 25 TGFβ-treated qVICs vs aVICs; 44, TGFβ1-qVICs/aVICs vs qVICs. The top three pathways for the five data sets are shown in Table 2.

**Table 2:** Canonical pathways.

Analysis	Canonical Pathway	Up	Down	Gene changes in pathway	P-value
qVICs vs aVICs	Cell cycle control of chromosomal replication	15	0	15/49	1.7E-09
	Role of BRCA1 in DNA damage response	15	0	15/73	5.9E-07
	Mitotic roles of polo-like kinase	12	1	12/60	1.7E-06
TGFβ1 treated qVICs vs qVICs	Hepatic fibrosis/Hepatic stellate cell activation	6	4	10/160	0.00076
	Integrin signaling	3	8	11/196	0.00102
	Role of tissue factor in cancer	2	6	8/112	0.00107
SB431542 treated aVICs vs aVICs	Superpathway of cholesterol biosynthesis	10	0	10/22	1E-12
	Cholesterol biosynthesis I	0	7	7/11	13E-10
	Cholesterol biosynthesis II	0	7	7/11	13E-10
TGFβ-treated qVICs vs aVICs	Cell Cycle Control of Chromosomal Replication	19	0	19/49	1.58E-13
	Mitotic Roles of Polo-Like Kinase	16	0	16/60	2.92E-08
	Role of BRCA1 in DNA Damage Response	17	0	17/73	2.29E-08
Genes shared TGFβ1 treated qVICs and aVICs vs qVICs	Superpathway of cholesterol biosynthesis	3	0	3/28	0.0002
	Zymosterol biosynthesis	2	0	2/6	0.000025
	Axonal guidance signaling	4	4	8/457	0.00059

Summary of top three canonical pathways associated with each dataset. The number of genes that were up- and down-regulated in the treated or activated cells in each pathway as well as the total number of genes changed in each pathway is given. The P-value score gives the association of the gene list to the pathway.

Up-stream analysis identified an association of between 837 and 1034 up-stream regulators for these comparisons. In both treatment data sets the top predicted up-stream regulator, not surprisingly, was TGFβ1. The top up-stream regulators in untreated aVICs compared to qVICs included proteins involved in cell cycle control and apoptosis (encoded by *E2FA*, *CDKN1A*, *TP53*). Schematised illustrations of the networks are shown in S1 Fig. CDKN1A and E2FA were also identified as the top two up-stream regulators when comparing TGFβ-treated with qVICs and aVICs. Although E2FA was predicted as the top up-stream regulator, no predications could be made as to its effect on the data set. Overall there was inhibition of the CDKN1A pathway. The shared up-stream regulators for aVICs and TGFβ1-treated qVICs included TGFβ1 and TNF, with the former showing activation and the later slight inhibition. Although transcripts for *TNF* and *IFNG* were identified in all data sets, no significant differences were found and signal intensity for the *TGFB*s was much higher (>2 orders of magnitude).

Network analysis identified the prominent disease and function annotations for each of the four comparison datasets and the top five are shown in S9 Table. Of particular note were functions associated with cell cycle and movement, cardiovascular disease and developmental disorders. Schematic representation of *Cellular movement*, *cardiac arrhythmia and cardiovascular disease; Cardiovascular disease*, *hereditary disorder*, *organismal injury and abnormalities; Cell cycle*, *Cellular movement and cancer; Cell morphology*, *Cardiovascular disease and development disorder* are shown in S2 Fig.

## Discussion

Phenotypic alteration of mitral valve VICs to activated myofibroblasts and EndoMT of VECs are hallmarks of both canine and human myxomatous mitral valve disease [11, 22, 38, 39]. In this study, we have demonstrated for the first time that retention of normal phenotype for canine VICs (*α*SMA-) cultured from unaffected valves is possible using 2% FBS (DLF media), as reported for human aortic valve VICs. These VICs did not undergo spontaneous activation as in previous reports. Furthermore, aVICs from diseased valves under the DLF culture conditions retained their activated myofibroblast phenotype (αSMA+). TGFβ1 could transform qVICs to the disease aVIC phenotype, and this could be reversed using pan-anti-TGFβ antibodies and the kinase inhibitor SB431542. The same effect was found in cells from diseased valves, but 5HT or the 5HT2RB antagonist LY272015 had no effect. While exogenous TGFβ2 and 3 had similar effects as TGFβ1, the low level of secretion of TGFβ2 and 3 suggests that TGFβ1 is the physiologically important isotype. We have also identified the transcriptomic differences between normal and disease cells in culture and the effect of TGFβ1 and SB431542 on gene expression. This is the first time that an effective reversal of induced- and naturally-occurring disease phenotype has been achieved for MMVD, albeit under cell culture conditions, and has implications for understanding pathogenesis and discovery of novel therapeutic options applicable to MMVD.

Several molecular pathways have been identified as likely contributing to MMVD, the most prominent being the TGFβ, 5HT and angiotensin pathways [14, 21, 30, 40, 41]. We examined the effects of the latter two in our low serum culture model for VICs, but the effects were trivial and we therefore decided to concentrate on TGFβ effects, with some additional studies of 5HT in VICs. There are conflicting published data as to which of the TGFβ factors are most important in human and canine MMVD, with some suggesting a single TGFβ factor or a combination of two [19, 21, 24, 42–44]. Determining if one or more TGFβ factors are implicated in canine MMVD, and which one is then the major player, is necessary for selecting an inducer of disease in any cell culture model. In this study, TGFβ1 was identified and selected as the inducer of disease *in vitro* as it was the most abundant TGFβ secreted by cultured VICs and was markedly increased in all diseased cells. We were able to induce some biological features of MMVD in primary culture as early as 3 days. TGFβ1 induced dose-dependent activation of VICs with up-regulation of αSMA, and these VICs had a myofibroblast aVIC morphology. TGFβ1 stimulated VICs to increase proteoglycan deposition. In untreated VICs there were a few αSMA positive cells whereas numbers rose in TGFβ1-treated cultures, which closely resembles the situation in affected native valves [20, 21]. TGFβ1 also induced VEC EndoMT with the endothelial cells differentiating into activated myofibroblasts. Addition of SB431542 or a pan-anti-TGFβ neutralising antibody was able to prevent or reverse myofibroblast differentiation and EndoMT. TGFβ3 was barely detectable above baseline levels and can be excluded from playing an important role in activation of VICs. These data suggest TGFβ1 as the main driver of MMVD, with TGFβ2 possibly playing a secondary role. Overall, the ability to de-differentiate aVICs from diseased valves to a qVIC phenotype and prevent further activation by TGFβ1 is a novel and interesting finding.

In our culture system, both 5HT and LY272015 had no effect on normal and diseased VIC phenotype. The up-regulation of the *5HTR2B* and *TAGLN* by LY272015 is a recognised effect of 5HT antagonists (inverse agonist) [45]. The potential role of 5HT in mitral valvulopathies has been extensively reported, but a causative link has not been shown, apart from the rare instances seen with carcinoid syndrome, appetite suppressants or anti-parkinsonian drugs [30]. Gene profiling has identified up-regulation of 5HT receptor expression in both canine and human mitral valvulopathies, but this appears to be an end-stage disease outcome [14, 16, 19, 31]. As shown here, TGFβ1 can induce *5HT2RB* gene expression in qVICs, and *5HT2RB* is differentially expressed in diseased cells and expression was abolished by SB431542 in our culture system. Data presented here suggests 5HT has either no or only a minor role in VIC phenotype, and *5HT2RB* receptor expression itself is controlled by TGFβ1 in cultured VICs [18, 31].

Proteoglycan deposition was shown to be affected by TGFβ1 in qVICs and inhibited by SB431542 in aVICs, but there was no significant up-regulation of PG deposition in normal VICs with exogenous TGFβ1. This might be due to absence of the modulatory effects of VECs on ECM synthesis [5, 36].

The ability to inhibit and reverse EndoMT in normal cell cultures, or VIC activation in normal and diseased cell culture systems, indicates the potential utility of these cell culture systems as early stage platforms for drug testing or discovery. The TGFβ pathway is a promising target for therapeutic intervention if a system of delivery can be directed specifically to the mitral valves and to those targets that are MMVD exclusive. Further work is needed to examine the down-steam signals and pathways that are TGFβ-dependent and contribute to the ECM changes seen with MMVD. Our previous and ongoing transcriptomic profiling has also identified other pathways of potential interest, but TGFβ1 is still the prime up-stream regulator of disease pathology, irrespective of stage of disease and severity score [14].

Expression of TNF and IFNγ, both at gene and protein level, was low to undetectable (by ELISA) in the VIC populations. This indicates that autocrine VIC signalling of these factors is unlikely. Circulating TNF and IFNγ have both previously been assessed in the dog and likewise do not appear to be significantly altered in disease. However, paracrine signalling from VECs or other potential valvular inflammatory cells cannot be ruled out [46, 47]. The mRNA expression intensity for TGFβ2 and 3 approached that seen with TGFβ1, despite trivial protein expression for both. This likely reflects differences in post-translational effects prior to secretion bound to latent TGFβ binding proteins (LTBPs) [16]. Tissue immunohistochemistry has identified high levels of expression of TGFβ1 and 3, but not TGFβ2, with only TGFβ1 found in the extra-cellular space, in canine MMVD valves [21]. The significantly higher level of TGFβ1 detected in aVIC cultures compared to qVICs indicates its importance in MMVD [21, 23, 29, 48, 49].

Clear transcriptomic differences were identified with TGFβ1-treated qVICs showing the greatest similarity to aVICs, and SB431542-treated aVICs showing similarity to qVICs. These data demonstrate that a credible, though not complete, disease phenotype could be induced, and that the disease phenotype could be transitioned closer to normal phenotype with treatment. Additionally, a shared data set of 102 genes was found comparing induced (TGFβ1-treated qVICs) and natural (aVICs) disease phenotypes. There was also a large un-shared gene list comparing the two “disease” sets. Nevertheless, TGFβ1-treated qVICs did show enhancement of GO terms recognised as hallmarks of MMVD, including extracellular matrix and extracellular space, but also down-regulated positive regulation of ERK1/2, which is not found in the natural disease state, and this may be due to negative feedback consequent on pathway activation. [3, 23, 50, 51]. Treatment with SB431542 altered GO terms and gene expression associated with the aVIC phenotype suggesting signaling through TGFβ RI is in-part responsible for disease phenotype traits [52–54].

IPA identified *Hepatic fibrosis/hepatic stellate cell activation* as the main canonical pathway in TGFβ1-treated qVICs, involving genes typically associated with aVICs in diseased tissue [14]. Up-stream regulator analysis suggested activation of TGFβ1 signaling, with the effects of SB431542 indicating this might involve TGFβ1–3, activin and nodal, but not BMPs [55, 56]. The down-regulation of genes associated with apoptotic pathways was an interesting finding as it fits a narrative for the role of anti-apoptosis in MMVD and other chronic diseases, where there is an increase in number and persistence of activated myofibroblasts, a condition that is TGFβ1-dependent [22, 29, 53, 57]. Network analysis also identified disease and function biological terms, including *cell morphology*, *cardiovascular disease* and *development disorder* that would be relevant to MMVD, where transition of qVICs to aVICs and initiation of EndoMT are cardinal features of disease [3, 11, 12, 57].

Specific gene expression by RT-qPCR identified differential expression comparing qVICs and aVICs, including typical markers of disease *ACTA2*, *TAGLN*, *MYH10* and *HTR2B* [12, 14, 34]. Surprisingly, *MYH10* expression was not affected by TGFβ1 or SB431542, suggesting control by another factor or delayed alteration under culture conditions. *MYH10* encodes embryonic smooth muscle myosin, which is differentially expressed in aVICs from diseased valve tissue, expressed alone or with αSMA [11, 34, 57]. While the potential role of 5HT in MMVD has been previously reported, the regulation of the expression of *HTR2B* by TGFβ1 (up-) and SB431542 (down-) suggests the proposed contribution of 5HT to MMVD pathogenesis is likely to be TGFβ1-dependent [31].

In order to optimise canine mitral valve cell cultures as credible disease model, further work is needed to examine the regulation of signaling pathways, the relationship between the VICs and VECs, the effects of endothelial trauma, mechanical strain and shear stress, and the control of matrix-located latent-bound TGFβs release [34, 58].

## Limitations

While sample size was sufficient to allow significant differences to be identified, larger samples might have given more detailed insight into the changes seen. Sample size also needs to be considered given the problem of cell phenotype heterogeneity when sampling from a naturally occurring diseased population. Confounding factors of age, disease severity, and, in the case of the dog, breed, also need to be considered. However, it can be argued that the data presented here represent a more complete cross-section of the population than if a single breed had been examined, and are therefore more representative of disease phenotype. The difficulty in harvesting and culturing sufficient VECs from diseased valves was disappointing as modelling MMVD will require examination of those cells and how they interact with stromal VICs, and further work is needed in this area. Further work is also needed to examine changes in specific ECM components such as collagens, elastin and proteoglycans in these culture systems. Lastly, while this 2D monoculture does give some insight into the patho-biology of MMVD, more complex 3D single cell and co-cultures would allow more detailed modelling of both the normal and diseased valve.

## Conclusion

We have shown it is feasible to induce, in a TGFβ1 concentration-dependent manner, EndoMT and activated myofibroblast phenotype in normal canine VECs and VICs respectively, and to reverse naturally-occurring disease phenotype in canine mitral valve VICs. We identified TGFβ1 as the main driver of disease phenotype, and that inhibition of TGFβ can completely inhibit VIC activation. We did not identify any significant contribution of 5HT, but our data indicate that any changes in 5HT signalling were likely to be TGFβ1-mediated. Simple cell culture systems, using a low serum protocol, can partially model MMVD and more sophisticated culture methods can now be developed to achieve greater approximation to the gene and protein changes seen in disease.

## Supporting information

S1 Table Details of antibodies used. IF, immunofluorescence and protein; WB Western protein immuno-blotting; VIC, valve interstitial cell; VEC, valve endothelial cell; EndoMT, endothelial-to-mesenchymal transition.
(PDF)

S2 Table RT-qPCR validation of microarray data. A. Microarray data for selected genes. B. RT-qPCR data for the same selected genes. Differences are shown for *5HTR2B* and *BMPER* highlighted in yellow for aVIC/qVIC dataset comparison.
(PDF)

S3 Table Gene list qVICs vs aVICs with fold change < or > 1.5 (902 differentially expressed genes; 406 down 496 up). (PDF)

S4 Table Gene list TGFβ1-treated qVICs vs qVICs with fold change < or > 1.5 (275 differentially expressed genes; 144 down, 131 up). (PDF)

S5 Table Gene list SB431542 treated aVICs vs aVICs with fold change < or > 1.5 (236 differentially expressed genes; 115 down, 121 up). (PDF)

S6 Table Gene list TGFβ1-treated qVICs vs aVICs with fold change < or > 1.5 (832 differentially expressed genes; 490 down, 342 up). (PDF)

S7 Table 102 shared differentially expressed genes in the TGFβ1-treated qVICs and aVICs datasets compared to un-treated qVICs. All gene showed the same direction of change (down) except for *MALD1*.
(PDF)

S8 Table Top 10 up and down regulated GO terms for different data set comparisons. A. qVICs/aVICs; B. TGFβ1-qVICs/qVICs; C. SB431542-aVICs/aVICs; D. TGFβ1-qVICs/aVICs; E. shared gene set for TGFB1-treated qVICs and aVICs.
(PDF)

S9 Table Disease and function annotations. Top five disease and function networks associated with the genes differentially expressed between A TGFβ1-qVICs/qVICs; B SB431542-aVICs/aVICs; C qVICs/aVICs; D TGFβ1-qVICs/aVICs. Underlined networks are shown schematically below.
(PDF)

S1 Fig Gene networks and up-stream regulators (A-E). Networks of genes differentially expressed showing downstream effects from the extracellular space to the nucleus. Genes are coloured red and green to represent up- or down-regulation in the dataset. Dotted lines connecting the regulator to these genes show the expected effect of regulator signalling: orange—activation, blue—inhibition, yellow—result inconsistent and grey—effect not predicted. A. TGFβ1 network; TGFβ1-qVICs/qVICs; B. TGFβ1 network; SB431542-aVICs/aVICs; C. E2F4 network; aVICs/qVICs; D. CDKN1A network; aVICs/qVICs; E.TGFβ1 network; aVICs/TGFβ1-qVICs/.
(TIF)

S2 Fig Schematic representations of disease and function networks highlighted in S7 Table. Genes are shown in their protein cellular location with red indicating up-regulation, green down-regulation and un-coloured showing no change in the dataset. A. Cellular movement, cardiac arrhythmia and cardiovascular disease; B. Cardiovascular disease, hereditary disorder, organismal injury and abnormalities; C. Cell cycle, cellular movement and Cancer; D. Cell morphology, cardiovascular disease and development disorder.
(TIF)
