# Supplementary material for: Evaluation of canine 2D cell cultures as models of myxomatous mitral valve degeneration
Source: PLoS One. 2019 Aug 15;14(8):e0221126. doi: 10.1371/journal.pone.0221126 (PMC6695117; doi:10.1371/journal.pone.0221126)
Supplement: S1 Table — IF, immunofluorescence and protein; WB Western protein immuno-blotting; VIC, valve interstitial cell; VEC, valve endothelial cell; EndoMT, endothelial-to-mesenchymal transition. (PDF) [file pone.0221126.s001.pdf]

S1 Table . Details of antibodies used. IF, immunofluorescence and protein; WB Western protein immuno-blotting; VIC, valve interstitial cell; VEC, valve endothelial cell; EndoMT, endothelial-to-mesenchymal transition.

Antibody antigen	Application	Source	Catalogue number	IF dilution	WB dilution	MW (kDa)
αSMA	Marker of activation of VICs, enhanced differentiation and EndoMT	Mouse monoclonal	Sigma-Aldrich A2547	1:200	1:1000	42
SM22-α	Myofibroblast differentiation marker	Rabbit polyclonal	Abcam AB14106	-	1:1000	23
Versican	Proteoglycan	Rabbit polyclonal	Millipore AB1033	-	1:100	1000 (core 400)
CD31	Standard VEC marker	Rabbit polyclonal	Abcam AB28364	-	1:100	130
HAS-2	EndoMT marker	Mouse monoclonal	Abcam AB140671	-	1:500	64
β-actin	Loading control	Mouse monoclonal	Abcam AB6276	-	1:20000	42
Alexafluor 488 anti-mouse	Fluorescent secondary antibody(IF)	Goat IgG (H+L)	Invitrogen A10667	1:100	-	-
HRP anti-mouse	Secondary antibody (WB)	Rabbit	DAKO P0260	-	1:1000	-
HRP anti-rabbit	Secondary antibody (WB)	Swine	DAKO P0217	-	1:3000	-
