# Supplementary material for: Evaluation of canine 2D cell cultures as models of myxomatous mitral valve degeneration
Source: PLoS One. 2019 Aug 15;14(8):e0221126. doi: 10.1371/journal.pone.0221126 (PMC6695117; doi:10.1371/journal.pone.0221126)
Supplement: S2 Table — A. Microarray data for selected genes. B. RT-qPCR data for the same selected genes. Differences are shown for 5HTR2B and BMPER highlighted in yellow for aVIC/qVIC dataset comparison. (PDF) [file pone.0221126.s002.pdf]

S2 Table. RT-qPCR validation of microarray data.

A. Microarray data for selected genes. B. RT-qPCR data for the same selected genes. Differences are shown for *5HTR2B* and *BMPER* highlighted in yellow for aVIC/qVIC dataset comparison.

A. Microarray results

Gene name	qVIC treated with TGFβ1		aVIC treated with SB431542		aVIC compared to qVIC	
	Fold change	P-value	Fold change	P-value	Fold change	P-value
ACTA2	1.95	0.0437	-6.36	0.0184	7.69	0.0051
TAGLN	2.1	0.0228	-2.58	0.034	3.78	0.0088
MYH10	-1.13	0.7948	-1.34	0.5271	2.18	0.0297
HTR2B	3.71	0.0176	-5.16	0.0308	3.35	0.1184
HBEGF	-1.73	0.0209	2.1	0.0003	-1.67	0.0085
BMPER	-3.09	0.0064	1.6	0.4142	-3.98	0.0257
VCAM1	-1.83	0.0004	1.59	0.1007	-2.79	0.0076
PDK4	-5.11	0.0021	-1.78	0.2011	-7.89	0.0009
FAP	4.71	0.0032	-6.94	0.0141	6.73	0.0063
SLC10A6	-1.96	0.0481	-1.17	0.993	-2.14	0.0109

B. RT-qPCR results

Gene name	qVIC treated with TGFβ1		aVIC treated with SB431542		aVIC compared to qVIC	
	Fold change	P-value	Fold change	P-value	Fold change	P-value
ACTA2	6.01	0.03	-22.36	0.021	37.4	0.003
TAGLN	2.84	0.043	-2.72	0.042	3.92	0.028
MYH10	-1.74	0.086	-1.07	0.572	2.9	0.05
HTR2B	2.43	0.043	-9.34	0.042	2.33	0.029
HBEGF	-2.69	0.44	2.95	0.035	-2.5	0.021
BMPER	-5.28	0.008	2.31	0.12	-4.2	0.092
VCAM1	-2.83	0.049	2.78	0.065	-6.93	0.007
PDK4	-6.88	0.005	3.89	0.09	-74.07	0.012
FAP	7.64	0.002	-5.71	0.001	8.54	0.026
SLC10A6	-2.65	0.013	2.07	0.237	-4.53	0.042
