# Supplementary material for: Evaluation of canine 2D cell cultures as models of myxomatous mitral valve degeneration
Source: PLoS One. 2019 Aug 15;14(8):e0221126. doi: 10.1371/journal.pone.0221126 (PMC6695117; doi:10.1371/journal.pone.0221126)
Supplement: S3 Table — (PDF) [file pone.0221126.s003.pdf]

S3 Table. Gene list qVICs vs aVICs with fold change < or > 1.5 (902 differentially expressed genes; 406 down 496 up)

Fold Change	Gene Symbol	Description
-38.85	ACKR4	atypical chemokine receptor 4
-16.06	HCN1	hyperpolarization activated cyclic nucleotide gated potassium channel 1
-14.47	PKHD1L1	polycystic kidney and hepatic disease 1 (autosomal recessive)-like 1
-14.18	HECW1	HECT, C2 and WW domain containing E3 ubiquitin protein ligase 1
-14.07	PKHD1L1	polycystic kidney and hepatic disease 1 (autosomal recessive)-like 1
-13.65	PKHD1L1	polycystic kidney and hepatic disease 1 (autosomal recessive)-like 1
-13.5	EDNRB	endothelin receptor type B
-13.02	LOC479476	arachidonate 12-lipoxygenase, 12S-type
-12.26	GJA5	gap junction protein, alpha 5, 40kDa
-11.84	PKHD1L1	polycystic kidney and hepatic disease 1 (autosomal recessive)-like 1
-10.19	SPINT1	serine peptidase inhibitor, Kunitz type 1
-10.13	BPI	bactericidal/permeability-increasing protein
-9.88	CXCL8	chemokine (C-X-C motif) ligand 8
-8.14	GFRA2	GDNF family receptor alpha 2
-7.89	PDK4	pyruvate dehydrogenase kinase, isozyme 4
-7.21	SERPINI1	serpin peptidase inhibitor, clade I (neuroserpin), member 1
-6.93	ENSCAFG00000031682	Chromosome 12: 20,320,070-20,334,201
-6.84	ADAMTSL2	ADAMTS-like 2
-6.1	MAMDC2	MAM domain containing 2
-5.76	DHRS9	dehydrogenase/reductase (SDR family) member 9
-5.73	FAM216B	family with sequence similarity 216, member B
-5.72	ATF7IP2	activating transcription factor 7 interacting protein 2
-5.72	CASP14	caspase 14, apoptosis-related cysteine peptidase
-5.53	FRMD5	FERM domain containing 5
-5.26	STRIP2	striatin interacting protein 2
-5.18	NPPC	natriuretic peptide C
-5.15	HEPACAM	hepatic and glial cell adhesion molecule
-5.06	LOC487628	thrombospondin type-1 domain-containing protein 4
-4.82	HCN1	hyperpolarization activated cyclic nucleotide gated potassium channel 1

-4.68	DLX2	distal-less homeobox 2
-4.67	HPSE	heparanase
-4.63	CLDN1	claudin 1
-4.58	IGF2BP3	insulin-like growth factor 2 mRNA binding protein 3
-4.45	ZDHHC14	zinc finger, DHHC-type containing 14
-4.41	CHRM2	cholinergic receptor, muscarinic 2
-4.35	SOD3	superoxide dismutase 3, extracellular
-4.32	CXADR	coxsackie virus and adenovirus receptor
-4.29	SELL	selectin L
-4.26	PTGS1	prostaglandin-endoperoxide synthase 1 (prostaglandin G/H synthase and cyclooxygenase)
-4.23	IGF1	insulin-like growth factor 1 (somatomedin C)
-4.08	EMB	embigin
-4.02	PRSS12	protease, serine, 12 (neurotrypsin, motopsin)
-3.98	BMPER	BMP binding endothelial regulator
-3.98	RGS16	regulator of G-protein signaling 16
-3.96	BPHL	biphenyl hydrolase-like (serine hydrolase)
-3.88	MAFB	v-maf avian musculoaponeurotic fibrosarcoma oncogene homolog B
-3.86	ACAN	aggrecan
-3.8	DOCK5	dedicator of cytokinesis 5
-3.71	TMEM100	transmembrane protein 100
-3.62	GYPC	glycophorin C (Gerbich blood group)
-3.58	RRAD	Ras-related associated with diabetes
-3.56	ACE	angiotensin I converting enzyme
-3.5	CP	ceruloplasmin (ferroxidase)
-3.47	SORBS1	sorbin and SH3 domain containing 1
-3.39	LOC100686073	metallothionein-1
-3.36	BICD1	bicaudal D homolog 1 (Drosophila)
-3.29	SEZ6L	seizure related 6 homolog (mouse)-like
-3.29	PTPRE	protein tyrosine phosphatase, receptor type, E
-3.18	KCND3	potassium channel, voltage gated Shal related subfamily D, member 3
-3.12	FAM180A	family with sequence similarity 180, member A
-3.11	CCDC3	coiled-coil domain containing 3
-3.1	TUBB4A	tubulin, beta 4A class IVa
-3.09	RANBP3L	RAN binding protein 3-like
-3.08	OSGIN2	oxidative stress induced growth inhibitor family member 2

-3.06	GRAP2	GRB2-related adaptor protein 2
-2.96	DTNB	dystrobrevin, beta
-2.94	OLFML2A	olfactomedin-like 2A
-2.9	ARL4D	ADP-ribosylation factor-like 4D
-2.89	CDS1	CDP-diacylglycerol synthase (phosphatidate cytidylyltransferase) 1
-2.87	AFF3	AF4/FMR2 family, member 3
-2.87	PTGFRN	prostaglandin F2 receptor inhibitor
-2.87	RAPGEF4	Rap guanine nucleotide exchange factor (GEF) 4
-2.85	GUCY1B3	guanylate cyclase 1, soluble, beta 3
-2.81	MIR1838	microRNA mir-1838
-2.79	VCAM1	vascular cell adhesion molecule 1
-2.77	LOC487977	cell surface glycoprotein CD200 receptor 1
-2.75	PCBD1	pterin-4 alpha-carbinolamine dehydratase/dimerization cofactor of hepatocyte nuclear factor 1 alpha
-2.72	MAP7	microtubule-associated protein 7
-2.69	UCP2	uncoupling protein 2 (mitochondrial, proton carrier)
-2.68	IGSF3	immunoglobulin superfamily, member 3
-2.67	PDCD4	programmed cell death 4 (neoplastic transformation inhibitor)
-2.65	CPXM1	carboxypeptidase X (M14 family), member 1
-2.64	ANO5	anoctamin 5
-2.64	PIPOX	pipecolic acid oxidase
-2.63	DEPDC7	DEP domain containing 7
-2.62	TMTC1	transmembrane and tetratricopeptide repeat containing 1
-2.61	MTHFS	5,10-methenyltetrahydrofolate synthetase (5-formyltetrahydrofolate cyclo-ligase)
-2.6	ZDHHC14	zinc finger, DHHC-type containing 14
-2.6	P2RY1	purinergic receptor P2Y, G-protein coupled, 1
-2.6	DTNA	dystrobrevin, alpha
-2.58	SLC25A42	solute carrier family 25, member 42
-2.57	DNM3	dynamin 3
-2.57	ASIC2	acid sensing (proton gated) ion channel 2
-2.56	HSPA2	heat shock 70kDa protein 2
-2.55	MFAP3L	microfibrillar-associated protein 3-like
-2.53	BAALC	brain and acute leukemia, cytoplasmic
-2.52	TMTC2	transmembrane and tetratricopeptide repeat containing 2

-2.52	SELP	selectin P (granule membrane protein 140kDa, antigen CD62)
-2.5	KIAA1217	KIAA1217 ortholog
-2.47	FGG	fibrinogen gamma chain
-2.46	FNIP2	folliculin interacting protein 2
-2.45	KLF11	Kruppel-like factor 11
-2.45	OTUD1	OTU deubiquitinase 1
-2.45	ARHGAP24	Rho GTPase activating protein 24
-2.42	VOPP1	vesicular, overexpressed in cancer, prosurvival protein 1
-2.42	SLC2A3	solute carrier family 2 (facilitated glucose transporter), member 3
-2.38	SHISA2	shisa family member 2
-2.38	PTHLH	parathyroid hormone-like hormone
-2.38	CXCL16	chemokine (C-X-C motif) ligand 16
-2.36	SLC22A23	solute carrier family 22, member 23
-2.36	DNM3	dynamin 3
-2.35	TAL1	T-cell acute lymphocytic leukemia 1
-2.33	MAP4	microtubule associated protein 4
-2.33	MAP4	microtubule associated protein 4
-2.33	MAP4	microtubule associated protein 4
-2.32	BAMBI	BMP and activin membrane-bound inhibitor
-2.31	CNTN3	contactin 3 (plasmacytoma associated)
-2.31	PIK3CB	phosphatidylinositol-4,5-bisphosphate 3-kinase, catalytic subunit beta
-2.3	FBLN2	fibulin 2
-2.29	YPEL1	yippee-like 1
-2.28	TSGA10	testis specific, 10
-2.26	ADGRF2	adhesion G protein-coupled receptor F2
-2.26	MOSPD2	motile sperm domain containing 2
-2.25	TSPAN13	tetraspanin 13
-2.25	PTPRN2	protein tyrosine phosphatase, receptor type, N polypeptide 2
-2.24	GALNT10	polypeptide N-acetylgalactosaminyltransferase 10
-2.23	GMDS	GDP-mannose 4,6-dehydratase
-2.22	PDZK1IP1	PDZK1 interacting protein 1
-2.22	TGFBR3	transforming growth factor, beta receptor III
-2.21	MSX1	msh homeobox 1
-2.2	EPHA5	EPH receptor A5
-2.17	CTNND2	catenin (cadherin-associated protein), delta 2

-2.17	DPYD	dihydropyrimidine dehydrogenase
-2.16	SALL3	spalt-like transcription factor 3
-2.16	LGMN	legumain
-2.14	SLC10A6	solute carrier family 10 (sodium/bile acid cotransporter), member 6
-2.14	DOK5	docking protein 5
-2.14	IRS1	insulin receptor substrate 1
-2.12	AGBL3	ATP/GTP binding protein-like 3
-2.12	CD58	CD58 molecule
-2.11	MIR22	microRNA mir-22
-2.09	NMRK1	nicotinamide riboside kinase 1
-2.09	GCNT4	glucosaminyl (N-acetyl) transferase 4, core 2
-2.09	SCARB2	scavenger receptor class B, member 2
-2.09	DDAH1	dimethylarginine dimethylaminohydrolase 1
-2.09	LOC608853	GTP:AMP phosphotransferase AK3, mitochondrial pseudogene
-2.08	MIR500	microRNA mir-500
-2.07	ELOVL7	ELOVL fatty acid elongase 7
-2.06	RND1	Rho family GTPase 1
-2.06	MYO1E	myosin IE
-2.06	ITGA2B	integrin, alpha 2b (platelet glycoprotein IIb of IIb/IIIa complex, antigen CD41)
-2.05	PLXNB1	plexin B1
-2.05	RORA	RAR-related orphan receptor A
-2.04	CACNG4	calcium channel, voltage-dependent, gamma subunit 4
-2.04	ENTPD3	ectonucleoside triphosphate diphosphohydrolase 3
-2.04	PLAU	plasminogen activator, urokinase
-2.03	PARK2	parkin RBR E3 ubiquitin protein ligase
-2.03	GABBR1	gamma-aminobutyric acid (GABA) B receptor, 1
-2.02	MGAT3	mannosyl (beta-1,4-)-glycoprotein beta-1,4-N-acetylglucosaminyltransferase
-2.02	BCR	breakpoint cluster region
-2.02	RHOV	ras homolog family member V
-2.01	LYPD6	LY6/PLAUR domain containing 6
-2.01	RRAGD	Ras-related GTP binding D
-2.01	STEAP1	six transmembrane epithelial antigen of the prostate 1
-2	FBXL2	F-box and leucine-rich repeat protein 2
-2	PSTPIP2	proline-serine-threonine phosphatase interacting protein 2

-2	CLCN5	chloride channel, voltage-sensitive 5
-1.99	CD200	CD200 molecule
-1.98	MMP15	matrix metallopeptidase 15 (membrane-inserted)
-1.98	MIR8810	microRNA mir-8810; adenosine monophosphate deaminase 3
-1.97	TRPM6	transient receptor potential cation channel, subfamily M, member 6
-1.97	P2RX2	purinergic receptor P2X, ligand gated ion channel, 2
-1.96	NFKBIA	nuclear factor of kappa light polypeptide gene enhancer in B-cells inhibitor, alpha
-1.96	PPAP2B	phosphatidic acid phosphatase type 2B
-1.96	PARK2	parkin RBR E3 ubiquitin protein ligase
-1.96	ENSCAFG00000030567	Chromosome 2: 36,317,518-36,322,334
-1.96	STK32B	serine/threonine kinase 32B
-1.96	PPAP2B	phosphatidic acid phosphatase type 2B
-1.95	FAM19A4	family with sequence similarity 19 (chemokine (C-C motif)-like), member A4
-1.94	C16H8orf4	chromosome 16 open reading frame, human C8orf4
-1.94	LOC487173	protocadherin beta-6
-1.93	CAPN3	calpain 3
-1.93	FOXN3	forkhead box N3
-1.92	TIAM2	T-cell lymphoma invasion and metastasis 2
-1.92	LOC102152410	GTP:AMP phosphotransferase AK3, mitochondrial pseudogene
-1.91	LOC481722	complement C4-A
-1.91	KTI12	KTI12 chromatin associated homolog
-1.91	MT2A	metallothionein 1H
-1.91	SPRY2	sprouty RTK signaling antagonist 2
-1.91	PPP1R26	protein phosphatase 1, regulatory subunit 26
-1.9	ADAMTS1	ADAM metallopeptidase with thrombospondin type 1 motif, 1
-1.9	RASL10B	RAS-like, family 10, member B
-1.9	LOC481227	neuronal-specific septin-3
-1.9	C15H1orf228	chromosome 15 open reading frame, human C1orf228
-1.89	LOC100687306	olfactory receptor 12-like
-1.88	NDP	Norrie disease (pseudoglioma)
-1.88	MAL	mal, T-cell differentiation protein
-1.88	EPHB2	EPH receptor B2
-1.88	KAT2B	K(lysine) acetyltransferase 2B
-1.88	TSPAN11	tetraspanin 11

-1.88	FLCN	folliculin
-1.88	RENBP	renin binding protein
-1.87	AK3	adenylate kinase 3
-1.87	STK17B	serine/threonine kinase 17b
-1.87	RALGDS	ral guanine nucleotide dissociation stimulator
-1.87	ZRSR2	zinc finger (CCCH type), RNA-binding motif and serine/arginine rich 2
-1.86	CCBL1	cysteine conjugate-beta lyase, cytoplasmic
-1.85	NRP1	neuropilin 1
-1.85	MMP24	matrix metallopeptidase 24 (membrane-inserted)
-1.85	GPR137B	G protein-coupled receptor 137B
-1.84	IL1R1	interleukin 1 receptor, type I
-1.84	GRAMD2	GRAM domain containing 2
-1.83	PLD2	phospholipase D2
-1.82	LGALSL	lectin, galactoside-binding-like
-1.82	LOC474850	heat shock 70 kDa protein 1-like
-1.82	NR4A1	nuclear receptor subfamily 4, group A, member 1
-1.81	ZHX2	zinc fingers and homeoboxes 2
-1.8	PPP1R15A	protein phosphatase 1, regulatory subunit 15A
-1.8	FGD6	FYVE, RhoGEF and PH domain containing 6
-1.8	MTIF3	mitochondrial translational initiation factor 3
-1.8	C5H11orf87	chromosome 5 open reading frame, human C11orf87
-1.79	TMEM2	transmembrane protein 2
-1.79	FOXRED2	FAD-dependent oxidoreductase domain containing 2
-1.78	ISCA1	iron-sulfur cluster assembly 1
-1.78	SLC44A1	solute carrier family 44 (choline transporter), member 1
-1.78	RASGEF1A	RasGEF domain family, member 1A
-1.78	AFF1	AF4/FMR2 family, member 1
-1.78	ZNF521	zinc finger protein 521
-1.77	TULP4	tubby like protein 4
-1.77	FOXD2	forkhead box D2
-1.77	LOC106559978	low-density lipoprotein receptor-related protein 5-like; low density lipoprotein receptor-related protein 5
-1.76	MTURN	maturin, neural progenitor differentiation regulator homolog (Xenopus)
-1.76	TMEM140	transmembrane protein 140

-1.76	RELN	reelin
-1.76	LMNTD1	lamin tail domain containing 1
-1.76	MOCOS	molybdenum cofactor sulfurase
-1.75	CTNNAL1	catenin (cadherin-associated protein), alpha-like 1
-1.75	RILP	Rab interacting lysosomal protein
-1.74	RAB32	RAB32, member RAS oncogene family
-1.74	IGF2R	insulin-like growth factor 2 receptor
-1.74	NEIL1	nei-like DNA glycosylase 1
-1.73	GAB1	GRB2-associated binding protein 1
-1.73	HTATIP2	HIV-1 Tat interactive protein 2, 30kDa
-1.72	NCOA7	nuclear receptor coactivator 7
-1.72	TXNIP	thioredoxin interacting protein
-1.72	EXTL3	exostosin-like glycosyltransferase 3
-1.72	REV3L	REV3 like, DNA directed polymerase zeta catalytic subunit
-1.71	UTP23	UTP23, small subunit (SSU) processome component, homolog (yeast)
-1.71	MAT2A	methionine adenosyltransferase II, alpha
-1.71	BLVRA	biliverdin reductase A
-1.71	CCDC113	coiled-coil domain containing 113
-1.71	ERO1B	endoplasmic reticulum oxidoreductase beta
-1.71	ELFN1	extracellular leucine-rich repeat and fibronectin type III domain containing 1
-1.71	AMDHD2	amidohydrolase domain containing 2
-1.71	DOCK11	dedicator of cytokinesis 11
-1.71	ENSCAFG00000037897	Chromosome 14: 28,759,918-28,762,414
-1.7	VIT	vitrin
-1.69	ZNF568	zinc finger protein 568
-1.69	LOC100856635	intersectin-1; intersectin 1 (SH3 domain protein)
-1.69	PARP8	poly (ADP-ribose) polymerase family, member 8
-1.69	DAAM1	dishevelled associated activator of morphogenesis 1
-1.68	LOC102153923	major allergen I polypeptide chain 2-like
-1.68	LOC100686488	glutathione S-transferase theta-2B-like
-1.68	MIR193A	microRNA mir-193a
-1.67	HBEGF	heparin-binding EGF-like growth factor
-1.67	TSHZ3	teashirt zinc finger homeobox 3
-1.67	ITM2C	integral membrane protein 2C
-1.67	CLN6	ceroid-lipofuscinosis, neuronal 6, late infantile, variant

-1.67	EEF2K	eukaryotic elongation factor 2 kinase
-1.67	ABCA8	ATP-binding cassette, sub-family A (ABC1), member 8
-1.66	ABHD17B	abhydrolase domain containing 17B
-1.66	WIPF3	WAS/WASL interacting protein family, member 3
-1.66	ELMO1	engulfment and cell motility 1
-1.66	ASAH1	N-acylsphingosine amidohydrolase (acid ceramidase) 1
-1.66	HSPB8	heat shock 22kDa protein 8
-1.66	LOC609365	CD46 molecule, complement regulatory protein-like
-1.66	SGSH	N-sulfoglucosamine sulfohydrolase
-1.66	GRIA3	glutamate receptor, ionotropic, AMPA 3
-1.65	NET1	neuroepithelial cell transforming 1
-1.65	MAPT	microtubule-associated protein tau
-1.65	LOC487174	protocadherin beta-7
-1.65	KLF5	Kruppel-like factor 5 (intestinal)
-1.65	TIPARP	TCDD-inducible poly(ADP-ribose) polymerase
-1.65	DOCK10	dedicator of cytokinesis 10
-1.65	ZSCAN2	zinc finger and SCAN domain containing 2
-1.65	LIPC	lipase, hepatic
-1.65	AGRN	agrin
-1.65	LOC480491	GTP:AMP phosphotransferase AK3, mitochondrial
-1.65	LGALS9	lectin, galactoside-binding, soluble, 9
-1.65	PARP8	poly(ADP-ribose) polymerase family member 8
-1.64	PARK2	parkin RBR E3 ubiquitin protein ligase
-1.64	CTSL3	cathepsin L family member 3
-1.64	MFHAS1	malignant fibrous histiocytoma amplified sequence 1
-1.64	MAGI2	membrane associated guanylate kinase, WW and PDZ domain containing 2
-1.64	EPB41	erythrocyte membrane protein band 4.1
-1.63	LOC476208	putative PDZ domain-containing protein PDZK1P1
-1.63	LRP3	low density lipoprotein receptor-related protein 3
-1.63	FOSL2	FOS-like antigen 2
-1.63	SNORD99	Small nucleolar RNA SNORD99
-1.63	CMTM6	CKLF-like MARVEL transmembrane domain containing 6
-1.63	SLC5A3	solute carrier family 5 (sodium/myo-inositol cotransporter), member 3; sodium/myo-inositol cotransporter
-1.63	CHD3	chromodomain helicase DNA binding protein 3

-1.63	PLXNA3	plexin A3
-1.62	SSC5D	scavenger receptor cysteine rich family, 5 domains
-1.62	CPE	carboxypeptidase E
-1.62	ITPR1	inositol 1,4,5-trisphosphate receptor, type 1
-1.62	LRRFIP2	leucine rich repeat (in FLII) interacting protein 2
-1.62	SHOX2	short stature homeobox 2
-1.62	SLC35F3	solute carrier family 35, member F3
-1.62	ARV1	ARV1 homolog, fatty acid homeostatsis modulator
-1.62	SIK2	salt-inducible kinase 2
-1.62	NPC1	Niemann-Pick disease, type C1
-1.62	ZFP14	ZFP14 zinc finger protein
-1.61	PLCB1	phospholipase C, beta 1 (phosphoinositide-specific)
-1.61	SNX32	sorting nexin 32
-1.61	RAB3D	RAB3D, member RAS oncogene family
-1.61	TRIM47	tripartite motif containing 47; tripartite motif containing 65
-1.6	EPAS1	endothelial PAS domain protein 1
-1.6	REV3L	REV3-like, polymerase (DNA directed), zeta, catalytic subunit
-1.6	LECT1	leukocyte cell derived chemotaxin 1
-1.6	PHYHIPL	phytanoyl-CoA 2-hydroxylase interacting protein-like
-1.6	JAG2	jagged 2
-1.6	DHRS13	dehydrogenase/reductase (SDR family) member 13
-1.6	LOC607536	tyrosine-protein phosphatase non-receptor type substrate 1-like
-1.6	LOC610177	signal-regulatory protein beta-1 isoform 3-like
-1.59	ENSCAFG00000039130	Chromosome 1: 25,738,715-25,743,756
-1.59	METTL20	methyltransferase like 20
-1.59	MTMR10	myotubularin related protein 10
-1.59	SSFA2	sperm specific antigen 2
-1.58	GLIS3	GLIS family zinc finger 3
-1.58	SRGAP1	SLIT-ROBO Rho GTPase activating protein 1
-1.58	ADGRA2	adhesion G protein-coupled receptor A2
-1.58	MIR23A	microRNA mir-23a
-1.58	LRRC16A	leucine rich repeat containing 16A
-1.58	SNORD60	Small nucleolar RNA SNORD60
-1.58	SNORD77	Small nucleolar RNA SNORD77
-1.58	PDK2	pyruvate dehydrogenase kinase, isozyme 2

-1.57	MGAT4B	mannosyl (alpha-1,3-)-glycoprotein beta-1,4-N-acetylglucosaminyltransferase, isozyme B
-1.57	RRAGC	Ras-related GTP binding C
-1.57	BTF3L4	basic transcription factor 3-like 4
-1.57	STK17A	serine/threonine kinase 17a
-1.57	OSBPL5	oxysterol binding protein-like 5
-1.57	ANKRD28	ankyrin repeat domain 28
-1.57	PLBD2	phospholipase B domain containing 2
-1.57	FAM219B	family with sequence similarity 219, member B
-1.57	DISC1	disrupted in schizophrenia 1
-1.57	VAV2	vav 2 guanine nucleotide exchange factor
-1.57	C1QTNF5	C1q and TNF related 5
-1.56	MTMR11	myotubularin related protein 11
-1.56	FYCO1	FYVE and coiled-coil domain containing 1
-1.56	IGF2BP2	insulin-like growth factor 2 mRNA binding protein 2
-1.56	CTDSP1	CTD (carboxy-terminal domain, RNA polymerase II, polypeptide A) small phosphatase 1
-1.56	GALNS	galactosamine (N-acetyl)-6-sulfatase
-1.56	CORO7	coronin 7
-1.56	RPS6KA5	ribosomal protein S6 kinase, 90kDa, polypeptide 5
-1.56	MTCP1	mature T-cell proliferation 1
-1.55	ELL3	elongation factor RNA polymerase II-like 3
-1.55	CITED1	Cbp/p300-interacting transactivator, with Glu/Asp-rich carboxy-terminal domain, 1
-1.54	TSC22D1	TSC22 domain family, member 1
-1.54	FBXO46	F-box protein 46
-1.54	CAECAM1	carcinoembryonic antigen-related cell adhesion molecule 25
-1.54	SCN1B	sodium channel, voltage gated, type I beta subunit
-1.54	FEM1C	fem-1 homolog c (C. elegans)
-1.54	DENND2A	DENN/MADD domain containing 2A
-1.54	LIX1L	limb and CNS expressed 1 like
-1.54	ZBTB8A	zinc finger and BTB domain containing 8A
-1.54	CACNA2D2	calcium channel, voltage-dependent, alpha 2/delta subunit 2
-1.54	NOL4L	nucleolar protein 4-like
-1.54	FBXO25	F-box protein 25
-1.54	KIF21A	kinesin family member 21A
-1.54	ARRDC3	arrestin domain containing 3
-1.54	SOX4	SRY (sex determining region Y)-box 4

-1.54	FAM102B	family with sequence similarity 102, member B
-1.53	LIFR	leukemia inhibitory factor receptor alpha
-1.53	RHPN2	rhophilin, Rho GTPase binding protein 2
-1.53	TBC1D2	TBC1 domain family, member 2
-1.53	TMEM200B	transmembrane protein 200B
-1.53	PRKCD	protein kinase C, delta
-1.53	SIAH2	siah E3 ubiquitin protein ligase 2; glutamate-rich 6
-1.53	PLEKHA1	pleckstrin homology domain containing, family A (phosphoinositide binding specific) member 1
-1.53	HEG1	heart development protein with EGF-like domains 1
-1.53	DGKG	diacylglycerol kinase, gamma 90kDa
-1.53	TBL1XR1	transducin (beta)-like 1 X-linked receptor 1
-1.53	PLCD4	phospholipase C, delta 4
-1.53	TEX2	testis expressed 2
-1.53	LOC100682773	serine/threonine-protein kinase pim-3-like
-1.52	TNFAIP8	tumor necrosis factor, alpha-induced protein 8
-1.52	PTPN3	protein tyrosine phosphatase, non-receptor type 3
-1.52	HRH3	histamine receptor H3
-1.52	IQGAP2	IQ motif containing GTPase activating protein 2
-1.52	BANK1	B-cell scaffold protein with ankyrin repeats 1
-1.52	RPL13	ribosomal protein L13
-1.52	PTPN14	protein tyrosine phosphatase, non-receptor type 14
-1.52	TMEM164	transmembrane protein 164
-1.52	PTPN3	protein tyrosine phosphatase, non-receptor type 3
-1.51	TCP11L2	t-complex 11, testis-specific-like 2
-1.51	CD109	CD109 molecule
-1.51	C1GALT1	core 1 synthase, glycoprotein-N-acetylgalactosamine 3-beta-galactosyltransferase 1
-1.51	TRIB2	tribbles pseudokinase 2
-1.51	RYBP	RING1 and YY1 binding protein
-1.51	TGFBR2	transforming growth factor, beta receptor II (70/80kDa)
-1.51	TMEM116	transmembrane protein 116
-1.51	FAM53B	family with sequence similarity 53, member B
-1.51	ZCCHC24	zinc finger, CCHC domain containing 24
1.51	PABPC4	poly(A) binding protein, cytoplasmic 4 (inducible form)
1.51	INTU	inturned planar cell polarity protein
1.51	SPPL2B	signal peptide peptidase like 2B

1.51	POLD3	polymerase (DNA-directed), delta 3, accessory subunit
1.51	LGI3	leucine-rich repeat LGI family, member 3
1.51	RECQL	RecQ helicase-like
1.51	PSMD14	proteasome 26S subunit, non-ATPase 14
1.51	NSL1	NSL1, MIS12 kinetochore complex component
1.51	HM13	histocompatibility minor 13
1.52	ARMC9	armadillo repeat containing 9
1.52	SPIB	Spi-B transcription factor (Spi-1/PU.1 related); polymerase (DNA directed), delta 1, catalytic subunit
1.52	SNORA74	Small nucleolar RNA SNORA74
1.52	C15H1orf109	chromosome 15 open reading frame, human C1orf109
1.52	DNASE1L3	deoxyribonuclease I-like 3
1.52	DNMT1	DNA (cytosine-5-)-methyltransferase 1
1.52	FKBP4	FK506 binding protein 4, 59kDa
1.52	GLIS1	GLIS family zinc finger 1
1.52	ENSCAFG00000026475	Chromosome 6: 36,862,275-36,862,364
1.52	POLA1	polymerase (DNA directed), alpha 1, catalytic subunit
1.52	TREX2	three prime repair exonuclease 2
1.52	JPH1	junctophilin 1
1.53	GMFG	glia maturation factor, gamma
1.53	C1H9orf85	chromosome 1 open reading frame, human C9orf85
1.53	DQX1	DEAQ box RNA-dependent ATPase 1
1.53	SIPA1	signal-induced proliferation-associated 1
1.53	SORD	sorbitol dehydrogenase
1.53	DUT	deoxyuridine triphosphatase
1.53	TEX9	testis expressed 9
1.53	TPMT	thiopurine S-methyltransferase
1.54	SYMPK	symplekin
1.54	SLC22A15	solute carrier family 22, member 15
1.54	HNRNPM	heterogeneous nuclear ribonucleoprotein M
1.54	HSPD1	heat shock 60kDa protein 1 (chaperonin)
1.54	KPNA2	karyopherin alpha 2 (RAG cohort 1, importin alpha 1)
1.54	CD40LG	CD40 ligand
1.55	MIR340	microRNA mir-340
1.55	AUNIP	aurora kinase A and ninein interacting protein

1.55	ATP13A1	ATPase type 13A1
1.55	ARHGAP19	Rho GTPase activating protein 19
1.55	CCNE2	cyclin E2
1.55	SLF1	SMC5-SMC6 complex localization factor 1
1.55	PVRL3	poliovirus receptor-related 3
1.55	TPPP3	tubulin polymerization-promoting protein family member 3
1.55	FAM20C	family with sequence similarity 20, member C
1.55	MIR6516	microRNA mir-6516
1.56	FAM198B	family with sequence similarity 198, member B
1.56	FAM111A	family with sequence similarity 111, member A
1.56	HOMER2	homer scaffolding protein 2
1.56	ATP5G3	ATP synthase, H+ transporting, mitochondrial Fo complex, subunit C3 (subunit 9)
1.56	ATL1	atlastin GTPase 1
1.56	EMC9	ER membrane protein complex subunit 9
1.57	FAM13A	family with sequence similarity 13, member A
1.57	PRADC1	protease-associated domain containing 1
1.57	PSMA2	proteasome subunit alpha 2
1.57	NUDC	nudC nuclear distribution protein
1.57	GINS1	GINS complex subunit 1 (Psf1 homolog)
1.57	CCDC150	coiled-coil domain containing 150
1.57	MYL10	myosin, light chain 10, regulatory
1.57	PLEK2	pleckstrin 2
1.57	FUS	FUS RNA binding protein
1.57	ENSCAFG00000006007	Chromosome 21: 26,709,459-26,710,169
1.58	PNPLA3	patatin-like phospholipase domain containing 3
1.58	ITPA	inosine triphosphatase (nucleoside triphosphate pyrophosphatase)
1.58	SFXN2	sideroflexin 2
1.58	NDC1	NDC1 transmembrane nucleoporin
1.59	NHP2	NHP2 ribonucleoprotein
1.59	EMG1	EMG1 N1-specific pseudouridine methyltransferase
1.59	FUS	FUS RNA binding protein
1.59	BCAP31	B-cell receptor-associated protein 31
1.6	MIR107	microRNA mir-107
1.6	LYRM7	LYR motif containing 7
1.6	IFT74	intraflagellar transport 74
1.6	PPIH	peptidylprolyl isomerase H (cyclophilin H)

1.6	NCLN	nicalin
1.6	CAPN5	calpain 5
1.6	RPL27A	ribosomal protein L27a
1.6	NEK10	NIMA-related kinase 10
1.6	C24H20orf196	chromosome 24 open reading frame, human C20orf196
1.6	ALG5	ALG5, dolichyl-phosphate beta-glucosyltransferase
1.6	C1QBP	complement component 1, q subcomponent binding protein
1.6	COL11A1	collagen, type XI, alpha 1
1.6	SIVA1	SIVA1, apoptosis-inducing factor
1.61	EPHB3	EPH receptor B3
1.61	ANKRD1	ankyrin repeat domain 1 (cardiac muscle)
1.61	THOP1	thimet oligopeptidase 1
1.61	FDFT1	farnesyl-diphosphate farnesyltransferase 1
1.61	RHNO1	RAD9-HUS1-RAD1 interacting nuclear orphan 1
1.61	SLIRP	SRA stem-loop interacting RNA binding protein
1.61	FEN1	flap structure-specific endonuclease 1
1.62	CENPP	centromere protein P
1.62	ADSL	adenylosuccinate lyase
1.62	FAM49A	family with sequence similarity 49, member A
1.62	POC1A	POC1 centriolar protein A
1.62	XRCC4	X-ray repair complementing defective repair in Chinese hamster cells 4
1.62	DHODH	dihydroorotate dehydrogenase (quinone)
1.62	CCDC56	coiled-coil domain containing 56
1.63	RFX2	regulatory factor X, 2 (influences HLA class II expression)
1.63	LOC100856570	trifunctional purine biosynthetic protein adenosine-3; phosphoribosylglycinamide formyltransferase, phosphoribosylglycinamide synthetase, phosphoribosylaminoimidazole synthetase
1.63	INTS1	integrator complex subunit 1
1.63	MTHFD1	methylenetetrahydrofolate dehydrogenase (NADP+ dependent) 1, methenyltetrahydrofolate cyclohydrolase, formyltetrahydrofolate synthetase
1.63	BRIP1	BRCA1 interacting protein C-terminal helicase 1
1.64	ALDH1L2	aldehyde dehydrogenase 1 family, member L2
1.64	CYC1	cytochrome c-1
1.64	ORC6	origin recognition complex, subunit 6
1.64	DCLRE1B	DNA cross-link repair 1B

1.64	TMX2	thioredoxin-related transmembrane protein 2
1.64	CTXN1	cortexin 1
1.64	MDH2	malate dehydrogenase 2, NAD (mitochondrial)
1.64	SUV39H1	suppressor of variegation 3-9 homolog 1 (Drosophila)
1.65	MTFR2	mitochondrial fission regulator 2
1.65	MSH2	mutS homolog 2
1.65	RPA2	replication protein A2, 32kDa
1.65	TAF1D	TATA box binding protein (TBP)-associated factor, RNA polymerase I, D, 41kDa
1.65	RAN	RAN, member RAS oncogene family
1.65	NUSAP1	nucleolar and spindle associated protein 1
1.65	CEP152	centrosomal protein 152kDa
1.65	RTKN2	rhotekin 2
1.65	HYOU1	hypoxia up-regulated 1
1.66	ADCY7	adenylate cyclase 7
1.66	TOMM40	translocase of outer mitochondrial membrane 40 homolog (yeast)
1.66	FILIP1	filamin A interacting protein 1
1.66	LOC483167	histone H3-like
1.66	RRM1	ribonucleotide reductase M1
1.66	MCM8	minichromosome maintenance 8 homologous recombination repair factor
1.66	HSD3B7	hydroxy-delta-5-steroid dehydrogenase, 3 beta- and steroid delta-isomerase 7
1.66	DBF4B	DBF4 zinc finger B
1.67	C1QTNF3	C1q and tumor necrosis factor related protein 3; alpha-methylacyl-CoA racemase
1.67	CYP51A1	cytochrome P450, family 51, subfamily A, polypeptide 1
1.67	MFSD2A	major facilitator superfamily domain containing 2A
1.67	LIG1	ligase I, DNA, ATP-dependent
1.67	TCF19	transcription factor 19
1.67	C18H11orf98	chromosome 18 open reading frame, human C11orf98
1.67	NOP56	NOP56 ribonucleoprotein
1.67	WDR76	WD repeat domain 76
1.67	GRIK4	glutamate receptor, ionotropic, kainate 4
1.67	TMEM97	transmembrane protein 97
1.68	ENSCAFG00000027833	Chromosome 32: 15,870,940-15,871,023
1.69	PMEPA1	prostate transmembrane protein, androgen induced 1

1.69	TYMS	thymidylate synthetase
1.69	TLN2	talin 2
1.69	POLQ	polymerase (DNA directed), theta
1.69	HMGB3	high mobility group box 3
1.7	VSTM2B	V-set and transmembrane domain containing 2B
1.7	CDC23	cell division cycle 23
1.7	PRIM2	primase, DNA, polypeptide 2 (58kDa)
1.7	RHOD	ras homolog family member D
1.7	HACD1	protein tyrosine phosphatase-like (proline instead of catalytic arginine), member A
1.7	SQRDL	sulfide quinone reductase-like (yeast)
1.7	LOC488263	histone H3.1
1.7	LOC488277	histone H4
1.71	VDR	vitamin D (1,25- dihydroxyvitamin D3) receptor
1.71	RAD18	RAD18 E3 ubiquitin protein ligase
1.71	ACSL5	acyl-CoA synthetase long-chain family member 5
1.71	C7H1orf112	chromosome 7 open reading frame, human C1orf112
1.72	FAM206A	family with sequence similarity 206, member A
1.72	GPRC5A	G protein-coupled receptor, class C, group 5, member A
1.72	LOC488260	uncharacterized LOC488260
1.72	FASN	fatty acid synthase
1.72	NSDHL	NAD(P) dependent steroid dehydrogenase-like
1.73	NPM3	nucleophosmin/nucleoplasmin 3
1.74	HSD17B12	hydroxysteroid (17-beta) dehydrogenase 12
1.74	FANCM	Fanconi anemia, complementation group M
1.74	VRK1	vaccinia related kinase 1
1.74	FAM122B	family with sequence similarity 122B
1.75	TSPAN6	tetraspanin 6
1.75	NPM1	Chromosome 4: 40,756,782-40,770,088
1.75	CCDC167	coiled-coil domain containing 167
1.75	CLSPN	claspin
1.76	E2F7	E2F transcription factor 7
1.76	LDLR	low density lipoprotein receptor
1.76	TAF1D	TATA box binding protein (TBP)-associated factor, RNA polymerase I, D, 41kDa
1.76	PLS1	plastin 1
1.76	PDLIM7	PDZ and LIM domain 7 (enigma)

1.77	TMPO	thymopoietin
1.77	SNORD123	Small nucleolar RNA SNORD123
1.77	HSD17B7	hydroxysteroid (17-beta) dehydrogenase 7
1.77	TMEM107	transmembrane protein 107
1.77	GAS8	growth arrest-specific 8
1.78	SLC29A1	solute carrier family 29 (equilibrative nucleoside transporter), member 1; heat shock protein 90kDa alpha (cytosolic), class B member 1
1.78	IL15RA	interleukin 15 receptor, alpha
1.78	SLC38A5	solute carrier family 38, member 5
1.79	ETHE1	ethylmalonic encephalopathy 1
1.79	PHF19	PHD finger protein 19
1.79	DOCK3	dedicator of cytokinesis 3
1.79	SLCO3A1	solute carrier organic anion transporter family, member 3A1
1.79	PFAS	phosphoribosylformylglycinamidine synthase
1.79	DTD2	D-tyrosyl-tRNA deacylase 2 (putative)
1.8	YIF1B	Yip1 interacting factor homolog B (S. cerevisiae)
1.8	SMC2	structural maintenance of chromosomes 2
1.8	SNORD48	Small nucleolar RNA SNORD48
1.8	CAMK2A	calcium/calmodulin-dependent protein kinase II alpha
1.81	PAPPA	pregnancy-associated plasma protein A, pappalysin 1
1.81	KIF24	kinesin family member 24
1.81	LOC486670	histone H4
1.81	HELLS	helicase, lymphoid-specific
1.81	NPTX2	neuronal pentraxin II
1.82	UACA	uveal autoantigen with coiled-coil domains and ankyrin repeats
1.82	HMGB2	high mobility group box 2
1.82	MCM4	minichromosome maintenance complex component 4
1.82	GTPBP8	GTP-binding protein 8 (putative)
1.83	CKAP4	cytoskeleton-associated protein 4
1.83	TRAIP	TRAF interacting protein
1.83	SCARB1	scavenger receptor class B, member 1
1.83	FKBP11	FK506 binding protein 11
1.84	GCAT	glycine C-acetyltransferase

1.84	SEMA5A	sema domain, seven thrombospondin repeats (type 1 and type 1-like), transmembrane domain (TM) and short cytoplasmic domain, (semaphorin) 5A
1.84	USP1	ubiquitin specific peptidase 1
1.84	RFC2	replication factor C (activator 1) 2, 40kDa
1.84	NGFR	nerve growth factor receptor
1.85	NDUFB2	NADH dehydrogenase (ubiquinone) 1 beta subcomplex, 2, 8kDa
1.85	RFC5	replication factor C (activator 1) 5, 36.5kDa
1.85	SLCO3A1	solute carrier organic anion transporter family, member 3A1
1.85	ZWILCH	zwilch kinetochore protein
1.85	CTH	cystathionine gamma-lyase
1.85	RALGPS2	Ral GEF with PH domain and SH3 binding motif 2
1.86	SNRPA	small nuclear ribonucleoprotein polypeptide A
1.86	KIF18A	kinesin family member 18A
1.86	ENSCAFG00000015856	Chromosome 8: 38,816,649-38,817,107
1.87	WDR62	WD repeat domain 62
1.87	CCL27	chemokine (C-C motif) ligand 27
1.88	SREBF1	sterol regulatory element binding transcription factor 1
1.88	CACNG7	calcium channel, voltage-dependent, gamma subunit 7
1.88	PARPBP	PARP1 binding protein
1.88	INCENP	inner centromere protein antigens 135/155kDa
1.88	MASTL	microtubule associated serine/threonine kinase-like
1.89	C28H10orf90	chromosome 28 open reading frame, human C10orf90
1.9	RCOR2	REST corepressor 2
1.9	EXO1	exonuclease 1
1.9	RPS29	ribosomal protein S29
1.91	SLC9B1	solute carrier family 9, subfamily B (NHA1, cation proton antiporter 1), member 1
1.93	PRIM1	primase, DNA, polypeptide 1 (49kDa)
1.93	TRIM34	tripartite motif containing 34
1.93	BRCA2	breast cancer 2, early onset
1.93	CLDN6	claudin 6
1.95	SPC24	SPC24, NDC80 kinetochore complex component
1.95	FANCA	Fanconi anemia, complementation group A
1.96	ACOT7	acyl-CoA thioesterase 7
1.97	CENPO	centromere protein O

1.97	RFC3	replication factor C (activator 1) 3, 38kDa
1.97	NCAPD2	non-SMC condensin I complex, subunit D2
1.97	GPR143	G protein-coupled receptor 143
1.98	LPCAT3	lysophosphatidylcholine acyltransferase 3
1.98	SMTN	smoothelin
1.98	LOC608051	tubulin alpha-3 chain
1.98	FAM110B	family with sequence similarity 110, member B
1.98	KIAA1524	KIAA1524 ortholog
1.98	LHX9	LIM homeobox 9
1.99	CENPN	centromere protein N
1.99	TLCD1	TLC domain containing 1
2	FZD2	frizzled class receptor 2
2	ARHGEF39	Rho guanine nucleotide exchange factor (GEF) 39
2	SLC7A2	solute carrier family 7 (cationic amino acid transporter, y+ system), member 2
2.01	IGDCC4	immunoglobulin superfamily, DCC subclass, member 4
2.01	TUBD1	tubulin, delta 1
2.01	HAUS7	HAUS augmin-like complex, subunit 7
2.01	NUSAP1	nucleolar and spindle associated protein 1
2.02	KCND2	potassium channel, voltage gated Shal related subfamily D, member 2
2.02	PCDH18	protocadherin 18
2.02	MICAL2	microtubule associated monooxygenase, calponin and LIM domain containing 2
2.02	CD40	CD40 molecule, TNF receptor superfamily member 5
2.02	IQGAP3	IQ motif containing GTPase activating protein 3
2.03	GEN1	GEN1 Holliday junction 5 flap endonuclease
2.03	LOC488258	histone H1.1
2.04	LGR5	leucine-rich repeat containing G protein-coupled receptor 5
2.04	DDIAS	DNA damage-induced apoptosis suppressor
2.04	NUDT1	nudix (nucleoside diphosphate linked moiety X)-type motif 1
2.05	CRABP2	cellular retinoic acid binding protein 2
2.05	RUNX1T1	runt-related transcription factor 1; translocated to, 1 (cyclin D-related)
2.06	FAM83D	family with sequence similarity 83, member D
2.06	SPAG5	sperm associated antigen 5
2.08	MEOX1	mesenchyme homeobox 1

2.08	AKAP2	A kinase (PRKA) anchor protein 2
2.08	CENPA	centromere protein A
2.1	CPXM2	carboxypeptidase X (M14 family), member 2
2.12	KIF18B	kinesin family member 18B
2.12	FBXO27	F-box protein 27
2.12	MCM5	minichromosome maintenance complex component 5
2.12	KIF14	kinesin family member 14
2.13	CDKN2C	cyclin-dependent kinase inhibitor 2C (p18, inhibits CDK4)
2.13	NDUFS8	NADH dehydrogenase (ubiquinone) Fe-S protein 8, 23kDa (NADH-coenzyme Q reductase)
2.13	FAM72A	family with sequence similarity 72, member A
2.14	LOC479820	exonuclease NEF-sp
2.14	CDC6	cell division cycle 6
2.15	DHCR7	7-dehydrocholesterol reductase
2.15	BLM	Bloom syndrome, RecQ helicase-like
2.15	ACLY	ATP citrate lyase
2.16	SNORD26	Small nucleolar RNA SNORD26
2.16	LOC102151147	uncharacterized LOC102151147
2.17	CENPM	centromere protein M
2.18	MYH10	myosin, heavy chain 10, non-muscle
2.19	AMPH	amphiphysin
2.19	SNRPA1	small nuclear ribonucleoprotein polypeptide A
2.2	ZC4H2	zinc finger, C4H2 domain containing
2.21	LIMS2	LIM and senescent cell antigen-like domains 2
2.21	TRIP13	thyroid hormone receptor interactor 13
2.22	AP3M2	adaptor-related protein complex 3, mu 2 subunit
2.22	C17H1orf54	chromosome 17 open reading frame, human C1orf54
2.22	VWA5A	von Willebrand factor A domain containing 5A
2.23	CENPE	centromere protein E, 312kDa
2.24	KIF22	kinesin family member 22
2.25	REPS2	RALBP1 associated Eps domain containing 2
2.28	ACAT2	acetyl-CoA acetyltransferase 2
2.28	NCAPH	non-SMC condensin I complex, subunit H
2.29	KIAA1456	KIAA1456 ortholog
2.29	ESPL1	extra spindle pole bodies like 1, separase
2.29	LOC102152580	armadillo repeat-containing protein 4-like

2.3	CNOT1	CCR4-NOT transcription complex subunit 1
2.3	MARK1	MAP/microtubule affinity-regulating kinase 1
2.31	KIF2C	kinesin family member 2C
2.32	MATN2	matrilin 2
2.32	SGOL1	shugoshin-like 1 (S. pombe)
2.34	KIF20B	kinesin family member 20B
2.34	UNC5B	unc-5 netrin receptor B
2.35	POLA2	polymerase (DNA directed), alpha 2, accessory subunit
2.36	INSIG1	insulin induced gene 1
2.36	CRYL1	crystallin, lambda 1
2.37	C20H3orf14	chromosome 20 open reading frame, human C3orf14
2.37	TICRR	TOPBP1-interacting checkpoint and replication regulator
2.37	CENPI	centromere protein I
2.38	AURKB	aurora kinase B
2.4	RAD51AP1	RAD51 associated protein 1
2.41	TK1	thymidine kinase 1, soluble
2.43	MYO3A	myosin IIIA
2.44	VEGFC	vascular endothelial growth factor C
2.44	KIF15	kinesin family member 15
2.44	CCDC150	coiled-coil domain containing 150
2.44	RAVER2	ribonucleoprotein, PTB-binding 2
2.44	RMI2	RecQ mediated genome instability 2
2.45	RFC4	replication factor C (activator 1) 4, 37kDa
2.46	TPX2	TPX2, microtubule-associated
2.46	REEP4	receptor accessory protein 4
2.47	COL15A1	collagen, type XV, alpha 1
2.47	TCF21	transcription factor 21
2.47	MCM2	minichromosome maintenance complex component 2
2.48	ORC1	origin recognition complex, subunit 1
2.5	SULF1	sulfatase 1
2.5	CDT1	chromatin licensing and DNA replication factor 1
2.51	PLA2R1	phospholipase A2 receptor 1, 180kDa
2.52	DTL	denticleless E3 ubiquitin protein ligase homolog (Drosophila)
2.53	RAD54L	RAD54-like (S. cerevisiae)
2.54	MYL9	myosin, light chain 9, regulatory

2.55	OIP5	Opa interacting protein 5
2.56	ALDH1A3	A kinase (PRKA) anchor protein 12
2.56	FAM64A	family with sequence similarity 64, member A
2.57	SP100	SP100 nuclear antigen
2.58	ANLN	anillin actin binding protein
2.58	FDPS	farnesyl diphosphate synthase
2.59	FGD5	FYVE, RhoGEF and PH domain containing 5
2.59	E2F1	E2F transcription factor 1
2.59	HMMR	hyaluronan-mediated motility receptor (RHAMM)
2.62	LAMA2	laminin, alpha 2
2.62	RARRES2	retinoic acid receptor responder (tazarotene induced) 2
2.64	LOC102153827	uncharacterized LOC102153827
2.65	RAD51	RAD51 recombinase
2.67	MCM3	minichromosome maintenance complex component 3
2.7	WFIKKN2	WAP, follistatin/kazal, immunoglobulin, kunitz and netrin domain containing 2
2.7	UHRF1	ubiquitin-like with PHD and ring finger domains 1
2.73	FANCD2	Fanconi anemia, complementation group D2
2.73	FANCI	Fanconi anemia, complementation group I
2.73	MYLK	myosin light chain kinase
2.73	PLK1	polo-like kinase 1
2.74	BTK	Bruton agammaglobulinemia tyrosine kinase
2.75	MELK	maternal embryonic leucine zipper kinase
2.75	TUBA4A	tubulin, alpha 4a
2.75	LOC489372	histone H2AX
2.76	TTK	TTK protein kinase
2.76	CDCA8	cell division cycle associated 8
2.76	ARHGAP11A	Rho GTPase activating protein 11A
2.79	CCNB3	cyclin B3
2.8	CCNF	cyclin F
2.8	BRCA1	breast cancer 1, early onset
2.81	KNTC1	kinetochore associated 1
2.85	CASC5	cancer susceptibility candidate 5
2.86	CDCA2	cell division cycle associated 2
2.86	LOC489024	core histone macro-H2A.2
2.86	SASS6	SAS-6 centriolar assembly protein
2.87	BIRC5	baculoviral IAP repeat containing 5

2.88	ITGBL1	integrin, beta-like 1 (with EGF-like repeat domains)
2.9	TPM2	tropomyosin 2
2.9	CEP55	centrosomal protein 55kDa
2.9	PKMYT1	protein kinase, membrane associated tyrosine/threonine 1
2.92	BUB1B	BUB1 mitotic checkpoint serine/threonine kinase B
2.92	LOC100856294	rho GTPase-activating protein 20-like
2.92	KIF4A	kinesin family member 4A
2.93	PAK1	p21 protein (Cdc42/Rac)-activated kinase 1
2.94	CDC20	cell division cycle 20
2.94	FOXM1	forkhead box M1
2.94	TACC3	transforming, acidic coiled-coil containing protein 3; transforming acidic coiled-coil-containing protein 3-like
2.96	HOXD8	homeobox D8
2.97	UBE2T	ubiquitin-conjugating enzyme E2T
2.99	TM7SF2	transmembrane 7 superfamily member 2
3.02	POLE	polymerase (DNA directed), epsilon, catalytic subunit
3.02	NME1	non-metastatic cells 1, protein (NM23A) expressed in
3.02	LOC102153443	rho GTPase-activating protein 20-like
3.03	KNSTRN	kinetochore-localized astrin/SPAG5 binding protein
3.03	DHCR24	24-dehydrocholesterol reductase
3.05	PI16	peptidase inhibitor 16
3.05	KIF11	kinesin family member 11
3.05	TOP2A	topoisomerase (DNA) II alpha
3.07	TPM2	tropomyosin 2 (beta)
3.09	CENPU	centromere protein U
3.1	ST5	suppression of tumorigenicity 5
3.1	LAMA2	laminin, alpha 2
3.11	DIAPH3	diaphanous-related formin 3
3.12	KIF23	kinesin family member 23
3.15	RACGAP1	Rac GTPase activating protein 1
3.15	DLGAP5	discs, large (Drosophila) homolog-associated protein 5
3.16	MYBL2	v-myb avian myeloblastosis viral oncogene homolog-like 2
3.2	CH25H	cholesterol 25-hydroxylase
3.2	TMEM88	transmembrane protein 88

3.25	GTSE1	G-2 and S-phase expressed 1
3.26	CCNB1	cyclin B1
3.27	CENPF	centromere protein F, 350/400kDa
3.27	CLSPN	claspin
3.28	MCM10	minichromosome maintenance 10 replication initiation factor
3.29	CENPT	centromere protein T
3.29	GPER1	G protein-coupled estrogen receptor 1
3.3	NCAPG	non-SMC condensin I complex, subunit G
3.31	ALDH1A3	aldehyde dehydrogenase 1 family, member A3
3.32	NOX4	NADPH oxidase 4
3.32	DLA-64	MHC class I DLA-64
3.34	CDC45	cell division cycle 45
3.34	PRR11	proline rich 11
3.36	GPC4	glypican 4
3.38	LOC102152154	rho GTPase-activating protein 20-like; uncharacterized LOC102151972
3.39	CIT	citron rho-interacting serine/threonine kinase
3.4	BUB1	BUB1 mitotic checkpoint serine/threonine kinase
3.42	KIFC1	kinesin family member C1
3.43	PTGFR	prostaglandin F receptor (FP)
3.44	NDC80	NDC80 kinetochore complex component
3.47	FABP3	fatty acid binding protein 3, muscle and heart
3.48	CCNB2	cyclin B2
3.5	RAB38	RAB38, member RAS oncogene family
3.52	ASPM	abnormal spindle microtubule assembly
3.54	LOC100855995	tubulin alpha chain-like
3.55	GFPT2	glutamine-fructose-6-phosphate transaminase 2
3.55	MIS18A	MIS18 kinetochore protein A
3.63	CCNA2	cyclin A2
3.64	DEPDC1	DEP domain containing 1
3.65	ZFHX4	zinc finger homeobox 4
3.66	SRPX2	sushi-repeat containing protein, X-linked 2
3.67	ECT2	epithelial cell transforming 2
3.72	SPC25	SPC25, NDC80 kinetochore complex component
3.73	ASF1B	anti-silencing function 1B histone chaperone
3.75	LOC102152056	antigen KI-67-like; antigen identified by monoclonal antibody Ki-67
3.78	TAGLN	transgelin

3.78	FAM20A	family with sequence similarity 20, member A
3.79	SHCBP1	SHC SH2-domain binding protein 1
3.8	LOC486400	gamma-glutamyltranspeptidase 1
3.83	PBK	PDZ binding kinase
3.85	PTTG1	pituitary tumor-transforming 1
3.86	CKAP2L	cytoskeleton associated protein 2-like
3.87	DIAPH3	diaphanous-related formin 3
3.89	KIF20A	kinesin family member 20A
3.89	SLC8A1	solute carrier family 8 (sodium/calcium exchanger), member 1
3.89	E2F8	E2F transcription factor 8
3.93	CDKN3	cyclin-dependent kinase inhibitor 3
3.99	PLCXD2	phosphatidylinositol-specific phospholipase C, X domain containing 2
4.02	PRC1	protein regulator of cytokinesis 1
4.04	LOC102151762	uncharacterized LOC102151762
4.05	TROAP	trophinin associated protein
4.08	NEK2	NIMA-related kinase 2
4.17	LOC102153827	uncharacterized LOC102153827
4.22	CDCA3	cell division cycle associated 3
4.29	KIAA0101	KIAA0101 ortholog; casein kinase 1, gamma 1
4.34	ARSE	arylsulfatase E (chondrodysplasia punctata 1)
4.37	ESM1	endothelial cell-specific molecule 1
4.4	UBE2C	ubiquitin-conjugating enzyme E2C
4.56	PEG3	paternally expressed 3
4.56	HJURP	Holliday junction recognition protein
4.74	ACPP	acid phosphatase, prostate
4.76	NUF2	NUF2, NDC80 kinetochore complex component
5	RNF144A	ring finger protein 144A
5.04	ESCO2	establishment of sister chromatid cohesion N-acetyltransferase 2
5.1	MAB21L2	mab-21-like 2 (C. elegans)
5.13	GNG11	guanine nucleotide binding protein (G protein), gamma 11
5.17	LOC491373	uncharacterized LOC491373
5.22	RRM2	ribonucleotide reductase M2
5.42	MDGA2	MAM domain containing glycosylphosphatidylinositol anchor 2
5.78	EPHA3	EPH receptor A3
5.81	ITGA8	integrin, alpha 8

6.73	FAP	fibroblast activation protein, alpha
7.09	MRAP	melanocortin 2 receptor accessory protein
7.52	CNN1	calponin 1, basic, smooth muscle
7.69	ACTA2	actin, alpha 2, smooth muscle, aorta
7.91	THBS2	thrombospondin 2
9.16	NEDD9	neural precursor cell expressed, developmentally down-regulated 9
9.21	TFPI2	tissue factor pathway inhibitor 2
9.52	MOXD1	monooxygenase, DBH-like 1
11.14	DKK2	dickkopf WNT signaling pathway inhibitor 2
31.45	ACTG2	actin, gamma 2, smooth muscle, enteric
