# Supplementary material for: Evaluation of canine 2D cell cultures as models of myxomatous mitral valve degeneration
Source: PLoS One. 2019 Aug 15;14(8):e0221126. doi: 10.1371/journal.pone.0221126 (PMC6695117; doi:10.1371/journal.pone.0221126)
Supplement: S4 Table — (PDF) [file pone.0221126.s004.pdf]

S4 Table. Gene list TGFβ1-treated qVICs vs qVICs with fold change < or > 1.5 (275 differentially expressed genes; 144 down, 131 up)

Fold Change	Gene Symbol	Description
-5.47	ENSCAFG00000031869	Uncharacterised
-5.11	PDK4	pyruvate dehydrogenase kinase, isozyme 4
-4.41	NDP	Norrie disease (pseudoglioma)
-4.19	NPPC	natriuretic peptide C
-4.1	FRMD5	FERM domain containing 5
-3.91	SLC43A2	solute carrier family 43 (amino acid system L transporter), member 2
-3.66	ACKR3	atypical chemokine receptor 3
-3.5	NRG1	neuregulin 1
-3.41	CCDC3	coiled-coil domain containing 3
-3.39	GJA5	gap junction protein, alpha 5, 40kDa
-3.09	BMPER	BMP binding endothelial regulator
-3.02	MALL	mal, T-cell differentiation protein-like
-3.01	RRAD	Ras-related associated with diabetes
-2.93	CCDC69	coiled-coil domain containing 69
-2.87	ZDHHC14	zinc finger, DHHC-type containing 14
-2.8	ATF7IP2	activating transcription factor 7 interacting protein 2
-2.77	SPINT1	serine peptidase inhibitor, Kunitz type 1
-2.74	STRIP2	striatin interacting protein 2
-2.73	APOL5	apolipoprotein L5
-2.7	CLIC5	chloride intracellular channel 5
-2.69	ARL4D	ADP-ribosylation factor-like 4D
-2.6	OLFML2A	olfactomedin-like 2A
-2.54	MYZAP	myocardial zonula adherens protein
-2.48	SLC25A42	solute carrier family 25, member 42
-2.41	SCARA5	scavenger receptor class A, member 5
-2.38	CNKSR3	CNKSR family member 3
-2.3	LDB2	LIM domain binding 2
-2.26	PTX3	pentraxin 3, long
-2.26	CYGB	cytoglobin
-2.2	EBF2	early B-cell factor 2
-2.18	FAM101A	family with sequence similarity 101, member A
-2.17	ZDHHC14	zinc finger, DHHC-type containing 14

-2.16	ITIH4	inter-alpha-trypsin inhibitor heavy chain family, member 4
-2.14	KCND3	potassium channel, voltage gated Shal related subfamily D, member 3
-2.14	P2RY1	purinergic receptor P2Y, G-protein coupled, 1
-2.13	F2RL2	coagulation factor II (thrombin) receptor-like 2
-2.13	LDB2	LIM domain binding 2
-2.11	EBF2	early B-cell factor 2
-2.07	FIGF	c-fos induced growth factor (vascular endothelial growth factor D)
-2.06	SELL	selectin L
-2.05	SAMHD1	SAM domain and HD domain 1
-2.02	FHDC1	FH2 domain containing 1
-2.02	PCDH7	protocadherin 7
-2.01	SLC16A10	solute carrier family 16 (aromatic amino acid transporter), member 10
-2.01	TNFRSF19	tumor necrosis factor receptor superfamily, member 19
-1.97	ITGA6	integrin, alpha 6
-1.96	IGF1	insulin-like growth factor 1 (somatomedin C)
-1.96	SLC10A6	solute carrier family 10 (sodium/bile acid cotransporter), member 6
-1.95	NET1	neuroepithelial cell transforming 1
-1.95	EPHA3	EPH receptor A3
-1.93	LGALSL	lectin, galactoside-binding-like
-1.91	ENSCAFG00000004270	Chromosome 21: 8,538,851-8,540,378
-1.91	ITGB4	integrin, beta 4
-1.88	LMO2	LIM domain only 2 (rhombotin-like 1)
-1.87	FAM19A4	family with sequence similarity 19 (chemokine (C-C motif)-like), member A4
-1.86	ADGRF2	adhesion G protein-coupled receptor F2
-1.84	RAPH1	Ras association (RalGDS/AF-6) and pleckstrin homology domains 1
-1.83	HS3ST1	heparan sulfate-glucosamine 3-sulfotransferase 1
-1.83	VCAM1	vascular cell adhesion molecule 1
-1.83	YPEL1	yippee-like 1
-1.83	PDE1A	phosphodiesterase 1A, calmodulin-dependent
-1.81	GRAP2	GRB2-related adaptor protein 2
-1.81	PRSS12	protease, serine 12
-1.81	PRSS12	protease, serine 12
-1.81	PRSS12	protease, serine 12

-1.81	ANKRD44	ankyrin repeat domain 44
-1.8	CSF1	colony stimulating factor 1 (macrophage)
-1.8	CABLES1	Cdk5 and Abl enzyme substrate 1
-1.79	ATOH8	atonal bHLH transcription factor 8
-1.78	SORBS1	sorbin and SH3 domain containing 1
-1.78	NRP1	neuropilin 1
-1.78	FRMD3	FERM domain containing 3
-1.78	RND3	Rho family GTPase 3
-1.78	CX3CL1	chemokine (C-X3-C motif) ligand 1
-1.78	AHNAK2	AHNAK nucleoprotein 2
-1.77	PROCR	protein C receptor, endothelial
-1.77	ARHGAP6	Rho GTPase activating protein 6
-1.76	LIFR	leukemia inhibitory factor receptor alpha
-1.76	PREX2	phosphatidylinositol-3,4,5-trisphosphate-dependent Rac exchange factor 2
-1.76	PPP1R36	protein phosphatase 1, regulatory subunit 36
-1.75	LYPD6	LY6/PLAUR domain containing 6
-1.75	SLC2A12	solute carrier family 2 (facilitated glucose transporter), member 12
-1.74	ZFP30	ZFP30 zinc finger protein
-1.73	NFKBIA	nuclear factor of kappa light polypeptide gene enhancer in B-cells inhibitor, alpha
-1.73	HBEGF	heparin-binding EGF-like growth factor
-1.73	MMP28	matrix metallopeptidase 28
-1.72	HSPA2	heat shock 70kDa protein 2
-1.72	MEF2C	myocyte enhancer factor 2C
-1.72	ABCC1	ATP-binding cassette, sub-family C (CFTR/MRP), member 1
-1.71	CACNG4	calcium channel, voltage-dependent, gamma subunit 4
-1.71	APOL5	apolipoprotein L5
-1.7	ST3GAL1	ST3 beta-galactoside alpha-2,3-sialyltransferase 1
-1.7	MEST	mesoderm specific transcript
-1.69	KLF15	Kruppel-like factor 15
-1.69	SLC40A1	solute carrier family 40 (iron-regulated transporter), member 1
-1.69	SYNE3	spectrin repeat containing, nuclear envelope family member 3
-1.68	CXADR	coxsackie virus and adenovirus receptor
-1.68	ADH4	alcohol dehydrogenase 4
-1.67	SLC22A23	solute carrier family 22, member 23

-1.67	PLCB1	phospholipase C, beta 1 (phosphoinositide-specific)
-1.66	TCF7	transcription factor 7 (T-cell specific, HMG-box)
-1.65	MAFB	v-maf avian musculoaponeurotic fibrosarcoma oncogene homolog B
-1.65	CBX7	chromobox homolog 7
-1.63	MAPT	microtubule-associated protein tau
-1.62	LOC474850	heat shock 70 kDa protein 1-like
-1.62	ADH4	alcohol dehydrogenase 4
-1.61	GPCPD1	glycerophosphocholine phosphodiesterase 1
-1.61	SEZ6	seizure related 6 homolog (mouse)
-1.6	ADAMTS1	ADAM metallopeptidase with thrombospondin type 1 motif, 1
-1.6	RASL10B	RAS-like, family 10, member B
-1.6	PIP4K2A	phosphatidylinositol-5-phosphate 4-kinase, type II, alpha
-1.6	CAPN5	calpain 5
-1.6	SYNDIG1	synapse differentiation inducing 1
-1.59	EPAS1	endothelial PAS domain protein 1
-1.59	EML2	echinoderm microtubule associated protein like 2
-1.59	SLC16A5	solute carrier family 16 (monocarboxylate transporter), member 5
-1.58	TPX2	TPX2, microtubule-associated
-1.57	ANLN	anillin actin binding protein
-1.57	ZC3HAV1	zinc finger CCCH-type, antiviral 1
-1.57	EPB41L4A	erythrocyte membrane protein band 4.1 like 4A
-1.56	TSPAN13	tetraspanin 13
-1.56	TSC22D1	TSC22 domain family, member 1
-1.56	HAND2	heart and neural crest derivatives expressed 2
-1.56	LIMCH1	LIM and calponin homology domains 1
-1.55	PPAP2B	phosphatidic acid phosphatase type 2B
-1.55	CTTNBP2	cortactin binding protein 2
-1.55	GCNT7	glucosaminyl (N-acetyl) transferase family member 7
-1.55	HSD11B1	hydroxysteroid (11-beta) dehydrogenase 1
-1.55	ADH4	alcohol dehydrogenase 4
-1.54	PLXND1	plexin D1
-1.54	ENSCAFG00000027807	Chromosome 33: 12,503,220-12,503,314
-1.53	KIF18B	kinesin family member 18B
-1.52	LOC481227	neuronal-specific septin-3

-1.52	ENSCAFG00000022342	Chromosome 1: 56,225,495-56,225,821
-1.52	MAMLD1	mastermind like domain containing 1
-1.52	CTTNBP2	cortactin binding protein 2
-1.52	LYVE1	lymphatic vessel endothelial hyaluronan receptor 1
-1.52	LOC477562	proline dehydrogenase 1, mitochondrial
-1.52	SEPT11	septin 11
-1.52	CDKL5	cyclin-dependent kinase-like 5
-1.52	EFNB1	ephrin-B1
-1.51	MET	MET proto-oncogene, receptor tyrosine kinase
-1.51	PLCB1	phospholipase C, beta 1 (phosphoinositide-specific)
-1.51	DGKD	diacylglycerol kinase, delta 130kDa
1.51	ADAM12	ADAM metallopeptidase domain 12
1.51	RASAL2	RAS protein activator like 2
1.51	COL1A1	collagen, type I, alpha 1
1.52	BTG1	B-cell translocation gene 1, anti-proliferative
1.52	BMP6	bone morphogenetic protein 6
1.52	HHIPL1	HHIP-like 1
1.53	LPCAT3	lysophosphatidylcholine acyltransferase 3
1.53	ENSCAFG00000027228	Chromosome 3: 28,138,305-28,138,408
1.53	RPS3	40S ribosomal protein S3
1.53	XPR1	xenotropic and polytropic retrovirus receptor 1
1.54	C12H6orf25	chromosome 12 open reading frame, human C6orf25
1.54	MYO1D	myosin ID
1.54	LOC480926	melanoma-associated antigen D4
1.54	UGDH	UDP-glucose 6-dehydrogenase
1.54	UGDH	UDP-glucose 6-dehydrogenase
1.54	UGDH	UDP-glucose 6-dehydrogenase
1.55	SREBF1	sterol regulatory element binding transcription factor 1
1.55	ABCA1	ATP-binding cassette, sub-family A (ABC1), member 1
1.55	PIM1	Pim-1 proto-oncogene, serine/threonine kinase
1.55	CTHRC1	collagen triple helix repeat containing 1
1.55	RASSF1	Ras association (RalGDS/AF-6) domain family member 1
1.55	SNORA1	Small nucleolar RNA SNORA1
1.55	ALDH18A1	aldehyde dehydrogenase 18 family, member A1
1.55	MRC2	mannose receptor, C type 2

1.56	VDR	vitamin D (1,25- dihydroxyvitamin D3) receptor
1.56	SMTN	smoothelin
1.56	TBC1D1	TBC1 (tre-2/USP6, BUB2, cdc16) domain family, member 1
1.56	SLC35A2	solute carrier family 35 (UDP-galactose transporter), member A2
1.57	EPHB3	EPH receptor B3
1.57	TPM2	tropomyosin 2
1.57	IGLON5	IgLON family member 5
1.57	P4HA3	prolyl 4-hydroxylase, alpha polypeptide III
1.57	SEC31A	SEC31 homolog A, COPII coat complex component
1.58	YIF1B	Yip1 interacting factor homolog B (S. cerevisiae)
1.58	MARC3	membrane-associated ring finger (C3HC4) 3, E3 ubiquitin protein ligase
1.58	MIR29A	microRNA mir-29a
1.59	LOC102151133	leucine-rich repeat-containing protein 37A3-like; leucine-rich repeat-containing protein 37B-like
1.6	EFCAB6	EF-hand calcium binding domain 6
1.6	NPAS2	neuronal PAS domain protein 2
1.6	HTR7	5-hydroxytryptamine (serotonin) receptor 7, adenylate cyclase-coupled
1.6	SKIL	SKI-like proto-oncogene
1.6	MIR21	microRNA mir-21
1.61	ACAT2	acetyl-CoA acetyltransferase 2
1.61	FNTB	farnesyltransferase, CAAX box, beta
1.61	ENSCAFG00000028490	Chromosome 17: 57,385,006-57,446,097
1.62	MIR107	microRNA mir-107
1.62	ENSCAFG00000002071	Chromosome 10: 36,487,173-36,487,535
1.63	ARMC9	armadillo repeat containing 9
1.63	FBP1	fructose-1,6-bisphosphatase 1
1.63	LOC475563	ubiquitin-conjugating enzyme E2 E1-like
1.63	HHAT	hedgehog acyltransferase
1.63	SHROOM4	shroom family member 4
1.64	BTK	Bruton agammaglobulinemia tyrosine kinase
1.66	TPM2	tropomyosin 2 (beta)
1.67	C1QTNF3	C1q and tumor necrosis factor related protein 3; alpha-methylacyl-CoA racemase
1.67	TMPO	thymopoietin
1.67	SNX29	sorting nexin 29
1.68	VAMP5	vesicle-associated membrane protein 5

1.68	STARD4	StAR related lipid transfer domain containing 4
1.68	GALNT1	polypeptide N-acetylgalactosaminyltransferase 1
1.69	SUGCT	succinyl-CoA:glutarate-CoA transferase
1.7	TMEM86A	transmembrane protein 86A
1.7	SYNPO	synaptopodin
1.7	NONO	non-POU domain containing octamer binding
1.71	SUGCT	succinyl-CoA:glutarate-CoA transferase
1.73	CYP51A1	cytochrome P450, family 51, subfamily A, polypeptide 1
1.74	TSPAN6	tetraspanin 6
1.74	PLAUR	plasminogen activator, urokinase receptor
1.75	LPCAT2	lysophosphatidylcholine acyltransferase 2
1.76	ST5	suppression of tumorigenicity 5
1.77	UACA	uveal autoantigen with coiled-coil domains and ankyrin repeats
1.79	TPM2	tropomyosin 2
1.79	DDIT4	DNA-damage-inducible transcript 4
1.82	MLLT11	myeloid/lymphoid or mixed-lineage leukemia; translocated to, 11
1.83	FAM13A	family with sequence similarity 13, member A
1.84	SUGCT	succinyl-CoA:glutarate-CoA transferase
1.84	TNFSF10	tumor necrosis factor (ligand) superfamily, member 10
1.88	ST5	suppression of tumorigenicity 5
1.88	WFIKKN2	WAP, follistatin/kazal, immunoglobulin, kunitz and netrin domain containing 2
1.88	QPCT	glutaminyl-peptide cyclotransferase
1.88	SERPINE1	serpin peptidase inhibitor, clade E (nexin, plasminogen activator inhibitor type 1), member 1
1.89	PIEZO2	piezo-type mechanosensitive ion channel component 2
1.91	ENSCAFG00000036785	Chromosome 25: 35,570,658-35,745,048
1.95	ACTA2	actin, alpha 2, smooth muscle, aorta
1.96	TM7SF2	transmembrane 7 superfamily member 2
1.96	LIF	leukemia inhibitory factor
2.01	WFDC5	WAP four-disulfide core domain 5
2.07	NEDD9	neural precursor cell expressed, developmentally down-regulated 9
2.1	COL15A1	collagen, type XV, alpha 1
2.1	TAGLN	transgelin
2.1	ADAMTS6	ADAM metallopeptidase with thrombospondin type 1 motif 6

2.1	ADAMTS6	ADAM metallopeptidase with thrombospondin type 1 motif 6
2.1	ADAMTS6	ADAM metallopeptidase with thrombospondin type 1 motif 6
2.13	MFSD2A	major facilitator superfamily domain containing 2A
2.15	ADCY7	adenylate cyclase 7
2.18	EREG	epiregulin
2.23	IGDCC4	immunoglobulin superfamily, DCC subclass, member 4
2.23	WNT5B	wingless-type MMTV integration site family, member 5B
2.26	PMAIP1	phorbol-12-myristate-13-acetate-induced protein 1
2.27	ADAMTS6	ADAM metallopeptidase with thrombospondin type 1 motif, 6
2.28	MEOX1	mesenchyme homeobox 1
2.29	TUBB3	tubulin, beta 3 class III
2.3	LTBP2	latent transforming growth factor beta binding protein 2
2.38	ENSCAFG00000030590	Chromosome 19: 12,626,650-12,627,354
2.39	ANKRD1	ankyrin repeat domain 1 (cardiac muscle)
2.39	ABCA9	ATP-binding cassette, sub-family A (ABC1), member 9
2.41	CADM4	cell adhesion molecule 4
2.41	LRRC6	leucine rich repeat containing 6
2.47	CRABP2	cellular retinoic acid binding protein 2
2.5	PAPPA	pregnancy-associated plasma protein A, pappalysin 1
2.51	FZD2	frizzled class receptor 2
2.53	ST5	suppression of tumorigenicity 5
2.57	APOC1	apolipoprotein C-I
2.61	MIR214	microRNA mir-214
2.68	CH25H	cholesterol 25-hydroxylase
2.85	PMEPA1	prostate transmembrane protein, androgen induced 1
2.99	TMEM71	transmembrane protein 71
3.19	LOC486400	gamma-glutamyltranspeptidase 1
3.2	MIR181B-2	microRNA mir-181b-2
3.28	MFAP4	microfibrillar-associated protein 4
3.55	MIR199-2	microRNA mir-199-2
3.58	AQP11	aquaporin 11
3.71	NOX4	NADPH oxidase 4

3.71	HTR2B	5-hydroxytryptamine (serotonin) receptor 2B, G protein-coupled
3.75	TNC	tenascin C
3.78	TFPI2	tissue factor pathway inhibitor 2
4.15	SRPX2	sushi-repeat containing protein, X-linked 2
4.71	FAP	fibroblast activation protein, alpha
4.71	MSC	musculin
5.81	RGS1	regulator of G-protein signaling 1
6	SNORD93	Small nucleolar RNA SNORD93
