# Supplementary material for: Evaluation of canine 2D cell cultures as models of myxomatous mitral valve degeneration
Source: PLoS One. 2019 Aug 15;14(8):e0221126. doi: 10.1371/journal.pone.0221126 (PMC6695117; doi:10.1371/journal.pone.0221126)
Supplement: S5 Table — (PDF) [file pone.0221126.s005.pdf]

S5 Table. Gene list SB431542 treated aVICs vs aVICs with fold change < or > 1.5 (236 differentially expressed genes; 115 down, 121 up)

Fold Change	Gene Symbol	Description
-7.83	CRABP2	cellular retinoic acid binding protein 2
-6.94	FAP	fibroblast activation protein, alpha
-6.86	SRPX2	sushi-repeat containing protein, X-linked 2
-6.36	ACTA2	actin, alpha 2, smooth muscle, aorta
-5.65	PRG4	proteoglycan 4
-5.34	LOC487977	cell surface glycoprotein CD200 receptor 1
-5.16	HTR2B	5-hydroxytryptamine (serotonin) receptor 2B, G protein-coupled
-4.56	PI16	peptidase inhibitor 16
-4.5	CMKLR1	chemerin chemokine-like receptor 1
-4.38	FAM26E	family with sequence similarity 26, member E
-4.16	SERTAD4	SERTA domain containing 4
-4.14	MXRA5	matrix-remodelling associated 5
-4.07	PMEPA1	prostate transmembrane protein, androgen induced 1
-3.99	TMEM100	transmembrane protein 100
-3.76	TXNIP	thioredoxin interacting protein
-3.71	COL12A1	collagen, type XII, alpha 1
-3.7	COL1A1	collagen, type I, alpha 1
-3.6	LOC479376	cadherin-6
-3.05	LOC486400	gamma-glutamyltranspeptidase 1
-2.92	SLC8A1	solute carrier family 8 (sodium/calcium exchanger), member 1
-2.8	SGCD	sarcoglycan, delta (35kDa dystrophin-associated glycoprotein)
-2.77	ADCY7	adenylate cyclase 7
-2.63	LMCD1	LIM and cysteine-rich domains 1
-2.6	GPER1	G protein-coupled estrogen receptor 1
-2.58	TAGLN	transgelin
-2.56	SKIL	SKI-like proto-oncogene
-2.53	ITGA1	integrin, alpha 1
-2.5	EPHB3	EPH receptor B3
-2.48	TGFB3	transforming growth factor, beta 3
-2.47	COL3A1	collagen type III alpha 1 chain
-2.47	MEOX1	mesenchyme homeobox 1
-2.45	PAPPA	pregnancy-associated plasma protein A, pappalysin 1

-2.38	ARRDC4	arrestin domain containing 4
-2.38	FZD2	frizzled class receptor 2
-2.37	ANKRD1	ankyrin repeat domain 1 (cardiac muscle)
-2.33	TNFSF10	tumor necrosis factor (ligand) superfamily, member 10
-2.3	UACA	uveal autoantigen with coiled-coil domains and ankyrin repeats
-2.24	MIR214	microRNA mir-214
-2.19	BAMBI	BMP and activin membrane-bound inhibitor
-2.17	GJA1	gap junction protein, alpha 1, 43kDa
-2.16	SEMA3C	sema domain, immunoglobulin domain (Ig), short basic domain, secreted, (semaphorin) 3C
-2.15	DKK1	dickkopf WNT signaling pathway inhibitor 1
-2.09	HHAT	hedgehog acyltransferase
-2.07	ITPR1	inositol 1,4,5-trisphosphate receptor, type 1
-2.07	MRC2	mannose receptor, C type 2
-2.07	PTPRN	protein tyrosine phosphatase, receptor type, N
-2.04	RCOR2	REST corepressor 2
-2.03	COL3A1	collagen, type III, alpha 1
-2.03	CNN1	calponin 1, basic, smooth muscle
-2	JADE1	jade family PHD finger 1
-2	TRABD2B	TraB domain containing 2B
-2	MIR665	microRNA mir-665
-1.99	NUAK1	NUAK family, SNF1-like kinase, 1
-1.94	WNT5B	wingless-type MMTV integration site family, member 5B
-1.93	MIR27B	microRNA mir-27b
-1.93	NOX4	NADPH oxidase 4
-1.9	RASL11B	RAS-like, family 11, member B
-1.89	LOC476300	fructose-1,6-bisphosphatase isozyme 2
-1.89	SMTN	smoothelin
-1.88	EBF4	early B-cell factor 4
-1.86	CTNNAL1	catenin (cadherin-associated protein), alpha-like 1
-1.86	FAM20A	family with sequence similarity 20, member A
-1.85	GAL3ST4	galactose-3-O-sulfotransferase 4
-1.84	ARMC9	armadillo repeat containing 9
-1.79	MYL9	myosin, light chain 9, regulatory
-1.78	TRABD2B	TraB domain containing 2B
-1.74	WFIKKN2	WAP, follistatin/kazal, immunoglobulin, kunitz and netrin domain containing 2

-1.74	ENSCAFG00000025740	ENSCAFG00000025740
-1.74	ENSCAFG00000025740	ENSCAFG00000025740
-1.74	ENSCAFG00000025740	ENSCAFG00000025740
-1.73	DSEL	dermatan sulfate epimerase-like
-1.73	KCND2	potassium channel, voltage gated Shal related subfamily D, member 2
-1.73	TNFRSF25	tumor necrosis factor receptor superfamily, member 25
-1.7	MLLT11	myeloid/lymphoid or mixed-lineage leukemia; translocated to, 11
-1.7	DPT	dermatopontin
-1.69	BCL2L11	BCL2-like 11 (apoptosis facilitator)
-1.68	FAM46A	family with sequence similarity 46, member A
-1.68	FKBP1B	FK506 binding protein 1B, 12.6 kDa
-1.68	TBC1D1	TBC1 (tre-2/USP6, BUB2, cdc16) domain family, member 1
-1.68	MIR199-2	microRNA mir-199-2
-1.67	KANK1	KN motif and ankyrin repeat domains 1
-1.67	MICAL2	microtubule associated monooxygenase, calponin and LIM domain containing 2
-1.66	COL5A1	collagen, type V, alpha 1
-1.65	ARL9	ADP-ribosylation factor-like 9
-1.65	COL11A1	collagen, type XI, alpha 1
-1.64	SYNPO	synaptopodin
-1.63	CHSY1	chondroitin sulfate synthase 1
-1.63	ZFYVE28	zinc finger FYVE-type containing 28
-1.63	TTC14	tetratricopeptide repeat domain 14
-1.6	CD200	CD200 molecule
-1.6	EFNA4	ephrin-A4
-1.59	FNBP1L	formin binding protein 1-like
-1.59	ENPP1	ectonucleotide pyrophosphatase/phosphodiesterase 1
-1.58	DAB2	Dab, mitogen-responsive phosphoprotein, homolog 2 (Drosophila)
-1.58	THY1	Thy-1 cell surface antigen
-1.58	PLEKHG5	pleckstrin homology domain containing, family G (with RhoGef domain) member 5
-1.57	PLOD2	procollagen-lysine, 2-oxoglutarate 5-dioxygenase 2
-1.57	AMOTL2	angiomotin like 2
-1.57	TBC1D1	TBC1 (tre-2/USP6, BUB2, cdc16) domain family, member 1
-1.56	WNK4	WNK lysine deficient protein kinase 4; vacuolar protein sorting 25 homolog (S. cerevisiae)

-1.56	PXDC1	PX domain containing 1
-1.56	FKBP14	FK506 binding protein 14, 22 kDa
-1.56	LOXL1	lysyl oxidase-like 1
-1.56	PHEX	phosphate regulating endopeptidase homolog, X-linked
-1.55	HHIPL1	HHIP-like 1
-1.55	LHX9	LIM homeobox 9
-1.54	ZNF827	zinc finger protein 827
-1.52	LOC487174	protocadherin beta-7
-1.52	DNAJB6	DnaJ heat shock protein family (Hsp40) member B6
-1.52	SHROOM4	shroom family member 4
-1.52	ENSCAFG00000023482	ENSCAFG00000023482
-1.51	STAC	SH3 and cysteine rich domain
-1.51	COL5A2	collagen, type V, alpha 2
-1.51	DCHS1	dachsous cadherin-related 1
-1.51	TPM1	tropomyosin 1 (alpha)
1.51	CERS4	ceramide synthase 4
1.51	ANKRD28	ankyrin repeat domain 28
1.51	RORA	RAR-related orphan receptor A
1.51	PLCD4	phospholipase C, delta 4
1.51	DUSP3	dual specificity phosphatase 3
1.52	HCAR1	hydroxycarboxylic acid receptor 1
1.52	LACTB2	lactamase, beta 2
1.53	LOC106557488	neurexin-3-beta
1.53	PXMP4	peroxisomal membrane protein 4, 24kDa
1.53	SLC25A33	solute carrier family 25 (pyrimidine nucleotide carrier), member 33
1.53	EPAS1	endothelial PAS domain protein 1
1.53	ZHX2	zinc fingers and homeoboxes 2
1.53	CCDC115	coiled-coil domain containing 115
1.53	GPR137B	G protein-coupled receptor 137B
1.53	SLC16A5	solute carrier family 16 (monocarboxylate transporter), member 5
1.54	FDFT1	farnesyl-diphosphate farnesyltransferase 1
1.54	SERAC1	serine active site containing 1
1.54	DUSP5	dual specificity phosphatase 5
1.54	MTMR4	myotubularin related protein 4
1.54	LOC490881	protein AHNAK2
1.54	ARMCX1	armadillo repeat containing, X-linked 1

1.55	CAECAM1	carcinoembryonic antigen-related cell adhesion molecule 25
1.55	GCHFR	GTP cyclohydrolase I feedback regulator
1.55	LOC491253	uncharacterized LOC491253
1.57	SPTLC2	serine palmitoyltransferase, long chain base subunit 2
1.58	MGAT4B	mannosyl (alpha-1,3-)-glycoprotein beta-1,4-N-acetylglucosaminyltransferase, isozyme B
1.58	SQSTM1	sequestosome 1
1.58	PIGW	phosphatidylinositol glycan anchor biosynthesis, class W
1.59	FASN	fatty acid synthase
1.59	RRAGC	Ras-related GTP binding C
1.59	SH2D4A	SH2 domain containing 4A
1.59	LOC611145	proline-rich protein 23A-like
1.6	MID1IP1	MID1 interacting protein 1
1.6	EGFL8	EGF-like-domain, multiple 8
1.6	MIR8810	microRNA mir-8810; adenosine monophosphate deaminase 3
1.6	RCBTB1	regulator of chromosome condensation (RCC1) and BTB (POZ) domain containing protein 1
1.61	HS1BP3	HCLS1 binding protein 3
1.62	CKB	Creatine kinase B-type
1.62	EML2	echinoderm microtubule associated protein like 2
1.62	PKNOX1	PBX/knotted 1 homeobox 1
1.64	PDE7B	phosphodiesterase 7B
1.65	HMX1	H6 family homeobox 1
1.65	PCYT2	phosphate cytidylyltransferase 2, ethanolamine
1.65	NPC2	Niemann-Pick disease, type C2
1.66	FNIP2	folliculin interacting protein 2
1.66	FUOM	fucose mutarotase
1.67	SDC4	syndecan 4
1.67	LOC100856505	melanoma-associated antigen 8-like
1.69	HERC6	HECT and RLD domain containing E3 ubiquitin protein ligase family member 6
1.69	SLC37A2	solute carrier family 37 (glucose-6-phosphate transporter), member 2
1.7	IRF1	interferon regulatory factor 1
1.71	CPAMD8	C3 and PZP-like, alpha-2-macroglobulin domain containing 8
1.73	ACAT2	acetyl-CoA acetyltransferase 2
1.74	MVK	mevalonate kinase

1.74	RNF144B	ring finger protein 144B
1.74	FLCN	folliculin
1.75	DOK5	docking protein 5
1.75	PDK2	pyruvate dehydrogenase kinase, isozyme 2
1.77	MTSS1	metastasis suppressor 1
1.77	BRI3	brain protein I3
1.77	CLIC3	chloride intracellular channel 3
1.78	MAGI2	membrane associated guanylate kinase, WW and PDZ domain containing 2
1.78	AHNAK2	AHNAK nucleoprotein 2
1.78	LOC609831	signal-regulatory protein beta-1 isoform 3-like
1.8	LDLR	low density lipoprotein receptor
1.81	KIAA1456	KIAA1456 ortholog
1.82	TIMP3	TIMP metallopeptidase inhibitor 3
1.83	NDNF	neuron-derived neurotrophic factor
1.83	GAP43	growth associated protein 43
1.85	HECW1	HECT, C2 and WW domain containing E3 ubiquitin protein ligase 1
1.85	CCBL2	cysteine conjugate-beta lyase 2
1.85	TMOD1	tropomodulin 1
1.86	LOC481227	neuronal-specific septin-3
1.87	OCA2	oculocutaneous albinism II
1.91	SC5D	sterol-C5-desaturase
1.93	SNX8	sorting nexin 8
1.94	DEPDC7	DEP domain containing 7
1.94	PCBD1	pterin-4 alpha-carbinolamine dehydratase/dimerization cofactor of hepatocyte nuclear factor 1 alpha
1.96	MEF2C	myocyte enhancer factor 2C
1.96	MT2A	metallothionein 1H
1.97	TM7SF2	transmembrane 7 superfamily member 2
1.98	TRPM6	transient receptor potential cation channel, subfamily M, member 6
1.98	EPHB1	EPH receptor B1
2	TNFRSF8	TNF receptor superfamily member 8
2.01	EPHB1	EPH receptor B1
2.03	TMEM140	transmembrane protein 140
2.03	AHNAK2	AHNAK nucleoprotein 2
2.03	RNF213	ring finger protein 213
2.05	SVIP	small VCP/p97-interacting protein

2.08	AMDHD2	amidohydrolase domain containing 2
2.09	CNKSR3	CNKSR family member 3
2.1	HBEGF	heparin-binding EGF-like growth factor
2.1	DHRS13	dehydrogenase/reductase (SDR family) member 13
2.16	BCL2A1	BCL2-related protein A1
2.16	UCP2	uncoupling protein 2 (mitochondrial, proton carrier)
2.17	LOC476208	putative PDZ domain-containing protein PDZK1P1
2.18	ELOVL7	ELOVL fatty acid elongase 7
2.2	PIK3CB	phosphatidylinositol-4,5-bisphosphate 3-kinase, catalytic subunit beta
2.21	ZDHHC14	zinc finger, DHHC-type containing 14
2.22	NHS	Nance-Horan syndrome (congenital cataracts and dental anomalies)
2.28	AACS	acetoacetyl-CoA synthetase; BRI3 binding protein
2.29	LMO2	LIM domain only 2 (rhombotin-like 1)
2.29	CYP24A1	cytochrome P450, family 24, subfamily A, polypeptide 1
2.29	LOC100855783	kinesin-like protein KIF1A
2.3	PHACTR1	phosphatase and actin regulator 1
2.4	GPNMB	glycoprotein (transmembrane) nmb
2.4	INSIG1	insulin induced gene 1
2.45	EBP	emopamil binding protein (sterol isomerase)
2.48	LGALSL	lectin, galactoside-binding-like
2.48	MAFB	v-maf avian musculoaponeurotic fibrosarcoma oncogene homolog B
2.5	CCDC113	coiled-coil domain containing 113
2.78	GPM6A	glycoprotein M6A
2.85	LOC100686073	metallothionein-1
2.92	CRYM	crystallin, mu
3.02	SNN	stannin
3.07	LOC491239	UDP-N-acetylhexosamine pyrophosphorylase-like protein 1
3.08	LOC606974	UDP-N-acetylhexosamine pyrophosphorylase-like protein 1
3.39	ZDHHC14	zinc finger, DHHC-type containing 14
4.88	RRAGD	Ras-related GTP binding D
5.83	NDP	Norrie disease (pseudoglioma)
8.07	CASP14	caspase 14, apoptosis-related cysteine peptidase
