# Supplementary material for: Evaluation of canine 2D cell cultures as models of myxomatous mitral valve degeneration
Source: PLoS One. 2019 Aug 15;14(8):e0221126. doi: 10.1371/journal.pone.0221126 (PMC6695117; doi:10.1371/journal.pone.0221126)
Supplement: S6 Table — (PDF) [file pone.0221126.s006.pdf]

S6 Table. Gene list TGFβ1-treated qVICs vs aVICs with fold change < or > 1.5 (832 differentially expressed genes; 490 down, 342 up)

Fold Change	Gene Symbol	Description
-26.46	ACTG2	actin, gamma 2, smooth muscle, enteric
-13.23	ITGA8	integrin, alpha 8
-11.3	EPHA3	EPH receptor A3
-9.88	DKK2	dickkopf WNT signaling pathway inhibitor 2
-8.89	NEK2	NIMA-related kinase 2
-8.39	MIR1840	microRNA mir-1840
-8.3	RRM2	ribonucleotide reductase M2
-8.08	ESCO2	establishment of sister chromatid cohesion N-acetyltransferase 2
-7.91	PBK	PDZ binding kinase
-7.46	THBS2	thrombospondin 2
-6.93	NUF2	NUF2, NDC80 kinetochore complex component
-6.88	PTTG1	pituitary tumor-transforming 1
-6.63	KIF20A	kinesin family member 20A
-6.52	HJURP	Holliday junction recognition protein
-6.27	LOC102152056	antigen KI-67-like; antigen identified by monoclonal antibody Ki-67
-6.15	RNF144A	ring finger protein 144A
-6.07	CDCA3	cell division cycle associated 3
-5.95	CDKN3	cyclin-dependent kinase inhibitor 3
-5.86	SHCBP1	SHC SH2-domain binding protein 1
-5.78	UBE2C	ubiquitin-conjugating enzyme E2C
-5.73	DIAPH3	diaphanous-related formin 3
-5.69	GNG11	guanine nucleotide binding protein (G protein), gamma 11
-5.65	DLGAP5	discs, large (Drosophila) homolog-associated protein 5
-5.64	BUB1	BUB1 mitotic checkpoint serine/threonine kinase
-5.59	CCNB1	cyclin B1
-5.55	ASPM	abnormal spindle microtubule assembly
-5.52	LOC491373	uncharacterized LOC491373
-5.48	IDNK	idnK, gluconokinase homolog (E. coli)
-5.26	PRC1	protein regulator of cytokinesis 1
-5.12	CNN1	calponin 1, basic, smooth muscle
-5.09	MDGA2	MAM domain containing glycosylphosphatidylinositol anchor 2
-4.92	ASF1B	anti-silencing function 1B histone chaperone
-4.81	ECT2	epithelial cell transforming 2
-4.78	TOP2A	topoisomerase (DNA) II alpha
-4.74	CCNA2	cyclin A2
-4.74	ACPP	acid phosphatase, prostate

-4.72	NCAPG	non-SMC condensin I complex, subunit G
-4.72	KIF4A	kinesin family member 4A
-4.71	DIAPH3	diaphanous-related formin 3
-4.71	DEPDC1	DEP domain containing 1
-4.71	KIAA0101	KIAA0101 ortholog; casein kinase 1, gamma 1
-4.7	E2F8	E2F transcription factor 8
-4.68	SPC25	SPC25, NDC80 kinetochore complex component
-4.65	PRR11	proline rich 11
-4.61	KIF11	kinesin family member 11
-4.53	LOC102153443	rho GTPase-activating protein 20-like
-4.53	CIT	citron rho-interacting serine/threonine kinase
-4.51	CENPF	centromere protein F, 350/400kDa
-4.48	CKAP2L	cytoskeleton associated protein 2-like
-4.48	KIF23	kinesin family member 23
-4.46	CASC5	cancer susceptibility candidate 5
-4.43	CDC20	cell division cycle 20
-4.42	NEDD9	neural precursor cell expressed, developmentally down-regulated 9
-4.4	BUB1B	BUB1 mitotic checkpoint serine/threonine kinase B
-4.39	LOC489372	histone H2AX
-4.32	TROAP	trophinin associated protein
-4.3	GTSE1	G-2 and S-phase expressed 1
-4.23	NDC80	NDC80 kinetochore complex component
-4.22	FGD5	FYVE, RhoGEF and PH domain containing 5
-4.18	MYBL2	v-myb avian myeloblastosis viral oncogene homolog-like 2
-4.14	SKA3	spindle and kinetochore associated complex subunit 3
-4.09	FOXM1	forkhead box M1
-4.09	TTK	TTK protein kinase
-4.07	CENPT	centromere protein T
-4.04	ANLN	anillin actin binding protein
-4.03	PLK1	polo-like kinase 1
-4.03	RND3	Rho family GTPase 3
-3.98	CENPU	centromere protein U
-3.95	LOC102151762	uncharacterized LOC102151762
-3.95	ACTA2	actin, alpha 2, smooth muscle, aorta
-3.94	ALDH1A3	aldehyde dehydrogenase 1 family, member A3
-3.92	FAM64A	family with sequence similarity 64, member A
-3.91	USP1	ubiquitin specific peptidase 1
-3.89	SPC24	SPC24, NDC80 kinetochore complex component
-3.88	TPX2	TPX2, microtubule-associated
-3.84	CLSPN	claspin
-3.83	RAB38	RAB38, member RAS oncogene family
-3.83	PLK4	polo-like kinase 4

-3.81	MIS18A	MIS18 kinetochore protein A
-3.77	UBE2T	ubiquitin-conjugating enzyme E2T
-3.77	LOC102152154	rho GTPase-activating protein 20-like; uncharacterized LOC102151972
-3.77	ARSE	arylsulfatase E (chondrodysplasia punctata 1)
-3.76	CDCA8	cell division cycle associated 8
-3.75	RACGAP1	Rac GTPase activating protein 1
-3.73	SASS6	SAS-6 centriolar assembly protein
-3.73	PEG3	paternally expressed 3
-3.72	UHRF1	ubiquitin-like with PHD and ring finger domains 1
-3.72	CCNB3	cyclin B3
-3.7	KIFC1	kinesin family member C1
-3.69	MCM5	minichromosome maintenance complex component 5
-3.67	RAD51	RAD51 recombinase
-3.66	ARAP2	ArfGAP with RhoGAP domain, ankyrin repeat and PH domain 2
-3.63	EFNA5	ephrin-A5
-3.61	BIRC5	baculoviral IAP repeat containing 5
-3.6	ITGBL1	integrin, beta-like 1 (with EGF-like repeat domains)
-3.59	LOC100855995	tubulin alpha chain-like
-3.59	MAB21L2	mab-21-like 2 (C. elegans)
-3.57	FANCD2	Fanconi anemia, complementation group D2
-3.56	TK1	thymidine kinase 1, soluble
-3.54	CDK1	cyclin-dependent kinase 1
-3.53	ARHGAP11A	Rho GTPase activating protein 11A
-3.52	LOC102153827	uncharacterized LOC102153827
-3.52	PI16	peptidase inhibitor 16
-3.49	KIF15	kinesin family member 15
-3.48	CDCA2	cell division cycle associated 2
-3.45	CCNB2	cyclin B2
-3.44	TACC3	transforming, acidic coiled-coil containing protein 3; transforming acidic coiled-coil-containing protein 3-like
-3.42	PTGFR	prostaglandin F receptor (FP)
-3.38	ENPP5	ectonucleotide pyrophosphatase/phosphodiesterase 5 (putative)
-3.37	MELK	maternal embryonic leucine zipper kinase
-3.36	FAM20A	family with sequence similarity 20, member A
-3.33	TMEM88	transmembrane protein 88
-3.31	NCAPH	non-SMC condensin I complex, subunit H
-3.29	E2F1	E2F transcription factor 1
-3.27	KNSTRN	kinetochore-localized astrin/SPAG5 binding protein
-3.25	KNTC1	kinetochore associated 1
-3.24	CCNF	cyclin F

-3.24	MCM3	minichromosome maintenance complex component 3
-3.24	KIF18B	kinesin family member 18B
-3.24	GPRC5A	G protein-coupled receptor class C group 5 member A
-3.22	KIF20B	kinesin family member 20B
-3.17	RMI2	RecQ mediated genome instability 2
-3.15	CYP26B1	cytochrome P450, family 26, subfamily B, polypeptide 1
-3.15	CDC45	cell division cycle 45
-3.12	CENPE	centromere protein E, 312kDa
-3.11	CENPA	centromere protein A
-3.11	CCDC150	coiled-coil domain containing 150
-3.1	POLA2	polymerase (DNA directed), alpha 2, accessory subunit
-3.08	SPAG5	sperm associated antigen 5
-3.08	KIF2C	kinesin family member 2C
-3.07	CEP55	centrosomal protein 55kDa
-3.01	FANCI	Fanconi anemia, complementation group I
-3	SULF2	sulfatase 2
-3	ORC1	origin recognition complex, subunit 1
-3	SLC8A1	solute carrier family 8 (sodium/calcium exchanger), member 1
-2.99	TP53I11	tumor protein p53 inducible protein 11
-2.98	HOXD8	homeobox D8
-2.94	PKMYT1	protein kinase, membrane associated tyrosine/threonine 1
-2.93	MCM10	minichromosome maintenance 10 replication initiation factor
-2.92	SLC1A1	solute carrier family 1 (neuronal/epithelial high affinity glutamate transporter, system Xag), member 1
-2.88	CHAF1B	chromatin assembly factor 1, subunit B (p60)
-2.86	SGOL1	shugoshin-like 1 (S. pombe)
-2.84	FAM81A	family with sequence similarity 81, member A
-2.81	ZFHX4	zinc finger homeobox 4
-2.8	SLC7A2	solute carrier family 7 (cationic amino acid transporter, y+ system), member 2
-2.8	HMMR	hyaluronan-mediated motility receptor (RHAMM)
-2.8	POLE	polymerase (DNA directed), epsilon, catalytic subunit
-2.78	NGEF	neuronal guanine nucleotide exchange factor
-2.77	NME1	non-metastatic cells 1, protein (NM23A) expressed in
-2.75	MCM2	minichromosome maintenance complex component 2
-2.75	LOC100856294	rho GTPase-activating protein 20-like

-2.75	KIF22	kinesin family member 22
-2.73	IQGAP3	IQ motif containing GTPase activating protein 3
-2.72	LOC489024	core histone macro-H2A.2
-2.7	GAS2L3	growth arrest-specific 2 like 3
-2.7	DTL	denticleless E3 ubiquitin protein ligase homolog (Drosophila)
-2.68	ESPL1	extra spindle pole bodies like 1, separase
-2.67	ARHGEF39	Rho guanine nucleotide exchange factor (GEF) 39
-2.66	RAD51AP1	RAD51 associated protein 1
-2.65	LOC488258	histone H1.1
-2.65	RAVER2	ribonucleoprotein, PTB-binding 2
-2.65	LOC486670	histone H4
-2.64	LIMS2	LIM and senescent cell antigen-like domains 2
-2.62	PARPBP	PARP1 binding protein
-2.62	C9H17orf53	chromosome 9 open reading frame, human C17orf53
-2.61	CENPM	centromere protein M
-2.61	REEP4	receptor accessory protein 4
-2.6	CDC6	cell division cycle 6
-2.57	RAD54L	RAD54-like (S. cerevisiae)
-2.56	FAM72A	family with sequence similarity 72, member A
-2.55	GINS4	GINS complex subunit 4 (Sld5 homolog)
-2.54	AURKB	aurora kinase B
-2.54	GPER1	G protein-coupled estrogen receptor 1
-2.53	SP100	SP100 nuclear antigen
-2.52	RFC4	replication factor C (activator 1) 4, 37kDa
-2.51	ACSL5	acyl-CoA synthetase long-chain family member 5
-2.49	MEF2C	myocyte enhancer factor 2C
-2.49	HMGB2	high mobility group box 2
-2.48	RFC3	replication factor C (activator 1) 3, 38kDa
-2.47	CENPI	centromere protein I
-2.46	FABP3	fatty acid binding protein 3, muscle and heart
-2.46	TRIP13	thyroid hormone receptor interactor 13
-2.46	MYH10	myosin, heavy chain 10, non-muscle
-2.45	OIP5	Opa interacting protein 5
-2.45	BRCA1	breast cancer 1, early onset
-2.43	C4H10orf54	chromosome 4 open reading frame, human C10orf54
-2.43	FAM160A1	family with sequence similarity 160 member A1
-2.42	LAMA2	laminin, alpha 2
-2.41	EXO1	exonuclease 1
-2.41	ADAMTS12	ADAM metallopeptidase with thrombospondin type 1 motif, 12
-2.4	SMC2	structural maintenance of chromosomes 2
-2.39	PAK1	p21 protein (Cdc42/Rac)-activated kinase 1

-2.37	CDKN2C	cyclin-dependent kinase inhibitor 2C (p18, inhibits CDK4)
-2.37	KIF18A	kinesin family member 18A
-2.36	KIF14	kinesin family member 14
-2.35	NDP	Norrie disease (pseudoglioma)
-2.35	CENPO	centromere protein O
-2.34	ARHGAP6	Rho GTPase activating protein 6
-2.33	C20H3orf14	chromosome 20 open reading frame, human C3orf14
-2.33	LOC102153827	uncharacterized LOC102153827
-2.33	FANCA	Fanconi anemia, complementation group A
-2.32	AKAP12	A kinase (PRKA) anchor protein 12
-2.32	MYLK	myosin light chain kinase
-2.29	FAM83D	family with sequence similarity 83, member D
-2.28	PDE3B	phosphodiesterase 3B, cGMP-inhibited
-2.28	GPR143	G protein-coupled receptor 143
-2.28	MASTL	microtubule associated serine/threonine kinase-like
-2.28	HELLS	helicase, lymphoid-specific
-2.27	NCAPD2	non-SMC condensin I complex, subunit D2
-2.26	GPC4	glypican 4
-2.26	NUSAP1	nucleolar and spindle associated protein 1
-2.26	CDT1	chromatin licensing and DNA replication factor 1
-2.25	FAM198B	family with sequence similarity 198, member B
-2.24	TLN2	talin 2
-2.22	TCF19	transcription factor 19
-2.22	WDR62	WD repeat domain 62
-2.21	KIAA1524	KIAA1524 ortholog
-2.21	SNORD26	Small nucleolar RNA SNORD26
-2.18	PRIM1	primase, DNA, polypeptide 1 (49kDa)
-2.17	TCF21	transcription factor 21
-2.17	VWA5A	von Willebrand factor A domain containing 5A
-2.17	SEMA5A	sema domain, seven thrombospondin repeats (type 1 and type 1-like), transmembrane domain (TM) and short cytoplasmic domain, (semaphorin) 5A
-2.17	CPXM2	carboxypeptidase X (M14 family), member 2
-2.17	MIR6516	microRNA mir-6516
-2.16	AKAP2	A kinase (PRKA) anchor protein 2
-2.15	CRYL1	crystallin, lambda 1
-2.15	E2F7	E2F transcription factor 7
-2.12	POC1A	POC1 centriolar protein A
-2.12	MLLT3	myeloid/lymphoid or mixed-lineage leukemia; translocated to, 3
-2.11	BLM	Bloom syndrome, RecQ helicase-like
-2.11	NDUFS8	NADH dehydrogenase (ubiquinone) Fe-S protein 8, 23kDa (NADH-coenzyme Q reductase)

-2.11	DDIAS	DNA damage-induced apoptosis suppressor
-2.11	BRCA2	breast cancer 2, early onset
-2.11	SULF1	sulfatase 1
-2.11	UNC5B	unc-5 netrin receptor B
-2.1	MIS18BP1	MIS18 binding protein 1
-2.09	RPA2	replication protein A2, 32kDa
-2.08	MTUS1	microtubule associated scaffold protein 1
-2.07	APOL5	apolipoprotein L5
-2.07	CENPN	centromere protein N
-2.07	LOC479820	exonuclease NEF-sp
-2.06	CTH	cystathionine gamma-lyase
-2.06	CHAF1A	chromatin assembly factor 1, subunit A (p150)
-2.04	KIF24	kinesin family member 24
-2.03	GCAT	glycine C-acetyltransferase
-2.03	TRAIP	TRAF interacting protein
-2.03	LAMA2	laminin, alpha 2
-2.03	NUDT1	nudix (nucleoside diphosphate linked moiety X)-type motif 1
-2.02	ZWILCH	zwilch kinetochore protein
-2.01	DOCK3	dedicator of cytokinesis 3
-2.01	ENPP4	ectonucleotide pyrophosphatase/phosphodiesterase 4 (putative)
-2.01	STBD1	starch binding domain 1
-2.01	PHF19	PHD finger protein 19
-2.01	GPRC5A	G protein-coupled receptor, class C, group 5, member A
-2	SUV39H1	suppressor of variegation 3-9 homolog 1 (Drosophila)
-2	MYL9	myosin, light chain 9, regulatory
-1.99	RFC5	replication factor C (activator 1) 5, 36.5kDa
-1.98	SNRPA1	small nuclear ribonucleoprotein polypeptide A
-1.98	FKBP4	FK506 binding protein 4, 59kDa
-1.98	CCNE2	cyclin E2
-1.98	CENPH	centromere protein H
-1.96	RPS29	ribosomal protein S29
-1.95	KIAA1456	KIAA1456 ortholog
-1.95	CKB	creatine kinase, brain
-1.95	DHTKD1	dehydrogenase E1 and transketolase domain containing 1
-1.94	ANKLE1	ankyrin repeat and LEM domain containing 1
-1.94	LOC488260	uncharacterized LOC488260
-1.94	RTKN2	rhotekin 2
-1.93	SNRPA	small nuclear ribonucleoprotein polypeptide A
-1.93	SMC4	structural maintenance of chromosomes 4
-1.92	CKB	Creatine kinase B-type

-1.92	LOC488263	histone H3.1
-1.92	LOC488277	histone H4
-1.91	ADGRE5	adhesion G protein-coupled receptor E5
-1.91	SCAPER	S-phase cyclin A associated protein in the ER
-1.91	EGLN1	egl-9 family hypoxia inducible factor 1
-1.9	GEN1	GEN1 Holliday junction 5 flap endonuclease
-1.89	ELOVL6	ELOVL fatty acid elongase 6
-1.89	RRM1	ribonucleotide reductase M1
-1.88	NGFR	nerve growth factor receptor
-1.88	PSRC1	proline/serine-rich coiled-coil 1
-1.88	TIAM1	T-cell lymphoma invasion and metastasis 1
-1.88	ERI3	ERI1 exoribonuclease family member 3
-1.88	SRGN	serglycin
-1.87	RALGPS2	Ral GEF with PH domain and SH3 binding motif 2
-1.86	EMILIN2	elastin microfibril interfacer 2; structural maintenance of chromosomes flexible hinge domain containing 1
-1.86	DTD2	D-tyrosyl-tRNA deacylase 2 (putative)
-1.86	SETBP1	SET binding protein 1
-1.86	GALNT18	polypeptide N-acetylgalactosaminyltransferase 18
-1.86	SQRDL	sulfide quinone reductase-like (yeast)
-1.85	FOSL1	FOS-like antigen 1
-1.85	TPM2	tropomyosin 2 (beta)
-1.85	CEP152	centrosomal protein 152kDa
-1.84	TSEN2	TSEN2 tRNA splicing endonuclease subunit
-1.84	LOC100856570	trifunctional purine biosynthetic protein adenosine-3; phosphoribosylglycinamide formyltransferase, phosphoribosylglycinamide synthetase, phosphoribosylaminoimidazole synthetase
-1.84	MCM4	minichromosome maintenance complex component 4
-1.84	PFDN1	Prefoldin subunit 1
-1.84	GALNT15	polypeptide N-acetylgalactosaminyltransferase 15
-1.84	LOC488297	histone H2A type 1
-1.84	ACOT7	acyl-CoA thioesterase 7
-1.84	LHX9	LIM homeobox 9
-1.83	MEST	mesoderm specific transcript
-1.83	KALRN	kalirin, RhoGEF kinase
-1.83	RARRES1	retinoic acid receptor responder 1
-1.82	FAM110B	family with sequence similarity 110, member B
-1.82	LOC483167	histone H3-like
-1.81	IL15RA	interleukin 15 receptor, alpha
-1.81	FXN	frataxin
-1.81	GPR63	G protein-coupled receptor 63
-1.81	TRIB1	tribbles pseudokinase 1

-1.8	BRI3BP	BRI3 binding protein
-1.8	SCARB1	scavenger receptor class B, member 1
-1.8	TPMT	thiopurine S-methyltransferase
-1.8	GSG2	germ cell associated 2 (haspin)
-1.8	PLA2R1	phospholipase A2 receptor 1, 180kDa
-1.79	ABCC1	ATP-binding cassette, sub-family C (CFTR/MRP), member 1
-1.79	CHTF18	chromosome transmission fidelity factor 18
-1.79	NOP56	NOP56 ribonucleoprotein
-1.79	SP100	SP100 nuclear antigen
-1.79	DCLRE1B	DNA cross-link repair 1B
-1.79	CENPJ	centromere protein J
-1.78	SIX2	SIX homeobox 2
-1.78	PANX1	pannexin 1
-1.78	ZRANB3	zinc finger RANBP2-type containing 3
-1.78	POLA1	polymerase (DNA directed), alpha 1, catalytic subunit
-1.78	ORC6	origin recognition complex, subunit 6
-1.78	WDR76	WD repeat domain 76
-1.78	FANCM	Fanconi anemia, complementation group M
-1.77	VEGFC	vascular endothelial growth factor C
-1.77	SLC13A5	solute carrier family 13 (sodium-dependent citrate transporter), member 5
-1.76	RHOD	ras homolog family member D
-1.75	SAMHD1	SAM domain and HD domain 1
-1.75	FEN1	flap structure-specific endonuclease 1
-1.75	MSH2	mutS homolog 2
-1.75	DNPH1	2-deoxynucleoside 5-phosphate N-hydrolase 1
-1.75	MARK1	MAP/microtubule affinity-regulating kinase 1
-1.74	S100A4	S100 calcium binding protein A4
-1.74	MTFR2	mitochondrial fission regulator 2
-1.74	C7H1orf112	chromosome 7 open reading frame, human C1orf112
-1.73	VIPR1	vasoactive intestinal peptide receptor 1
-1.73	DBI	diazepam binding inhibitor (GABA receptor modulator, acyl-CoA binding protein)
-1.73	FAM206A	family with sequence similarity 206, member A
-1.73	EMG1	EMG1 N1-specific pseudouridine methyltransferase
-1.73	HMGB3	high mobility group box 3
-1.73	FZD1	frizzled class receptor 1
-1.73	H1FX	H1 histone family, member X
-1.73	ARHGAP19	Rho GTPase activating protein 19
-1.72	DHODH	dihydroorotate dehydrogenase (quinone)
-1.72	C1QBP	complement component 1, q subcomponent binding protein

-1.72	PRIM2	primase, DNA, polypeptide 2 (58kDa)
-1.71	GMNN	geminin, DNA replication inhibitor
-1.71	LYRM7	LYR motif containing 7
-1.71	FAM111A	family with sequence similarity 111, member A
-1.71	DNMT1	DNA (cytosine-5-)-methyltransferase 1
-1.71	TRPV2	transient receptor potential cation channel, subfamily V, member 2
-1.71	PELI1	pellino E3 ubiquitin protein ligase 1
-1.7	SYNE3	spectrin repeat containing, nuclear envelope family member 3
-1.7	PLS1	plastin 1
-1.7	PARP2	poly (ADP-ribose) polymerase 2
-1.7	RAD18	RAD18 E3 ubiquitin protein ligase
-1.7	RFC2	replication factor C (activator 1) 2, 40kDa
-1.7	C24H20orf196	chromosome 24 open reading frame, human C20orf196
-1.7	TUBA4A	tubulin, alpha 4a
-1.69	FUS	FUS RNA binding protein
-1.69	TRERF1	transcriptional regulating factor 1
-1.69	INCENP	inner centromere protein antigens 135/155kDa
-1.68	SPDL1	spindle apparatus coiled-coil protein 1
-1.68	CDC14A	cell division cycle 14A
-1.68	LOC608051	tubulin alpha-3 chain
-1.68	MTHFD1	methylenetetrahydrofolate dehydrogenase (NADP+ dependent) 1, methenyltetrahydrofolate cyclohydrolase, formyltetrahydrofolate synthetase
-1.68	EIF4E3	eukaryotic translation initiation factor 4E family member 3
-1.68	STIL	SCL/TAL1 interrupting locus
-1.68	CKAP5	cytoskeleton associated protein 5
-1.68	TYMS	thymidylate synthetase
-1.68	CDC25B	cell division cycle 25B
-1.68	TMEM107	transmembrane protein 107
-1.67	CD320	CD320 molecule
-1.67	CAMK2A	calcium/calmodulin-dependent protein kinase II alpha
-1.67	CENPP	centromere protein P
-1.67	FILIP1	filamin A interacting protein 1
-1.67	GTPBP8	GTP-binding protein 8 (putative)
-1.66	PCDH7	protocadherin 7
-1.66	INTS1	integrator complex subunit 1
-1.66	PFAS	phosphoribosylformylglycinamidine synthase
-1.66	FAM122B	family with sequence similarity 122B
-1.65	TOMM40	translocase of outer mitochondrial membrane 40 homolog (yeast)
-1.65	ENSCAFG00000000142	ENSCAFG00000000142

-1.65	GLI3	GLI family zinc finger 3
-1.65	TTC9	tetratricopeptide repeat domain 9
-1.64	ENSCAFG00000027573	ENSCAFG00000027573
-1.64	IPO4	importin 4
-1.64	MDH2	malate dehydrogenase 2, NAD (mitochondrial)
-1.64	FBXO27	F-box protein 27
-1.64	ADSL	adenylosuccinate lyase
-1.64	CDC23	cell division cycle 23
-1.64	BRIP1	BRCA1 interacting protein C-terminal helicase 1
-1.63	ICT1	immature colon carcinoma transcript 1
-1.63	AJUBA	ajuba LIM protein
-1.63	CCDC167	coiled-coil domain containing 167
-1.62	AGFG2	ArfGAP with FG repeats 2
-1.62	CD9	CD9 molecule
-1.62	SLCO3A1	solute carrier organic anion transporter family, member 3A1
-1.62	HYOU1	hypoxia up-regulated 1
-1.62	CLDN6	claudin 6
-1.61	CMSS1	cms1 ribosomal small subunit homolog (yeast)
-1.61	MAD1L1	MAD1 mitotic arrest deficient-like 1 (yeast)
-1.61	HNRNPM	heterogeneous nuclear ribonucleoprotein M
-1.61	RHNO1	RAD9-HUS1-RAD1 interacting nuclear orphan 1
-1.6	SLIRP	SRA stem-loop interacting RNA binding protein
-1.6	ATP5G3	ATP synthase, H+ transporting, mitochondrial Fo complex, subunit C3 (subunit 9)
-1.6	POLD2	polymerase (DNA directed), delta 2, accessory subunit
-1.6	LIG1	ligase I, DNA, ATP-dependent
-1.6	LOC488264	histone H1.2
-1.6	SLF1	SMC5-SMC6 complex localization factor 1
-1.59	RHEBL1	Ras homolog enriched in brain like 1
-1.59	NMRAL1	NmrA-like family domain containing 1
-1.59	TTC39B	tetratricopeptide repeat domain 39B
-1.59	MCM6	minichromosome maintenance complex component 6
-1.59	CNOT1	CCR4-NOT transcription complex subunit 1
-1.59	PDE5A	phosphodiesterase 5A, cGMP-specific
-1.59	VRK1	vaccinia related kinase 1
-1.59	OVCA2	OVCA2, serine hydrolase domain containing
-1.58	NEURL1B	neuralized E3 ubiquitin protein ligase 1B
-1.58	CYC1	cytochrome c-1
-1.58	PANX2	pannexin 2
-1.58	NHP2	NHP2 ribonucleoprotein
-1.58	MAP1A	microtubule-associated protein 1A

-1.58	ANP32A	acidic (leucine-rich) nuclear phosphoprotein 32 family, member A
-1.58	ATAD5	ATPase family, AAA domain containing 5
-1.57	MOB3B	MOB kinase activator 3B
-1.57	HLX	H2.0-like homeobox
-1.57	DOLPP1	dolichyldiphosphatase 1
-1.57	SMC1A	structural maintenance of chromosomes 1A
-1.57	SH3BP1	SH3-domain binding protein 1
-1.57	CHRNA5	cholinergic receptor, nicotinic, alpha 5 (neuronal)
-1.57	KCND2	potassium channel, voltage gated Shal related subfamily D, member 2
-1.57	C15H1orf109	chromosome 15 open reading frame, human C1orf109
-1.57	ZGRF1	zinc finger, GRF-type containing 1
-1.57	COQ6	coenzyme Q6 monooxygenase
-1.57	TUBD1	tubulin, delta 1
-1.56	GINS1	GINS complex subunit 1 (Psf1 homolog)
-1.55	POLD3	polymerase (DNA-directed), delta 3, accessory subunit
-1.55	ARL13B	ADP-ribosylation factor-like 13B
-1.55	CRELD2	cysteine-rich with EGF-like domains 2
-1.55	CEP290	centrosomal protein 290kDa
-1.55	KALRN	kalirin, RhoGEF kinase
-1.55	GLIS1	GLIS family zinc finger 1
-1.55	DBF4B	DBF4 zinc finger B
-1.54	PAG1	phosphoprotein membrane anchor with glycosphingolipid microdomains 1
-1.54	SSNA1	Sjogren syndrome nuclear autoantigen 1
-1.54	CEP164	centrosomal protein 164
-1.54	NCLN	nicalin
-1.54	RPP30	ribonuclease P/MRP 30kDa subunit
-1.54	POLQ	polymerase (DNA directed), theta
-1.54	HAT1	histone acetyltransferase 1
-1.54	SHROOM2	shroom family member 2
-1.54	VSIG4	V-set and immunoglobulin domain containing 4
-1.53	LSM7	LSM7 homolog, U6 small nuclear RNA and mRNA degradation associated
-1.53	PDGFRB	platelet-derived growth factor receptor, beta polypeptide
-1.53	MED24	mediator complex subunit 24
-1.53	KPNA2	karyopherin alpha 2 (RAG cohort 1, importin alpha 1)
-1.53	IFT74	intraflagellar transport 74
-1.53	RCAN2	regulator of calcineurin 2
-1.53	PSMA2	proteasome subunit alpha 2
-1.53	CKAP2	cytoskeleton associated protein 2

-1.53	DCPS	decapping enzyme, scavenger
-1.53	NSL1	NSL1, MIS12 kinetochore complex component
-1.52	C20H19orf60	chromosome 20 open reading frame, human C19orf60
-1.52	MTX1	metaxin 1
-1.52	SNRPF	small nuclear ribonucleoprotein polypeptide F
-1.52	SIVA1	SIVA1, apoptosis-inducing factor
-1.52	LOC102155220	39S ribosomal protein L33, mitochondrial pseudogene
-1.52	FEN1	flap structure-specific endonuclease 1
-1.52	HMGN2	high mobility group nucleosomal binding domain 2; dehydrodolichyl diphosphate synthase subunit
-1.52	GPR162	G protein-coupled receptor 162
-1.52	HMBS	hydroxymethylbilane synthase
-1.52	FUS	FUS RNA binding protein
-1.52	HSD3B7	hydroxy-delta-5-steroid dehydrogenase, 3 beta- and steroid delta-isomerase 7
-1.52	LIN9	lin-9 DREAM MuvB core complex component
-1.52	SLC38A5	solute carrier family 38, member 5
-1.51	FAM49A	family with sequence similarity 49, member A
-1.51	FASN	fatty acid synthase
-1.51	SYT7	synaptotagmin VII
-1.51	OAS2	2-5-oligoadenylate synthetase 2, 69/71kDa
-1.51	EFHD2	EF-hand domain family, member D2
-1.51	RTFDC1	replication termination factor 2 domain containing 1
-1.51	LOC610565	uncharacterized LOC610565
-1.51	NRG2	neuregulin 2
-1.51	MINOS1	mitochondrial inner membrane organizing system 1
-1.51	SPIDR	scaffolding protein involved in DNA repair
-1.51	NT5C	5, 3-nucleotidase, cytosolic
-1.51	TLCD1	TLC domain containing 1
1.51	KLC4	kinesin light chain 4
1.51	TBC1D2	TBC1 domain family, member 2
1.51	ANKRD50	ankyrin repeat domain 50
1.51	RAB32	RAB32, member RAS oncogene family
1.51	PHF20	PHD finger protein 20
1.51	SDC4	syndecan 4
1.51	SMIM3	small integral membrane protein 3
1.51	CLCN7	chloride channel, voltage-sensitive 7
1.52	TTC21A	tetratricopeptide repeat domain 21A
1.52	RPS3	ribosomal protein S3
1.52	ZC3HAV1L	zinc finger CCCH-type, antiviral 1-like
1.52	MARCH9	membrane-associated ring finger (C3HC4) 9
1.52	ADAMTS10	ADAM metallopeptidase with thrombospondin type 1 motif, 10

1.52	P4HA3	prolyl 4-hydroxylase, alpha polypeptide III
1.52	MIR27B	microRNA mir-27b
1.52	VAMP5	vesicle-associated membrane protein 5
1.52	STK17A	serine/threonine kinase 17a
1.52	ADGRA3	adhesion G protein-coupled receptor A3
1.52	GALNS	galactosamine (N-acetyl)-6-sulfatase
1.53	RPL21	60S ribosomal protein L21
1.53	CDH24	cadherin 24, type 2
1.53	PDPK1	3-phosphoinositide dependent protein kinase 1; potassium channel tetramerization domain containing 5
1.53	RAB33B	RAB33B, member RAS oncogene family
1.53	HEXB	hexosaminidase B (beta polypeptide)
1.53	DLG2	discs, large homolog 2 (Drosophila)
1.53	TMCO3	transmembrane and coiled-coil domains 3
1.53	NPC2	Niemann-Pick disease, type C2
1.53	ABCA8	ATP-binding cassette, sub-family A (ABC1), member 8
1.54	RHOBTB3	Rho-related BTB domain containing 3
1.54	EVL	Enah/Vasp-like
1.54	EED	embryonic ectoderm development
1.54	SEC61G	Sec61 translocon gamma subunit
1.54	CREB3L2	cAMP responsive element binding protein 3-like 2
1.54	CTSK	cathepsin K
1.54	CFLAR	CASP8 and FADD-like apoptosis regulator
1.54	TAF1A	TATA box binding protein (TBP)-associated factor, RNA polymerase I, A, 48kDa
1.55	ITM2C	integral membrane protein 2C
1.55	SGCD	sarcoglycan, delta (35kDa dystrophin-associated glycoprotein)
1.55	TMEM132A	transmembrane protein 132A
1.55	HSD11B1L	hydroxysteroid (11-beta) dehydrogenase 1-like
1.55	GHRH	growth hormone releasing hormone
1.55	MCC	mutated in colorectal cancers
1.55	SUCO	SUN domain containing ossification factor
1.55	RILP	Rab interacting lysosomal protein
1.56	ABHD17B	abhydrolase domain containing 17B
1.56	ASIC2	acid sensing (proton gated) ion channel 2
1.56	SNORD50	SNORD50
1.56	SNRNP48	small nuclear ribonucleoprotein U11/U12 subunit 48
1.56	SLC37A2	solute carrier family 37 (glucose-6-phosphate transporter), member 2
1.56	DNM3	dynamin 3
1.56	TTC25	tetratricopeptide repeat domain 25
1.57	TTLL4	tubulin tyrosine ligase-like family member 4

1.57	HIF1A	hypoxia inducible factor 1, alpha subunit (basic helix-loop-helix transcription factor)
1.57	KDM5B	lysine (K)-specific demethylase 5B
1.57	TNFAIP2	tumor necrosis factor, alpha-induced protein 2
1.57	KLHL23	kelch-like family member 23
1.57	SYNGR3	synaptogyrin 3
1.58	MTURN	maturin, neural progenitor differentiation regulator homolog (Xenopus)
1.58	SLC16A1	solute carrier family 16 (monocarboxylate transporter), member 1
1.58	FAM219B	family with sequence similarity 219, member B
1.58	KLHL24	kelch-like family member 24
1.58	TCEANC2	transcription elongation factor A (SII) N-terminal and central domain containing 2
1.59	GAB2	GRB2-associated binding protein 2
1.59	SHOX2	short stature homeobox 2
1.59	METTL12	methyltransferase like 12
1.59	SNX29	sorting nexin 29
1.59	SLC25A36	solute carrier family 25 (pyrimidine nucleotide carrier), member 36
1.59	FXYD6	FXYD domain containing ion transport regulator 6
1.59	MTCP1	mature T-cell proliferation 1
1.6	CBLB	Cbl proto-oncogene B, E3 ubiquitin protein ligase
1.6	ZSCAN2	zinc finger and SCAN domain containing 2
1.6	PLEKHA1	pleckstrin homology domain containing, family A (phosphoinositide binding specific) member 1
1.6	PEX10	peroxisomal biogenesis factor 10
1.6	TMEM116	transmembrane protein 116
1.6	C1GALT1	core 1 synthase, glycoprotein-N-acetylgalactosamine 3-beta-galactosyltransferase 1
1.6	FLRT2	fibronectin leucine rich transmembrane protein 2
1.6	LOC106559978	low-density lipoprotein receptor-related protein 5-like; low density lipoprotein receptor-related protein 5
1.6	RHOH	ras homolog family member H
1.6	MIR197	microRNA mir-197
1.61	TTC21A	tetratricopeptide repeat domain 21A
1.61	DAPK3	death-associated protein kinase 3
1.61	MEX3A	mex-3 RNA binding family member A
1.61	ZHX2	zinc fingers and homeoboxes 2
1.61	SBNO2	strawberry notch homolog 2 (Drosophila)
1.61	MTIF3	mitochondrial translational initiation factor 3
1.62	KDM4C	lysine (K)-specific demethylase 4C
1.62	RBPJL	recombination signal binding protein for immunoglobulin kappa J region-like

1.62	TIAM2	T-cell lymphoma invasion and metastasis 2
1.62	SYT5	synaptotagmin V
1.62	CHRNA7	cholinergic receptor, nicotinic, alpha 7 (neuronal)
1.62	GALNT16	polypeptide N-acetylgalactosaminyltransferase 16
1.62	GALC	galactosylceramidase
1.63	RPS8	ribosomal protein S8
1.63	MIR421	microRNA 421
1.63	TMEM51	transmembrane protein 51
1.63	ZNF280B	zinc finger protein 280B
1.63	PLAGL1	pleiomorphic adenoma gene-like 1
1.63	LOC474642	transmembrane emp24 domain-containing protein 9-like
1.64	SUGCT	succinyl-CoA:glutarate-CoA transferase
1.64	ZNF521	zinc finger protein 521
1.64	LOC102154557	uncharacterized LOC102154557
1.64	CCDC113	coiled-coil domain containing 113
1.64	KLF5	Kruppel-like factor 5 (intestinal)
1.64	RORA	RAR-related orphan receptor A
1.64	MOSPD2	motile sperm domain containing 2
1.65	DAAM1	dishevelled associated activator of morphogenesis 1
1.65	MRC2	mannose receptor, C type 2
1.65	HSD17B8	hydroxysteroid (17-beta) dehydrogenase 8
1.65	LRRFIP2	leucine rich repeat (in FLII) interacting protein 2
1.65	CCT6A	chaperonin containing TCP1 subunit 6A
1.65	SNORD60	SNORD60
1.65	MOCOS	molybdenum cofactor sulfurase
1.65	SGSH	N-sulfoglucosamine sulfohydrolase
1.66	KDM3A	lysine (K)-specific demethylase 3A
1.66	LIX1L	limb and CNS expressed 1 like
1.66	MAGI2	membrane associated guanylate kinase, WW and PDZ domain containing 2
1.66	NPC1	Niemann-Pick disease, type C1
1.66	CLCN4	chloride channel, voltage-sensitive 4
1.66	VEZT	vezatin, adherens junctions transmembrane protein
1.67	NEIL1	nei-like DNA glycosylase 1
1.67	RWDD2A	RWD domain containing 2A
1.67	HHIPL1	HHIP-like 1
1.67	C16H8orf4	chromosome 16 open reading frame, human C8orf4
1.67	NMRK1	nicotinamide riboside kinase 1
1.68	FNTB	farnesyltransferase, CAAX box, beta
1.68	FOXRED2	FAD-dependent oxidoreductase domain containing 2
1.68	LOC100687306	olfactory receptor 12-like

1.68	SEZ6L	seizure related 6 homolog (mouse)-like
1.68	MYOZ3	myozenin 3
1.69	GRAP2	GRB2-related adaptor protein 2
1.69	RPS6KA5	ribosomal protein S6 kinase, 90kDa, polypeptide 5
1.69	SLC35G2	solute carrier family 35, member G2
1.69	PARK2	parkin RBR E3 ubiquitin protein ligase
1.69	NMS	neuromedin S
1.69	MAT2A	methionine adenosyltransferase II, alpha
1.69	SNX32	sorting nexin 32
1.7	SOX4	SRY (sex determining region Y)-box 4
1.7	MFHAS1	malignant fibrous histiocytoma amplified sequence 1
1.71	CTSF	cathepsin F
1.71	CPE	carboxypeptidase E
1.71	TLE1	transducin-like enhancer of split 1 (E(sp1) homolog, Drosophila)
1.72	SNORD89	SNORD89
1.72	BTF3L4	basic transcription factor 3-like 4
1.72	FBLN2	fibulin 2
1.73	SNORD77	SNORD77
1.73	SLC44A1	solute carrier family 44 (choline transporter), member 1
1.73	TIMP3	TIMP metallopeptidase inhibitor 3
1.73	SNORD99	SNORD99
1.74	CDH3	cadherin 3, type 1, P-cadherin (placental)
1.74	NFKBIZ	nuclear factor of kappa light polypeptide gene enhancer in B-cells inhibitor, zeta
1.74	SLC35D3	solute carrier family 35, member D3
1.75	LGMN	legumain
1.75	ARV1	ARV1 homolog, fatty acid homeostatsis modulator
1.75	PLBD2	phospholipase B domain containing 2
1.75	C28H10orf88	chromosome 28 open reading frame, human C10orf88
1.75	GPR137B	G protein-coupled receptor 137B
1.76	IGF2BP2	insulin-like growth factor 2 mRNA binding protein 2
1.77	SUGCT	succinyl-CoA:glutarate-CoA transferase
1.77	PPP1R26	protein phosphatase 1, regulatory subunit 26
1.78	VOPP1	vesicular, overexpressed in cancer, prosurvival protein 1
1.78	OLFM2	olfactomedin 2
1.79	RPL21	60S ribosomal protein L21
1.79	P2RX2	purinergic receptor P2X, ligand gated ion channel, 2
1.79	BICD1	bicaudal D homolog 1 (Drosophila)
1.79	VMO1	vitelline membrane outer layer 1 homolog (chicken)
1.8	LOC102151205	uncharacterized LOC102151205

1.8	APOE	apolipoprotein E
1.81	KLF11	Kruppel-like factor 11
1.81	SHROOM4	shroom family member 4
1.81	RASL11B	RAS-like, family 11, member B
1.82	GABBR1	gamma-aminobutyric acid (GABA) B receptor, 1
1.82	FKBP7	FK506 binding protein 7
1.82	STARD6	StAR-related lipid transfer (START) domain containing 6
1.82	FAM131B	family with sequence similarity 131, member B
1.82	P2RY11	suppressor of SWI4 1 homolog
1.83	LOC490277	potassium voltage-gated channel subfamily H member 1
1.83	CXCL16	chemokine (C-X-C motif) ligand 16
1.83	TXNIP	thioredoxin interacting protein
1.84	CCBL1	cysteine conjugate-beta lyase, cytoplasmic
1.84	PPP1R15A	protein phosphatase 1, regulatory subunit 15A
1.84	LOC487173	protocadherin beta-6
1.84	SYNPO	synaptopodin
1.84	RASSF1	Ras association (RalGDS/AF-6) domain family member 1
1.85	PLD2	phospholipase D2
1.85	SUGCT	succinyl-CoA:glutarate-CoA transferase
1.85	C15H1orf228	chromosome 15 open reading frame, human C1orf228
1.85	CTNNAL1	catenin (cadherin-associated protein), alpha-like 1
1.85	ZNF395	zinc finger protein 395
1.86	DBNDD1	dysbindin (dystrobrevin binding protein 1) domain containing 1
1.86	TNFSF10	tumor necrosis factor (ligand) superfamily, member 10
1.86	LOC100686073	metallothionein-1
1.86	GMDS	GDP-mannose 4,6-dehydratase
1.87	IGLON5	IgLON family member 5
1.87	BDH2	3-hydroxybutyrate dehydrogenase, type 2
1.88	CD200	CD200 molecule
1.88	GPNMB	glycoprotein (transmembrane) nmb
1.88	FBXL2	F-box and leucine-rich repeat protein 2
1.89	C1QTNF5	C1q and TNF related 5
1.89	DOK5	docking protein 5
1.9	PDCD4	programmed cell death 4 (neoplastic transformation inhibitor)
1.9	KIAA1217	KIAA1217 ortholog
1.91	KCTD11	potassium channel tetramerization domain containing 11
1.91	PRRG1	proline rich Gla (G-carboxyglutamic acid) 1
1.91	EPOR	erythropoietin receptor

1.92	BTG1	B-cell translocation gene 1, anti-proliferative
1.92	STRIP2	striatin interacting protein 2
1.93	VEGFA	vascular endothelial growth factor A
1.93	FNIP2	folliculin interacting protein 2
1.93	SCARB2	scavenger receptor class B, member 2
1.93	PIPOX	pipecolic acid oxidase
1.94	SLC26A5	solute carrier family 26 (anion exchanger), member 5
1.94	ELOVL7	ELOVL fatty acid elongase 7
1.94	MIR8810	microRNA mir-8810; adenosine monophosphate deaminase 3
1.95	PTPN5	protein tyrosine phosphatase, non-receptor type 5 (striatum-enriched)
1.95	MAPK13	mitogen-activated protein kinase 13
1.96	UTP23	UTP23, small subunit (SSU) processome component, homolog (yeast)
1.96	HPS3	Hermansky-Pudlak syndrome 3
1.96	RALGDS	ral guanine nucleotide dissociation stimulator
1.97	CNTN3	contactin 3 (plasmacytoma associated)
1.97	FAM71E1	family with sequence similarity 71, member E1
1.99	SLC2A1	solute carrier family 2 (facilitated glucose transporter), member 1
2	TSPAN11	tetraspanin 11
2.01	MIR23A	microRNA mir-23a
2.02	GALNT10	polypeptide N-acetylgalactosaminyltransferase 10
2.03	CAECAM1	carcinoembryonic antigen-related cell adhesion molecule 25
2.04	MGAT3	mannosyl (beta-1,4-)-glycoprotein beta-1,4-N-acetylglucosaminyltransferase
2.05	MIR27A	microRNA mir-27a
2.05	PLAG1	pleiomorphic adenoma gene 1
2.05	PARK2	parkin RBR E3 ubiquitin protein ligase
2.05	ATF7IP2	activating transcription factor 7 interacting protein 2
2.06	MT2A	metallothionein 1H
2.07	PABPC4	poly(A) binding protein cytoplasmic 4
2.07	ZFHX2	zinc finger homeobox 2
2.08	TMEM71	transmembrane protein 71
2.08	UCP2	uncoupling protein 2 (mitochondrial, proton carrier)
2.1	PRSS12	protease, serine, 12 (neurotrypsin, motopsin)
2.1	CHL1	cell adhesion molecule L1-like
2.11	ARHGAP24	Rho GTPase activating protein 24
2.11	MTHFS	5,10-methenyltetrahydrofolate synthetase (5-formyltetrahydrofolate cyclo-ligase)
2.11	FLCN	folliculin
2.13	TULP4	tubby like protein 4

2.14	ASAH1	N-acylsphingosine amidohydrolase (acid ceramidase) 1
2.14	CHSY3	chondroitin sulfate synthase 3
2.15	CAPN3	calpain 3
2.15	OSGIN2	oxidative stress induced growth inhibitor family member 2
2.16	STK17B	serine/threonine kinase 17b
2.16	MSX1	msh homeobox 1
2.17	PLXNB1	plexin B1
2.17	RPL13	ribosomal protein L13
2.18	WNT5A	wingless-type MMTV integration site family, member 5A
2.19	FGD6	FYVE, RhoGEF and PH domain containing 6
2.19	ANKRD37	ankyrin repeat domain 37
2.21	MIR24-1	microRNA mir-24-1
2.22	DTNB	dystrobrevin, beta
2.23	DPYD	dihydropyrimidine dehydrogenase
2.25	ELOVL4	ELOVL fatty acid elongase 4
2.25	TAL1	T-cell acute lymphocytic leukemia 1
2.25	MIR21	microRNA mir-21
2.27	LRRC6	leucine rich repeat containing 6
2.29	MYO1E	myosin IE
2.29	ERP27	endoplasmic reticulum protein 27; Rho GDP dissociation inhibitor (GDI) beta
2.3	PTPRN2	protein tyrosine phosphatase, receptor type, N polypeptide 2
2.3	PLAU	plasminogen activator, urokinase
2.31	RANBP3L	RAN binding protein 3-like
2.32	TSGA10	testis specific, 10
2.33	FGG	fibrinogen gamma chain
2.33	PCBD1	pterin-4 alpha-carbinolamine dehydratase/dimerization cofactor of hepatocyte nuclear factor 1 alpha
2.33	MIR221	microRNA mir-221
2.36	MAFB	v-maf avian musculoaponeurotic fibrosarcoma oncogene homolog B
2.37	FAM180A	family with sequence similarity 180, member A
2.39	IL1R1	interleukin 1 receptor, type I
2.4	MIR22	microRNA mir-22
2.42	OTUD1	OTU deubiquitinase 1
2.47	PTGDS	prostaglandin D2 synthase 21kDa (brain)
2.53	LOC491239	UDP-N-acetylhexosamine pyrophosphorylase-like protein 1
2.55	MIR29A	microRNA mir-29a
2.56	STEAP1	six transmembrane epithelial antigen of the prostate 1

2.56	Y_RNA	Y RNA
2.58	QPCT	glutaminyl-peptide cyclotransferase
2.58	CXADR	coxsackie virus and adenovirus receptor
2.59	BMP6	bone morphogenetic protein 6
2.61	CPXM1	carboxypeptidase X (M14 family), member 1
2.65	WFDC5	WAP four-disulfide core domain 5
2.66	FHIT	fragile histidine triad
2.67	BAMBI	BMP and activin membrane-bound inhibitor
2.69	HK2	hexokinase 2
2.71	MFAP2	microfibrillar-associated protein 2
2.71	DOCK5	dedicator of cytokinesis 5
2.73	GCNT4	glucosaminyl (N-acetyl) transferase 4, core 2
2.73	FAM216B	family with sequence similarity 216, member B
2.73	MIR199-2	microRNA mir-199-2
2.78	CDS1	CDP-diacylglycerol synthase (phosphatidate cytidylyltransferase) 1
2.85	HPSE	heparanase
2.9	LOC487977	cell surface glycoprotein CD200 receptor 1
2.91	DDAH1	dimethylarginine dimethylaminohydrolase 1
2.93	TMEM86A	transmembrane protein 86A
3.04	SERTAD4	SERTA domain containing 4
3.05	DCLK2	doublecortin-like kinase 2
3.06	EMB	embigin
3.09	MFAP3L	microfibrillar-associated protein 3-like
3.09	CCL7	chemokine (C-C motif) ligand 7
3.12	PTHLH	parathyroid hormone-like hormone
3.14	SLC2A3	solute carrier family 2 (facilitated glucose transporter), member 3
3.19	SLC2A1	solute carrier family 2 (facilitated glucose transporter), member 1
3.27	MATN4	matrilin 4
3.29	TUBB4A	tubulin, beta 4A class IVa
3.3	RAPGEF4	Rap guanine nucleotide exchange factor (GEF) 4
3.38	KIF26B	kinesin family member 26B
3.57	HEPACAM	hepatic and glial cell adhesion molecule
3.58	CADM4	cell adhesion molecule 4
3.62	AQP11	aquaporin 11
3.68	SPINT1	serine peptidase inhibitor, Kunitz type 1
3.68	SOD3	superoxide dismutase 3, extracellular
3.76	IGF2BP3	insulin-like growth factor 2 mRNA binding protein 3
3.8	PTGS1	prostaglandin-endoperoxide synthase 1 (prostaglandin G/H synthase and cyclooxygenase)
3.82	TMEM100	transmembrane protein 100
3.94	LOC487628	thrombospondin type-1 domain-containing protein 4

4.02	CA12	carbonic anhydrase XII
4.05	BPHL	biphenyl hydrolase-like (serine hydrolase)
4.22	TGM2	transglutaminase 2
4.24	CLDN1	claudin 1
4.47	DHRS9	dehydrogenase/reductase (SDR family) member 9
4.65	SERPINI1	serpin peptidase inhibitor, clade I (neuroserpin), member 1
4.65	EREG	epiregulin
4.96	KERA	keratocan
5.05	PKHD1L1	polycystic kidney and hepatic disease 1 (autosomal recessive)-like 1
5.48	CHRM2	cholinergic receptor, muscarinic 2
5.86	CASP14	caspase 14, apoptosis-related cysteine peptidase
5.97	PKHD1L1	polycystic kidney and hepatic disease 1 (autosomal recessive)-like 1
6.15	PKHD1L1	polycystic kidney and hepatic disease 1 (autosomal recessive)-like 1
6.29	ADAMTSL2	ADAMTS-like 2
6.44	SLC4A11	solute carrier family 4, sodium borate transporter, member 11
6.81	CXCL8	chemokine (C-X-C motif) ligand 8
6.86	LOC608987	cyclin-J-like protein; cyclin J-like
7.01	PKHD1L1	polycystic kidney and hepatic disease 1 (autosomal recessive)-like 1
7.51	BPI	bactericidal/permeability-increasing protein
9.82	HSD17B14	hydroxysteroid (17-beta) dehydrogenase 14
9.83	EDNRB	endothelin receptor type B
13.69	LOC479476	arachidonate 12-lipoxygenase, 12S-type
16.05	HECW1	HECT, C2 and WW domain containing E3 ubiquitin protein ligase 1
68.9	ACKR4	atypical chemokine receptor 4
