# Supplementary material for: Evaluation of canine 2D cell cultures as models of myxomatous mitral valve degeneration
Source: PLoS One. 2019 Aug 15;14(8):e0221126. doi: 10.1371/journal.pone.0221126 (PMC6695117; doi:10.1371/journal.pone.0221126)
Supplement: S7 Table — All gene showed the same direction of change (down) except for MALD1. (PDF) [file pone.0221126.s007.pdf]

S7 Table. 102 shared differentially expressed genes in the TGFβ1-treated qVICs and aVICs datasets compared to un-treated qVICs. All gene showed the same direction of change (down) except for *MALD1*.

Fold Change in TGF treated qVICS	Fold Change aVICs	Gene symbol	Gene name
-5.47	-5.1	ENSCAFG00000031869	
-5.11	-7.89	PDK4	pyruvate dehydrogenase kinase, isozyme 4
-4.41	-1.88	NDP	Norrie disease (pseudoglioma)
-4.19	-5.18	NPPC	natriuretic peptide C
-4.1	-5.53	FRMD5	FERM domain containing 5
-3.41	-3.11	CCDC3	coiled-coil domain containing 3
-3.39	-12.26	GJA5	gap junction protein, alpha 5, 40kDa
-3.09	-3.98	BMPER	BMP binding endothelial regulator
-3.01	-3.58	RRAD	Ras-related associated with diabetes
-2.87	-4.45	ZDHHC14	zinc finger, DHHC-type containing 14
-2.8	-5.72	ATF7IP2	activating transcription factor 7 interacting protein 2
-2.77	-10.19	SPINT1	serine peptidase inhibitor, Kunitz type 1
-2.74	-5.26	STRIP2	striatin interacting protein 2
-2.69	-2.9	ARL4D	ADP-ribosylation factor-like 4D
-2.6	-2.94	OLFML2A	olfactomedin-like 2A
-2.48	-2.58	SLC25A42	solute carrier family 25, member 42
-2.17	-4.45	ZDHHC14	zinc finger, DHHC-type containing 14
-2.14	-3.18	KCND3	potassium channel, voltage gated Shal related subfamily D, member 3
-2.14	-2.6	P2RY1	purinergic receptor P2Y, G-protein coupled, 1
-2.06	-4.29	SELL	selectin L
-1.96	-4.23	IGF1	insulin-like growth factor 1 (somatomedin C)
-1.96	-2.14	SLC10A6	solute carrier family 10 (sodium/bile acid cotransporter), member 6
-1.95	-1.65	NET1	neuroepithelial cell transforming 1

-1.93	-1.82	LGALSL	lectin, galactoside-binding-like
-1.87	-1.95	FAM19A4	family with sequence similarity 19 (chemokine (C-C motif)-like), member A4
-1.86	-2.26	ADGRF2	adhesion G protein-coupled receptor F2
-1.83	-2.9	HS3ST1	heparan sulfate-glucosamine 3-sulfotransferase 1
-1.83	-2.79	VCAM1	vascular cell adhesion molecule 1
-1.81	-2.29	YPEL1	yippee-like 1
-1.81	-3.06	GRAP2	GRB2-related adaptor protein 2
-1.81	-4.02	PRSS12	protease, serine 12
-1.81	-4.02	PRSS12	protease, serine 12
-1.78	-4.02	PRSS12	protease, serine 12
-1.78	-3.47	SORBS1	sorbin and SH3 domain containing 1
-1.76	-1.85	NRP1	neuropilin 1
-1.75	-1.53	LIFR	leukemia inhibitory factor receptor alpha
-1.74	-2.01	LYPD6	LY6/PLAUR domain containing 6
-1.73	-1.88	ZFP30	ZFP30 zinc finger protein
-1.73	-1.96	NFKBIA	nuclear factor of kappa light polypeptide gene enhancer in B-cells inhibitor, alpha
-1.72	-1.67	HBEGF	heparin-binding EGF-like growth factor
-1.71	-2.56	HSPA2	heat shock 70kDa protein 2
-1.68	-2.04	CACNG4	calcium channel, voltage-dependent, gamma subunit 4
-1.67	-4.32	CXADR	coxsackie virus and adenovirus receptor
-1.67	-2.36	SLC22A23	solute carrier family 22, member 23
-1.65	-1.61	PLCB1	phospholipase C, beta 1 (phosphoinositide-specific)
-1.63	-3.88	MAFB	v-maf avian musculoaponeurotic fibrosarcoma oncogene homolog B
-1.62	-1.65	MAPT	microtubule-associated protein tau
-1.6	-1.82	LOC474850	heat shock 70 kDa protein 1-like

-1.6	-1.9	ADAMTS1	ADAM metallopeptidase with thrombospondin type 1 motif, 1
-1.59	-1.9	RASL10B	RAS-like, family 10, member B
-1.58	-1.6	EPAS1	endothelial PAS domain protein 1
-1.56	-2.25	TSPAN13	tetraspanin 13
-1.55	-1.54	TSC22D1	TSC22 domain family, member 1
-1.53	-1.96	PPAP2B	phosphatidic acid phosphatase type 2B
-1.52	2.12	KIF18B	kinesin family member 18B
-1.52	-1.9	LOC481227	neuronal-specific septin-3
-1.52	-1.76	ENSCAFG00000022342	Chromosome 1: 56,225,495-56,225,821
-1.52	1.56	MAMLD1	mastermind like domain containing 1
1.53	1.56	LPCAT3	lysophosphatidylcholine acyltransferase 3
1.54	2.39	ENSCAFG00000027228	Chromosome 3: 28,138,305-28,138,408
1.55	1.88	SREBF1	sterol regulatory element binding transcription factor 1
1.56	1.71	VDR	vitamin D (1,25-dihydroxyvitamin D3) receptor
1.57	1.98	SMTN	smoothelin
1.57	1.61	EPHB3	EPH receptor B3
1.58	2.9	TPM2	tropomyosin 2
1.61	1.8	YIF1B	Yip1 interacting factor homolog B (S. cerevisiae)
1.62	2.28	ACAT2	acetyl-CoA acetyltransferase 2
1.63	1.6	MIR107	microRNA mir-107
1.64	1.52	ARMC9	armadillo repeat containing 9
1.66	2.74	BTK	Bruton agammaglobulinemia tyrosine kinase
1.67	3.07	TPM2	tropomyosin 2 (beta)
1.67	1.67	C1QTNF3	C1q and tumor necrosis factor related protein 3; alpha-methylacyl-CoA racemase
1.73	1.77	TMPO	thymopoietin
1.74	1.67	CYP51A1	cytochrome P450, family 51, subfamily A, polypeptide 1
1.76	1.75	TSPAN6	tetraspanin 6
1.77	3.1	ST5	suppression of tumorigenicity 5
1.79	1.82	UACA	uveal autoantigen with coiled-coil domains and ankyrin repeats

1.83	2.9	TPM2	tropomyosin 2
1.88	1.57	FAM13A	family with sequence similarity 13, member A
1.88	3.1	ST5	suppression of tumorigenicity 5
1.95	2.7	WFIKKN2	WAP, follistatin/kazal, immunoglobulin, kunitz and netrin domain containing 2
1.96	7.69	ACTA2	actin, alpha 2, smooth muscle, aorta
2.07	2.99	TM7SF2	transmembrane 7 superfamily member 2
2.1	9.06	NEDD9	neural precursor cell expressed, developmentally down-regulated 9
2.1	2.47	COL15A1	collagen, type XV, alpha 1
2.13	3.78	TAGLN	transgelin
2.15	1.67	MFSD2A	major facilitator superfamily domain containing 2A
2.23	1.66	ADCY7	adenylate cyclase 7
2.25	2.01	IGDCC4	immunoglobulin superfamily, DCC subclass, member 4
2.28	2.08	MEOX1	mesenchyme homeobox 1
2.39	1.61	ANKRD1	ankyrin repeat domain 1 (cardiac muscle)
2.43	2.05	CRABP2	cellular retinoic acid binding protein 2
2.47	1.81	PAPPA	pregnancy-associated plasma protein A, pappalysin 1
2.5	2	FZD2	frizzled class receptor 2
2.51	3.1	ST5	suppression of tumorigenicity 5
2.53	3.2	CH25H	cholesterol 25-hydroxylase
2.68	1.69	PMEPA1	prostate transmembrane protein, androgen induced 1
2.85	3.8	LOC486400	gamma-glutamyltranspeptidase 1
3.19	3.32	NOX4	NADPH oxidase 4
3.71	9.21	TFPI2	tissue factor pathway inhibitor 2
4.15	3.66	SRPX2	sushi-repeat containing protein, X-linked 2
4.71	6.73	FAP	fibroblast activation protein, alpha
