# Supplementary material for: Evaluation of canine 2D cell cultures as models of myxomatous mitral valve degeneration
Source: PLoS One. 2019 Aug 15;14(8):e0221126. doi: 10.1371/journal.pone.0221126 (PMC6695117; doi:10.1371/journal.pone.0221126)
Supplement: S8 Table — A. qVICs/aVICs; B. TGFβ1-qVICs/qVICs; C. SB431542-aVICs/aVICs; D. TGFβ1-qVICs/aVICs; E. shared gene set for TGFB1-treated qVICs and aVICs. (PDF) [file pone.0221126.s008.pdf]

S8 Table. Top 10 up and down regulated GO terms for different data set comparisons.
A qVICs/aVICs; B TGFβ1-qVICs/qVICs; C SB431542-aVICs/aVICs; D TGFβ1-qVICs/aVICs; E shared gene set for TGFB1-treated qVICs and aVICs

A.

	Category	Term	Gene count	P-value	FDR
Up-regulated	GOTERM_MF_DIRECT	ATP binding	73	8.12E-13	1.13E-09
	GOTERM_BP_DIRECT	DNA replication initiation	11	8.27E-11	1.33E-07
	GOTERM_BP_DIRECT	Chromosome segregation	14	1.53E-10	2.45E-07
	GOTERM_CC_DIRECT	Kinesin complex	13	3.38E-09	4.46E-06
	GOTERM_BP_DIRECT	Mitotic sister chromatid segregation	9	1.37E-08	2.20E-05
	GOTERM_BP_DIRECT	Microtubule-based movement	14	1.54E-08	2.47E-05
	GOTERM_CC_DIRECT	Midbody	15	3.16E-08	4.17E-05
	GOTERM_CC_DIRECT	Nuclear chromosome, telomeric region	15	9.51E-08	1.26E-04
	GOTERM_CC_DIRECT	Nucleoplasm	67	2.13E-07	2.82E-04
	GOTERM_BP_DIRECT	Cell division	11	5.07E-07	8.14E-04
Down-regulated	GOTERM_CC_DIRECT	Intracellular	36	3.71E-06	0.00466
	GOTERM_CC_DIRECT	Lysosome	12	6.38E-05	0.08019
	GOTERM_BP_DIRECT	Small GTPase mediated signal transduction	15	7.56E-05	0.12256
	GOTERM_BP_DIRECT	PDGF receptor signaling pathway	6	9.44E-05	0.15311
	GOTERM_BP_DIRECT	intracellular signal transduction	18	1.61E-04	0.26021
	GOTERM_MF_DIRECT	Guanyl-nucleotide exchange factor activity	7	9.98E-04	1.33324
	GOTERM_MF_DIRECT	Semaphorin receptor activity	4	0.00208	2.753
	GOTERM_BP_DIRECT	Vasculogenesis	6	0.00263	4.18228
	GOTERM_MF_DIRECT	Rho guanyl-nucleotide exchange factor activity	7	0.00295	3.88544
	GOTERM_CC_DIRECT	Endosome	9	0.00815	9.77246

B.

	Category	Term	Gene count	P-value	FDR
Up-regulated	GOTERM_BP_DIRECT	Positive regulation of release of cytochrome c from mitochondria	4	2.64E-04	0.38132
	GOTERM_CC_DIRECT	Extracellular matrix	5	0.00387	4.2409
	GOTERM_CC_DIRECT	Extracellular space	12	0.01687	17.3279
	GOTERM_BP_DIRECT	Cholesterol metabolic process	3	0.01837	23.5133
	GOTERM_BP_DIRECT	Osteoblast differentiation	4	0.01841	23.5522
	GOTERM_MF_DIRECT	Acylglycerophosphocholine O-acyltransferase activity	2	0.02591	26.6683
	GOTERM_MF_DIRECT	GTPase activator activity	5	0.02659	27.2671
	GOTERM_CC_DIRECT	Extracellular exosome	22	0.02729	26.6109
	GOTERM_CC_DIRECT	Collagen trimer	3	0.03073	29.4616
	GOTERM_CC_DIRECT	Cytoplasm	25	0.03698	34.3791
Down-regulated	GOTERM_BP_DIRECT	Actomyosin structure organization	4	7.14E-04	1.05707
	GOTERM_CC_DIRECT	Cell surface	10	8.44E-04	0.96142
	GOTERM_CC_DIRECT	Integral component of plasma membrane	15	0.00108	1.23267
	GOTERM_BP_DIRECT	Positive regulation of Ras protein signal transduction	3	0.0048	6.90528
	GOTERM_BP_DIRECT	Cardiac conduction system development	2	0.01617	21.529
	GOTERM_MF_DIRECT	E-box binding	3	0.01709	18.5306
	GOTERM_CC_DIRECT	Extracellular space	13	0.01817	18.9324
	GOTERM_BP_DIRECT	Positive regulation of ERK1 and ERK2 cascade	5	0.01976	25.684
	GOTERM_CC_DIRECT	Cell body	3	0.02099	21.5577
	GOTERM_BP_DIRECT	Outflow tract morphogenesis	3	0.02296	29.2174

C.

	Category	Term	Gene count	P-value	FDR
Up-regulated	GOTERM_CC_DIRECT	Lysosome	6	0.001142	1.24282
	GOTERM_BP_DIRECT	Angiogenesis	5	0.00706	9.51341
	GOTERM_BP_DIRECT	Positive regulation of TOR signaling	3	0.01016	13.4181
	GOTERM_BP_DIRECT	Nephron tubule epithelial cell differentiation	2	0.013912	17.9349
	GOTERM_CC_DIRECT	Mitochondrial membrane	3	0.01667	16.8095
	GOTERM_MF_DIRECT	Creatine kinase activity	2	0.019243	20.4883
	GOTERM_CC_DIRECT	Gtr1-Gtr2 GTPase complex	2	0.0197	19.5725
	GOTERM_BP_DIRECT	Renal tubule morphogenesis	2	0.020796	25.6583
	GOTERM_BP_DIRECT	Epithelial cell proliferation involved in renal tubule morphogenesis	2	0.020796	25.6583
	GOTERM_BP_DIRECT	Glomerulus morphogenesis	2	0.020796	25.6583
Down-regulated	GOTERM_BP_DIRECT	Collagen fibril organization	6	1.28E-06	0.00187
	GOTERM_MF_DIRECT	Extracellular matrix structural constituent	5	6.49E-05	0.07331
	GOTERM_BP_DIRECT	Positive regulation of epithelial to mesenchymal transition	4	6.09E-04	0.88332
	GOTERM_CC_DIRECT	Extracellular space	14	0.001122	1.25424
	GOTERM_CC_DIRECT	Stress fiber	4	0.001916	2.13231
	GOTERM_CC_DIRECT	Extracellular matrix	5	0.002899	3.21056
	GOTERM_BP_DIRECT	Transforming growth factor beta receptor signaling pathway	4	0.004864	6.85297
	GOTERM_BP_DIRECT	Positive regulation of release of cytochrome c from mitochondria	3	0.007117	9.87749
	GOTERM_CC_DIRECT	Focal adhesion	7	0.008523	9.17272
	GOTERM_BP_DIRECT	Positive regulation of cell migration	5	0.009217	12.6128

D.

	Category	Term	Gene count	P-value	FDR
Up-regulated	GOTERM_MF_DIRECT	ATP binding	80	6.78E-16	9.21E-13
	GOTERM_BP_DIRECT	DNA replication initiation	13	8.83E-14	1.44E-10
	GOTERM_BP_DIRECT	Chromosome segregation	14	2.48E-10	4.03E-07
	GOTERM_CC_DIRECT	Nuclear chromosome, telomeric region	18	2.65E-10	3.51E-07
	GOTERM_CC_DIRECT	Spindle pole	15	5.33E-10	7.05E-07
	GOTERM_CC_DIRECT	Midbody	17	6.23E-10	8.25E-07
	GOTERM_CC_DIRECT	Kinesin complex	13	4.65E-09	6.15E-06
	GOTERM_BP_DIRECT	Mitotic sister chromatid segregation	9	1.86E-08	3.02E-05
	GOTERM_BP_DIRECT	Microtubule-based movement	14	2.44E-08	3.98E-05
	GOTERM_CC_DIRECT	Kinetochore	13	2.55E-08	3.38E-05
Down-regulated	GOTERM_CC_DIRECT	Lysosome	12	1.39E-05	0.0172
	GOTERM_CC_DIRECT	Extracellular exosome	58	0.00181	2.21502
	GOTERM_BP_DIRECT	Regulation of cell shape	8	0.00306	4.76552
	GOTERM_BP_DIRECT	Positive regulation of fibroblast apoptotic process	3	0.00491	7.54745
	GOTERM_BP_DIRECT	Response to hypoxia	6	0.0091	13.554
	GOTERM_MF_DIRECT	Phosphatidylinositol binding	6	0.01062	13.4174
	GOTERM_BP_DIRECT	Androgen metabolic process	3	0.01136	16.6502
	GOTERM_BP_DIRECT	Negative regulation of growth	3	0.01136	16.6502
	GOTERM_BP_DIRECT	Positive regulation of endothelial cell differentiation	3	0.01136	16.6502
	GOTERM_BP_DIRECT	Regulation of stem cell population maintenance	3	0.01136	16.6502

E.

	Category	Term	Gene count	P-value	FDR
Up-regulated	GOTERM_BP_DIRECT	Retina vasculature morphogenesis in camera-type eye	2	0.01613	19.6781
	GOTERM_CC_DIRECT	Cell surface	5	0.02198	20.9497
	GOTERM_BP_DIRECT	Insulin-like growth factor receptor signaling pathway	2	0.02569	29.5769
	GOTERM_CC_DIRECT	Plasma membrane	11	0.04139	36.0558
	GOTERM_CC_DIRECT	Microtubule	3	0.04523	38.7122
	GOTERM_CC_DIRECT	Extracellular space	7	0.04575	39.0637
	GOTERM_BP_DIRECT	Positive regulation of smooth muscle cell proliferation	2	0.06301	58.3991
	GOTERM_BP_DIRECT	Regulation of multicellular organism growth	2	0.06606	60.1855
	GOTERM_CC_DIRECT	Intracellular	6	0.07053	53.8688
	GOTERM_BP_DIRECT	Response to heat	2	0.07212	63.5318
Down-regulated	GOTERM_CC_DIRECT	Collagen trimer	2	0.08399	57.2015
