# Supplementary material for: Evaluation of canine 2D cell cultures as models of myxomatous mitral valve degeneration
Source: PLoS One. 2019 Aug 15;14(8):e0221126. doi: 10.1371/journal.pone.0221126 (PMC6695117; doi:10.1371/journal.pone.0221126)
Supplement: S9 Table — Top five disease and function networks associated with the genes differentially expressed between A TGFβ1-qVICs/qVICs; B SB431542-aVICs/aVICs; C qVICs/aVICs; D TGFβ1-qVICs/aVICs. Underlined networks are shown schematically below. (PDF) [file pone.0221126.s009.pdf]

S9 Table. Disease and Function Annotations

Top five disease and function networks associated with the genes differentially expressed between A TGFβ1-qVICs/qVICs; B SB431542-aVICs/aVICs; C qVICs/aVICs; D TGFβ1-qVICs/aVICs. Underlined networks are shown schematically below.

A

**Top disease and function**
Cancer, Endocrine System Disorders, Organismal Injury and Abnormalities
Cellular Movement, Organismal Survival, Cancer
<u>Cellular Movement, Cardiac Arrythmia, Cardiovascular Disease</u>
Cancer, Organismal Injury and Abnormalities, Reproductive System Disease
Cardiac Enlargement, Cardiovascular Disease, Cardiovascular System Development and Function

B

**Top disease and function**
Dermatological Diseases and Conditions, Inflammatory Disease, Organismal Injury and Abnormalities
Drug Metabolism, Small Molecule Biochemistry, Cellular Assembly and Organization
<u>Cardiovascular Disease, Hereditary Disorder, Organismal Injury and Abnormalities</u>
Cell Morphology, Glomerular Injury, Organ Morphology
Developmental Disorder, Ophthalmic Disease, Organismal Injury and Abnormalities

C

**Top disease and function**
<u>Cell Cycle, Cellular Movement, Cancer</u>
Cell Cycle, Reproductive System Development and Function, Cellular Assembly and Organization
Cellular Assembly and Organization, Connective Tissue Disorders, Developmental Disorder
Cellular Assembly and Organization, DNA Replication, Recombination, and Repair, Cell Cycle
Cell Cycle, DNA Replication, Recombination, and Repair, Cancer

D

**Top disease and function**
<u>Cell Morphology, Cardiovascular Disease, Developmental Disorder</u>
Cancer, Endocrine System Disorders, Organismal Injury and Abnormalities
Connective Tissue Disorders, Organismal Injury and Abnormalities, Cell Death and Survival
Cellular Movement, Cellular Development, Cellular Growth and Proliferation
Cell-To-Cell Signaling, Hematological System Development and Function, Hematopoiesis
