# Supplementary figures and images for: Evaluation of canine 2D cell cultures as models of myxomatous mitral valve degeneration
Source: PLoS One. 2019 Aug 15;14(8):e0221126. doi: 10.1371/journal.pone.0221126 (PMC6695117; doi:10.1371/journal.pone.0221126)

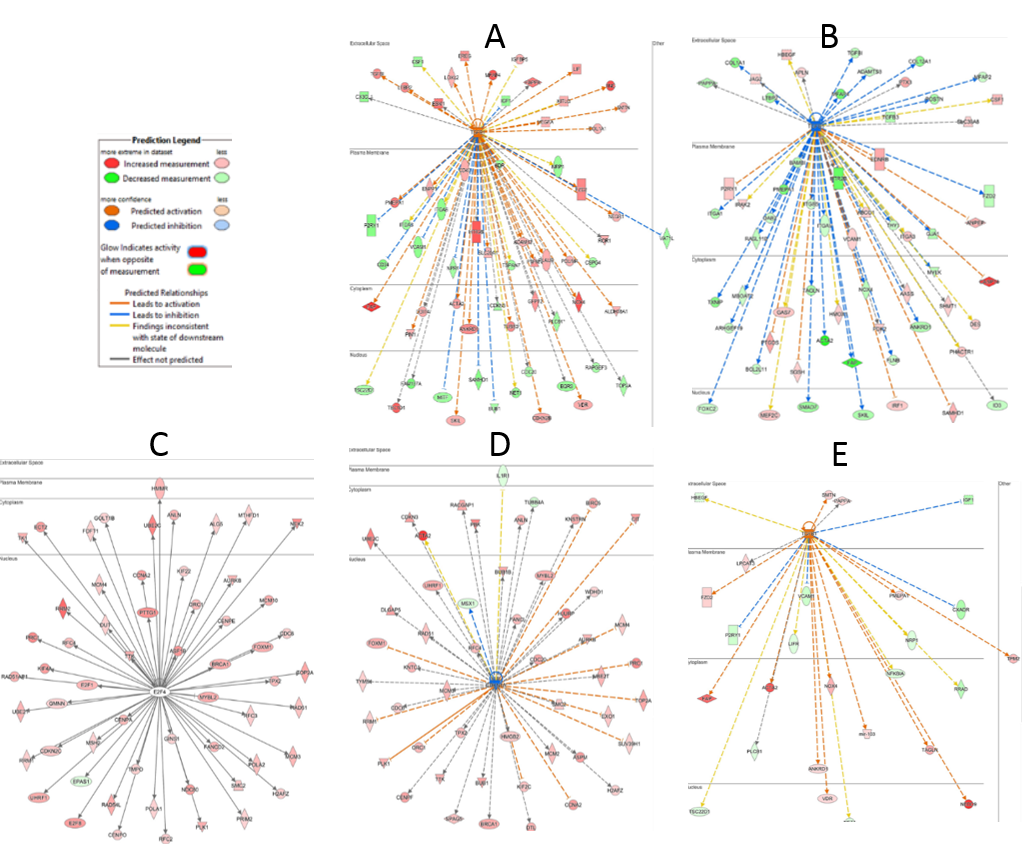

Supplement: S1 Fig — Networks of genes differentially expressed showing downstream effects from the extracellular space to the nucleus. Genes are coloured red and green to represent up- or down-regulation in the dataset. Dotted lines connecting the regulator to these genes show the expected effect of regulator signalling: orange—activation, blue—inhibition, yellow—result inconsistent and grey—effect not predicted. A. TGFβ1 network; TGFβ1-qVICs/qVICs; B. TGFβ1 network; SB431542-aVICs/aVICs; C. E2F4 network; aVICs/qVICs; D. CDKN1A network; aVICs/qVICs; E.TGFβ1 network; aVICs/TGFβ1-qVICs/. (TIF) [file pone.0221126.s010.tif]

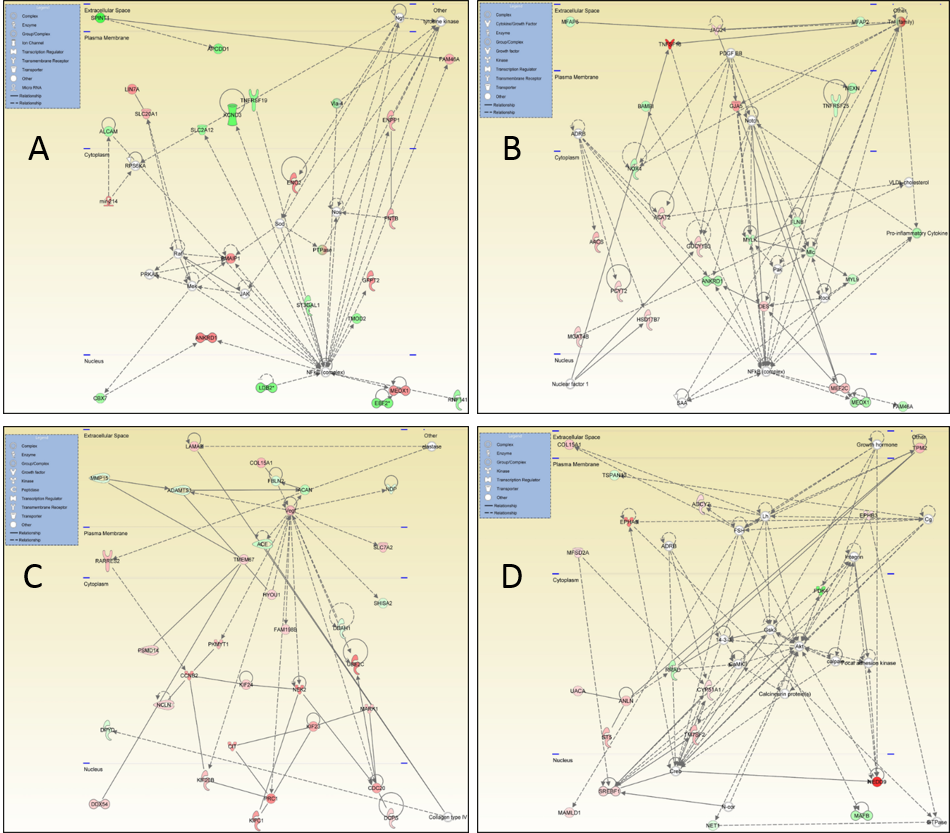

Supplement: S2 Fig — Genes are shown in their protein cellular location with red indicating up-regulation, green down-regulation and un-coloured showing no change in the dataset. A. Cellular movement, cardiac arrhythmia and cardiovascular disease; B. Cardiovascular disease, hereditary disorder, organismal injury and abnormalities; C. Cell cycle, cellular movement and Cancer; D. Cell morphology, cardiovascular disease and development disorder. (TIF) [file pone.0221126.s011.tif]
